# Author Correction: Geographics and bacterial networks differently shape the acquired and latent global sewage resistomes

**DOI:** 10.1038/s41467-026-74855-7

**Published:** 2026-07-01

**Authors:** Hannah-Marie Martiny, Patrick Munk, Alessandro Fuschi, Ágnes Becsei, Nikiforos Pyrounakis, Christian Brinch, Global Sewage Consortium, Hannah-Marie Martiny, Patrick Munk, Alessandro Fuschi, Ágnes Becsei, Nikiforos Pyrounakis, Christian Brinch, D. G. Joakim Larsson, Marion Koopmans, Nawel Zaatout, Catherine Rees, Guangming Jiang, Jiahua Shi, Bernhard Benka, Franz Allerberger, Sandra Koeberl-Jelovcan, Khalil Hasan Abdulla, Ali Bin Thani, Anowara Begum, Carlon Worrell, Tamegnon Victorien Dougnon, Freddy Soria, Natasa Mazalica, Teddie Rahube, Andreza Francisco Martins, Carlos Alberto Tagliati, Larissa Camila Ribeiro de Souza, Ariane Nzouankeu, Muhammad Attiq Rehman, Jeff Gauthier, Roger C. Levesque, Sean D. Workman, Christopher Yost, Aiko Adell Nakashima, Andres Opazo, Gerardo Gonzales, Yongjie Yu, Pilar Donado-Godoy, Tadjidine Youssouf, Pablo-César Rivera-Navarro, Matijana Jergovic, Jasna Hrenovic, Renata Karpiskova, Julien Kalpy Coulibaly, William Calero-Cáceres, Mohamed Abouelnaga, Anna-Maria Hokajärvi, Annamari Heikinheimo, Soizick Le Guyader, Andreas Nitsche, Annika Brinkmann, Sara Schubert, Thomas Berendonk, Uli Klümper, Ernest Bonah, Solomon Asante Sefa, Andrew Camilleri, Courage Kosi Setsoafia Saba, Victoria Bernice Sedor, Kassiani Mellou, Theologia Sideroglou, Charalampos Kotzamanidis, Jens-Peter B. Henriksen, Mie Møller, Thorunn Rafnar Thorsteinsdottir, A. A. Mohamed Hatha, Sima Mohammad, Burhan Shamurad, Hiwa Ali Faraj, Dearbháile Morris, Louise O’Connor, Jacob Moran-Gilad, Roberta Orletti, Giuseppina La Rosa, Marcello Iaconelli, Antonio Battisti, Lucia Decastelli, Maira Napoleoni, Alessandra De Cesare, Gianluca Corno, Jelena Avsejenko, Ghassan M. Matar, Christian Penny, Luc Hervé Samison, Daniel L. Banda, Heera Rajandas, Sivachandran Parimannan, Malcolm Vella Haber, Sunita J. Santchurn, Julian Carrillo-Reyes, Julián Osvaldo Ovis-Sánchez, René Arredondo-Hernández, Aleksandar Vujacic, Dijana Djurovic, Pushkar Pal, Carlos J. A. Campos, Isabelle Pattis, Stephen Chambers, Gert-Jan Jeunen, Charles Elikwu, Olayinka Osuolale, Mwapu Ndahi, Oluwadamilola Abiodun-Adewusi, Amen Ekhosuehi, Ayorinde Afolayan, Kayode Fashae, Mabel Kamweli Aworh, Akeem Olayiwola Ahmed, Ibrahim Adisa Raufu, Ismail Odetokun, Samaila Musa Chiroma, Claire Mitchell, Rune Holmstad, Natalie Weiler, Dariusz Wasyl, Magdalena Zając, Eugénia Cardoso, Célia Manaia, Maria Jorge Campos, Hor-Gil Hur, Min Joon Song, Sukhwan Yoon, Olga Burduniuc, Peiying Hong, Vladimir Radosavljevic, Angel Anika Cokro, Dagmar Gavacová, Marija Trkov, Rukayya Hussain Abubakar, Karen Keddy, Isabel Martínez-Alcalá, Marta Cerdà-Cuéllar, Ana Cañas, Fernando Gonzalez-Candelas, Yahia Ali Sabiel, Tanja van der Heijden, Yu-ping Hong, Julius John Medardus, Rajeev P. Nagassar, Cemíl Kürekci, Patrick McNamara, Lisa Wong, Erica Fuhrmeister, John Scott Meschke, Nicola Beck, Ayella Maile-Moskowitz, Jens Thomsen, Ouidiane Obaidi, Bruce Paterson, Anne Leonard, Lihong Zhang, Kevin Chau, Daniel Remondini, István Csabai, Frank M. Aarestrup, D. G. Joakim Larsson, Marion Koopmans, Daniel Remondini, István Csabai, Frank M. Aarestrup

**Affiliations:** 1 Research Group for Genomic Epidemiology, Technical University of Denmark, Lyngby, Denmark; 2 Department of Physics and Astronomy (DIFA), University of Bologna, Bologna, Italy; 3 Department of Physics of Complex Systems, ELTE Eötvös Loránd University, Budapest, Hungary; 4 Department of Infectious Diseases, Institute of Biomedicine, University of Gothenburg, and Centre for Antibiotic Resistance Research in Gothenburg, Gothenburg, Sweden; 5 Erasmus Medical Centre, Rotterdam, The Netherlands; 6 University of Batna 2, Batna, Algeria; 7 Melbourne Water Corporation, Melbourne, VIC Australia; 8 School of Civil, Mining, Environmental, and Architecture Engineering, University of Wollongong, Wollongong, NSW Australia; 9 University of Wollongong, Wollongong, NSW Australia; 10 Austrian Agency for Health and Food Safety (AGES), Vienna, Austria; 11 The Supreme Council for Environment (SCE) in Bahrain, Manama, Bahrain; 12 University of Bahrain, Sakhir, Bahrain; 13 University of Dhaka, Dhaka, Bangladesh; 14 Environmental Protection Department, Bridgetown, St. Michael, Barbados; 15 Polytechnic School of Abomey-Calavi, Abomey-Calavi, Benin; 16 Universidad Católica Boliviana San Pablo, La Paz, Bolivia; 17 Public Health Institute of the Republic of Srpska, Banja Luka, Bosnia and Herzegovina; 18 Botswana International University of Science and Technology, Palapye, Botswana; 19 Federal University of Rio Grande do Sul, Porto Alegre, Brazil; 20 Universidade Federal de Minas Gerais, Belo Horizonte, Brazil; 21 Centre Pasteur du Cameroun, Yaoundé, Cameroon; 22 Research and Productivity Council, Fredericton, NB Canada; 23 Université Laval, Québec, QC Canada; 24 University of Regina, Regina, SK Canada; 25 Escuela de Medicina Veterinaria, Facultad de Ciencias de la Vida, Universidad Andrés Bello, Santiago, Chile; 26 Universidad de Concepción, Concepción, Chile; 27 Nanjing University of Information Science and Technology, Nanjing, China; 28 Global Health Research Unit on Genomic Surveillance of Antimicrobial Resistance (GHRU), CI Tibaitatá, Corporación Colombiana de Investigación Agropecuaria (AGROSAVIA), Funza, Colombia; 29 Laboratoire National de Référence Hospitalier, Moroni, Comoros; 30 University of Costa Rica, San José, Costa Rica; 31 Andrija Stampar Teaching Institute of Public Health, Zagreb, Croatia; 32 University of Zagreb, Zagreb, Croatia; 33 Veterinary Research Institute, Brno, Czechia; 34 Institut Pasteur de Côte d’Ivoire, Abidjan, Côte d’Ivoire; 35 UTA-RAM-One Health, Group for Universal Advance in bioScience, Department of Food and Biotechnology Science and Engineering, Universidad Técnica de Ambato, Ambato, Ecuador; 36 Suez Canal University, Ismailia, Egypt; 37 Tampere University, Tampere, Finland; 38 University of Helsinki, Helsinki, Finland; 39 French Institute Search Pour L’exploitation De LaMer (Ifremer), Nantes, France; 40 Robert Koch Institute, Berlin, Germany; 41 Institute of Hydrobiology, Technische Universität Dresden, Dresden, Germany; 42 Food and Drugs Authority, Accra, Ghana; 43 Public Health Laboratory, Ghana Health Service, Takoradi, Ghana; 44 University for Development Studies, Tamale, Ghana; 45 Veterinary Services Department, Ministry of Food and Agriculture, National Food Safety Laboratory, Accra, Ghana; 46 National Public Health Organisation (EODY), Athens, Greece; 47 Veterinary Research Institute of Thessaloniki, Hellenic Agricultural Organisation-DEMETER, Thermi, Greece; 48 Kommuneqarfik Sermersooq, Nuuk, Greenland; 49 Peqqik, Nuuk, Greenland; 50 University of Iceland, Reykjavik, Iceland; 51 Cochin University of Science and Technology, Cochin, India; 52 Shahid Beheshti University, Tehran, Iran; 53 The University of Sulaimani, Sulaymaniyah, Iraq; 54 National University of Ireland, Galway, Ireland; 55 BenGurion University of the Negev and Ministry of Health, Beer-Sheva, Israel; 56 Ambiente, Marche, Italy; 57 Istituto Superiore di Sanità, Rome, Italy; 58 Istituto Zooprofilattico Sperimentale del Lazio e della Toscana, Rome, Italy; 59 Istituto Zooprofilattico Sperimentale del Piemonte, Liguria e Valle d’Aosta, Italy; 60 Istituto Zooprofilattico Sperimentale dell’Umbria e delle Marche “Togo Rosati”, Perugia, Italy; 61 University of Bologna, Bologna, Italy; 62 Water Research Institute, National Research Council (CNR), Verbania, Italy; 63 Institute of Food Safety, Riga, Latvia; 64 American University of Beirut, Beirut, Lebanon; 65 Luxembourg Institute of Science and Technology, Belvaux, Luxembourg; 66 University of Antananarivo, Centre d’Infectiologie Charles Mérieux, Antananarivo, Madagascar; 67 University of Malawi, Blantyre, Malawi; 68 AIMST University, COMBio, Kedah Malaysia; 69 Environmental Health Directorate, St. Venera, Malta; 70 University of Mauritius, Reduit, Mauritius; 71 Universidad Nacional Autónoma de México, Mexico City, Mexico; 72 Institute for Public Health Montenegro, Podgorica, Montenegro; 73 Agriculture and Forestry University, Kathmandu, Nepal; 74 Cawthron Institute, Nelson, New Zealand; 75 Institute of Environmental Science and Research Limited (ESR), Christchurch, New Zealand; 76 University of Otago, Christchurch, New Zealand; 77 University of Otago, Dunedin, New Zealand; 78 Babcock University Teaching Hospital, Ilishan-Remo, Nigeria; 79 Elizade University, Ilara-Mokin, Nigeria; 80 Federal Ministry of Agriculture and Rural Development, Abuja, Nigeria; 81 Nigeria Field Epidemiology Training Program, Abuja, Nigeria; 82 University of Benin, Benin City, Nigeria; 83 University of Ibadan, Ibadan, Nigeria; 84 University of Ilorin, Ilorin, Nigeria; 85 University of Maiduguri, Maiduguri, Nigeria; 86 Department of Agriculture, Environment and Rural Affairs, Northern Ireland, UK; 87 VEAS, Slemmestad, Norway; 88 Departamento de Bacteriologia, Laboratorio Central de Salud Publico, Asunción, Paraguay; 89 National Veterinary Research Institute, Pulawy, Poland; 90 Águas de Portugal, SGPS, S.A, Lisboa, Portugal; 91 Centre for Biotechnology and Fine Chemistry (CBQF), Universidade Católica Portuguesa, Porto, Portugal; 92 Escola Superior de Turismo e Tecnologia do Mar (ESTM), Instituto Politécnico de Leiria (IPLeiria), Peniche, Portugal; 93 Gwangju Institute of Science and Technology, Gwangju, Republic of Korea; 94 Korea Advanced Institute of Science and Technology, Daejeon, Republic of Korea; 95 National Agency for Public Health, Chișinău, Republic of Moldova; 96 King Abdullah University of Science and Technology, Thuwal, Saudi Arabia; 97 Institute of Veterinary Medicine of Serbia, Belgrade, Serbia; 98 Nanyang Technological University, Singapore, Singapore; 99 Public Health Authority of the Slovak Republic, Bratislava, Slovakia; 100 National Laboratory of Health, Environment and Food, Ljubljana, Slovenia; 101 Faculty of Veterinary Sciences, University of Pretoria, South Africa; 102 Independent consultant, Johannesburg, South Africa; 103 Catholic University of Murcia, Murcia, Spain; 104 Centre de Recerca en Sanitat Animal (CReSA), IRTA-UAB, Universitat Autònoma de Barcelona, Bellaterra, Spain; 105 Instituto de Salud Carlos III, Madrid, Spain; 106 Universidad de Valencia, Valencia, Spain; 107 Sudan University of Science and Technology, Khartoum, Sudan; 108 Ara Region Bern AG, Herrenschwanden, Switzerland; 109 Centers for Disease Control, Taipei, Taiwan, ROC; 110 Sokoine University of Agriculture, Morogoro, Tanzania; 111 Eastern Regional Health Authority (ERHA), Sangre Grande, Trinidad and Tobago; 112 Mustafa Kemal Üniversitesi, Hatay, Turkey; 113 Marquette University, Milwaukee, WI USA; 114 Massachusetts Water Resources Authority, Boston, MA USA; 115 Tufts University, Medford, MA USA; 116 University of Washington, Seattle, WA USA; 117 Virginia Tech, Blacksburg, VA USA; 118 Abu Dhabi Public Health Center, Abu Dhai, United Arab Emirates; 119 Dubai Municipality, WWTP Al Aweer, Dubai, United Arab Emirates; 120 Scottish Environment Protection Agency, Stirling, UK; 121 University of Exeter Medical School, Cornwall, UK; 122 University of Oxford, Oxford, UK

**Keywords:** Antimicrobial resistance, Network topology, Metagenomics, Microbial ecology, Policy and public health in microbiology

Correction to: *Nature Communications* 10.1038/s41467-025-66070-7, published online 21 November 2025

In this article, the Global Sewage Consortium member Julián Osvaldo Ovis-Sánchez was incorrectly written as Julian Osvaldo Sanchez-Lara. The original article has been updated.

