# Supplementary material for: Geographics and bacterial networks differently shape the acquired and latent global sewage resistomes
Source: Nat Commun. 2025 Nov 21;16:10278. doi: 10.1038/s41467-025-66070-7 (PMC12639157; doi:10.1038/s41467-025-66070-7)
Supplement: Supplementary file 1 — Supplementary Information [file 41467_2025_66070_MOESM1_ESM.pdf]

Geographics and bacterial networks differently shape the acquired and latent global sewage resistomes

Supplementary material

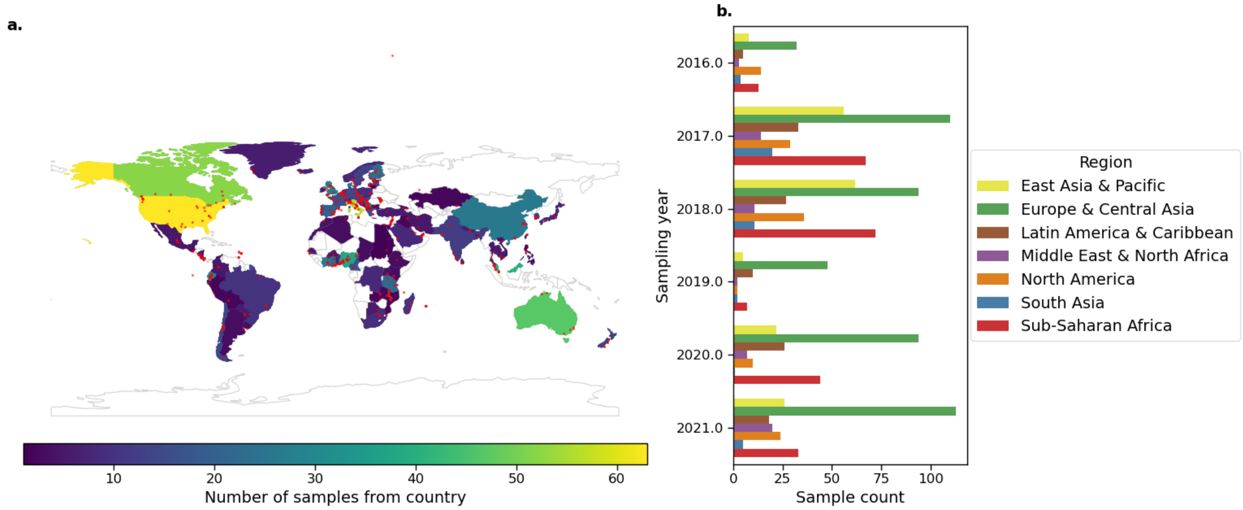

*Supplementary Figure 1. **Global Sewage samples included in this study**. **a**. Each location contributing untreated municipal sewage is marked as red, and each country is colored according to the number of samples received from that country. Country shapes were drawn using the Natural Earth dataset. **b**. The number of samples retrieved across the years from the different world regions.*

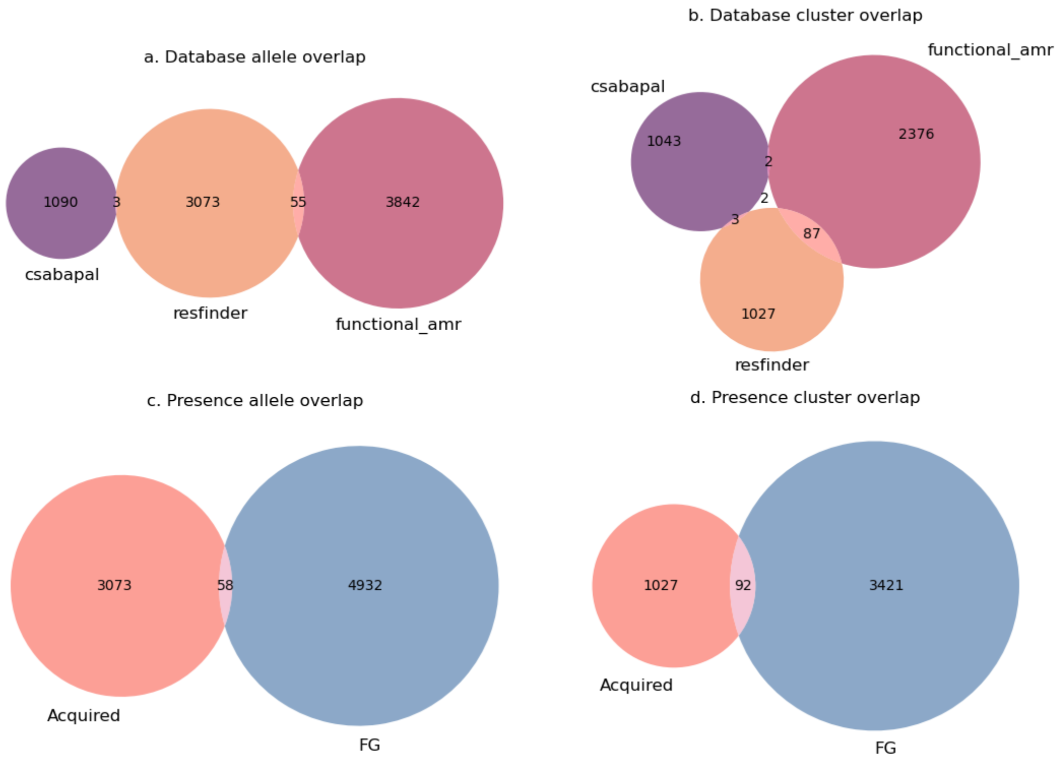

Supplementary Figure 2. ***The number of ARGs shared between the three different gene collections****.* ***a****. Amount of shared reference sequences between the three collections: ResFinder*[1]*, ResFinderFG*[2] *(functional_amr), and the genes from Daruka et al. (2025)*[3] *(csabpal).* ***b.*** *The number of shared 98% homology clusters.* ***c****. The alleles shared between the two groupings of ARGs: Acquired ARGs and FG ARGs.* ***d.*** *The number of shared 98% homology clusters between the two groupings.*

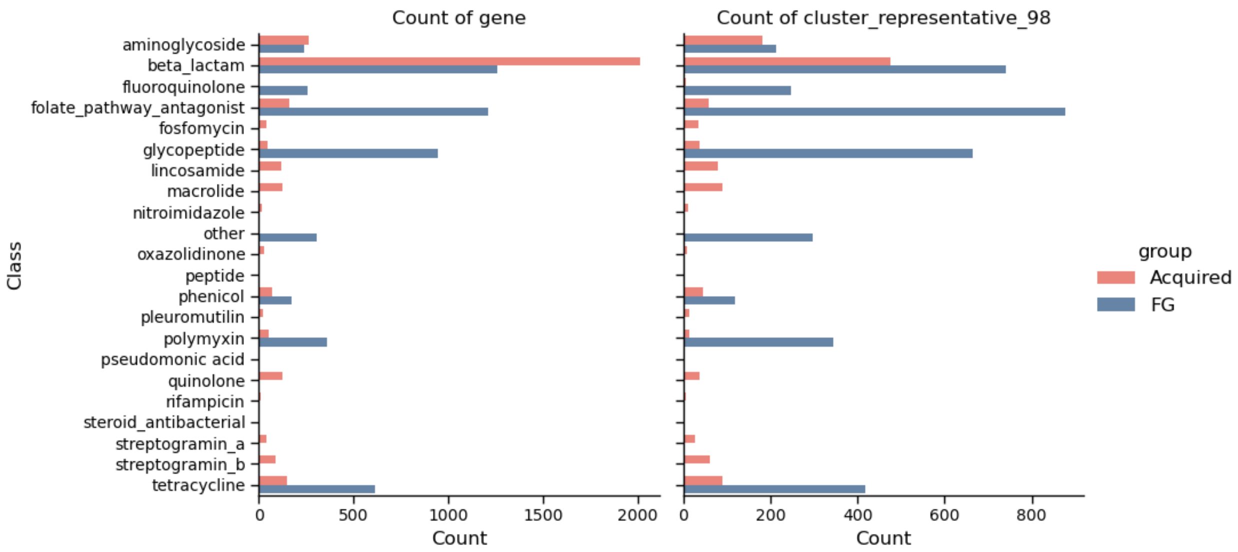

Supplementary Figure 3. ***The number of a. reference sequences and b. 98% homology clusters per class of antimicrobial resistance****. Note here that a sequence can be counted multiple times, as some ARGs confer resistance to multiple classes.*

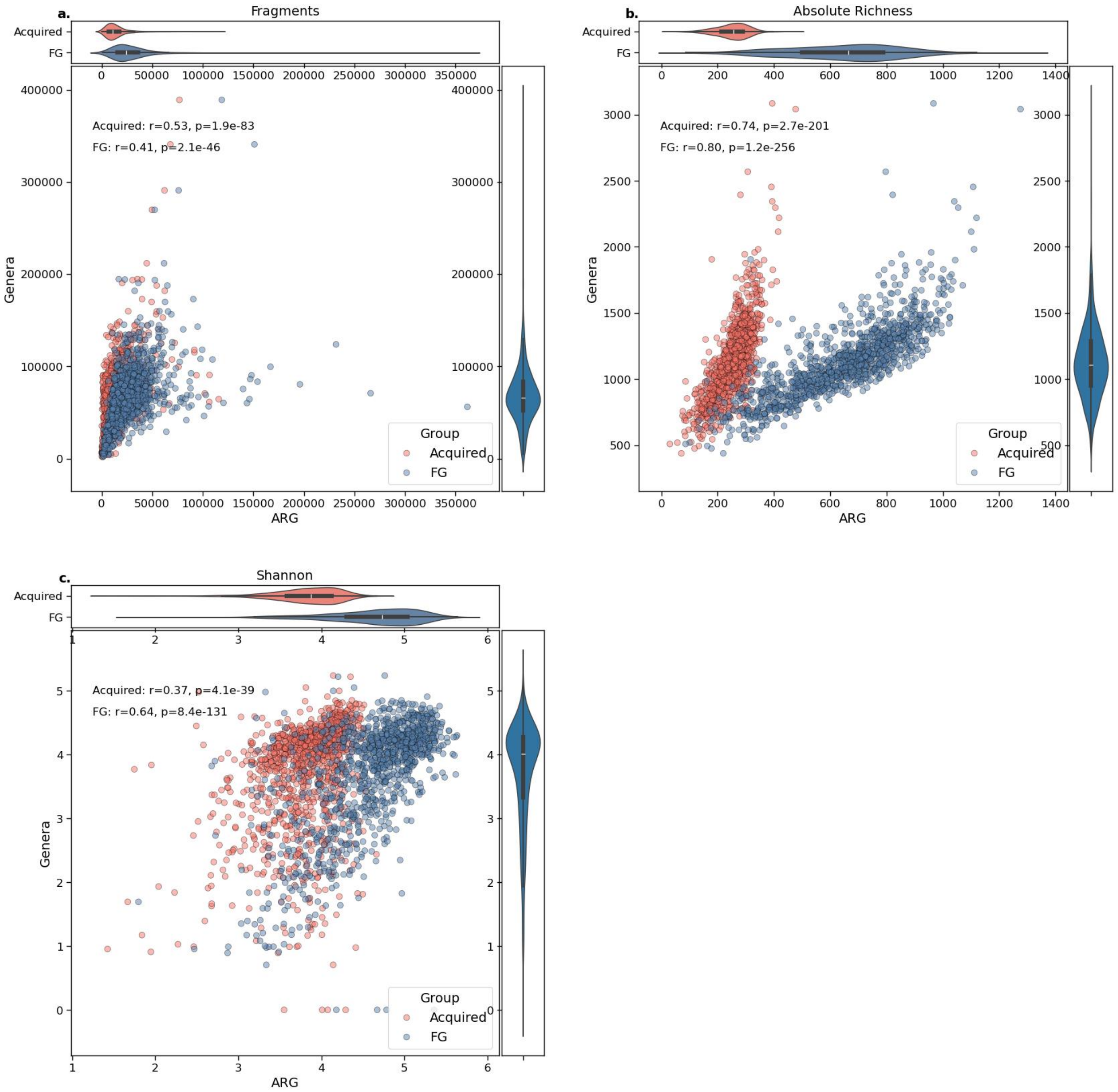

Supplementary Figure 4. ***Alpha-diversity indices of antimicrobial resistance genes (x-axis) and the bacterial genera (y-axis) found in the sewage samples*****.** ***a****. The number of read fragments aligned to either the Acquired ARGs or the FG ARGs and fragments aligned to genera.* ***b****. Absolute Richness of the ARGs and genera.* ***c.*** *Shannon diversity indices for ARGs and bacterial genera.*

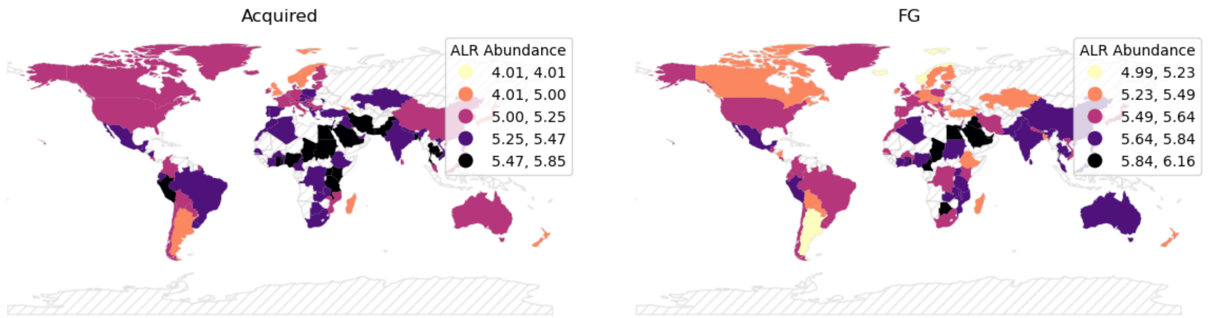

Supplementary Figure 5. ***Chloropleth world maps showing the country-wise ALR abundances*** *of acquired ARGs (left) and FG ARGs (right). If a country is hashed out, there is no data from there. Country shapes were drawn using the Natural Earth dataset.*

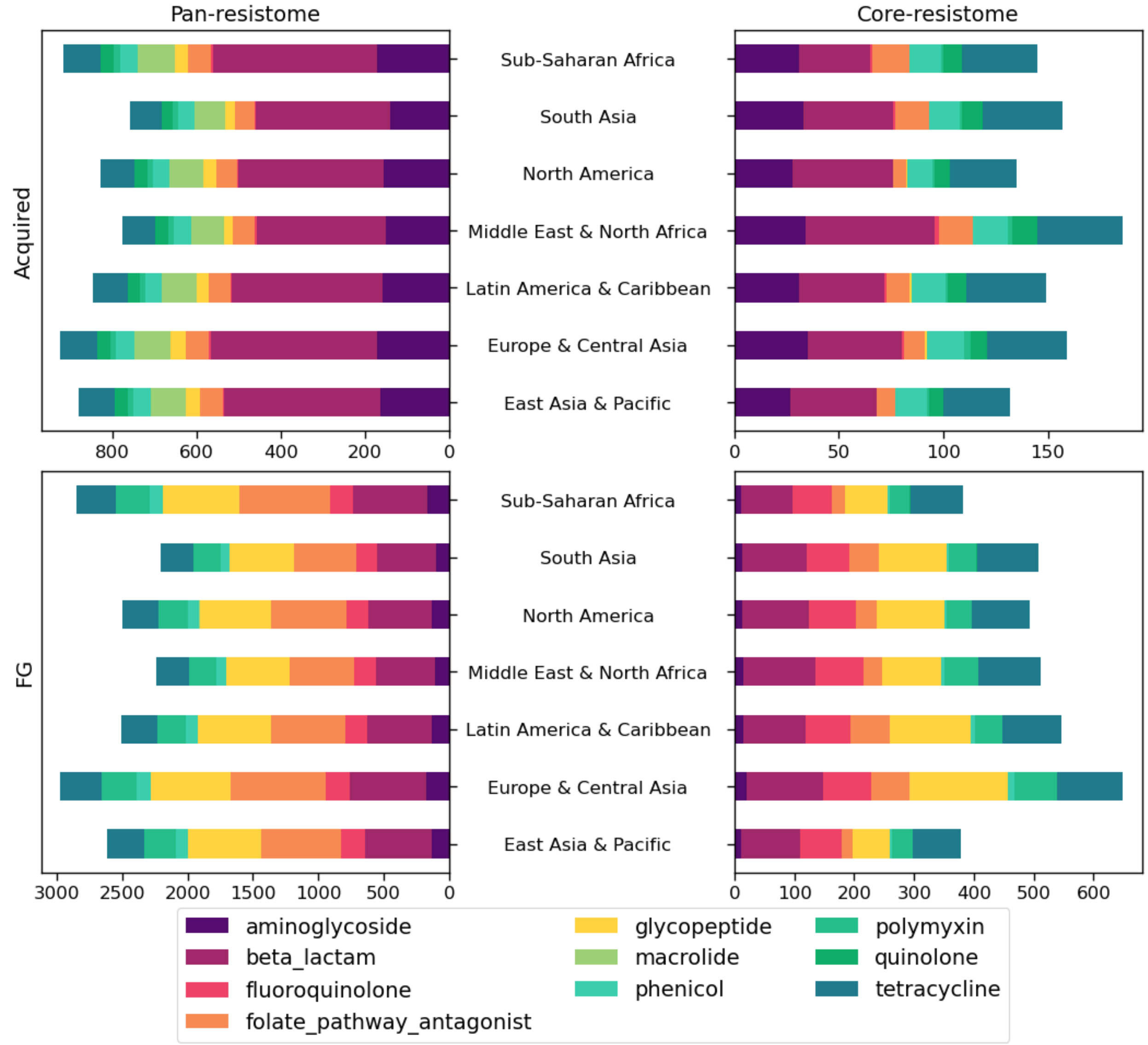

Supplementary Figure 6. ***Pan- and core-resistomes grouped by world region and colored by antimicrobial resistance class that are part of both the acquired and the FG collections****. The length of each bar indicates the number of genes matched with at least one read and is colored by the class of antimicrobial. The pan-resistome comprises genes detected in at least one sample from a global region, whereas the core-resistome encompasses genes present in at least 50% of the samples.*

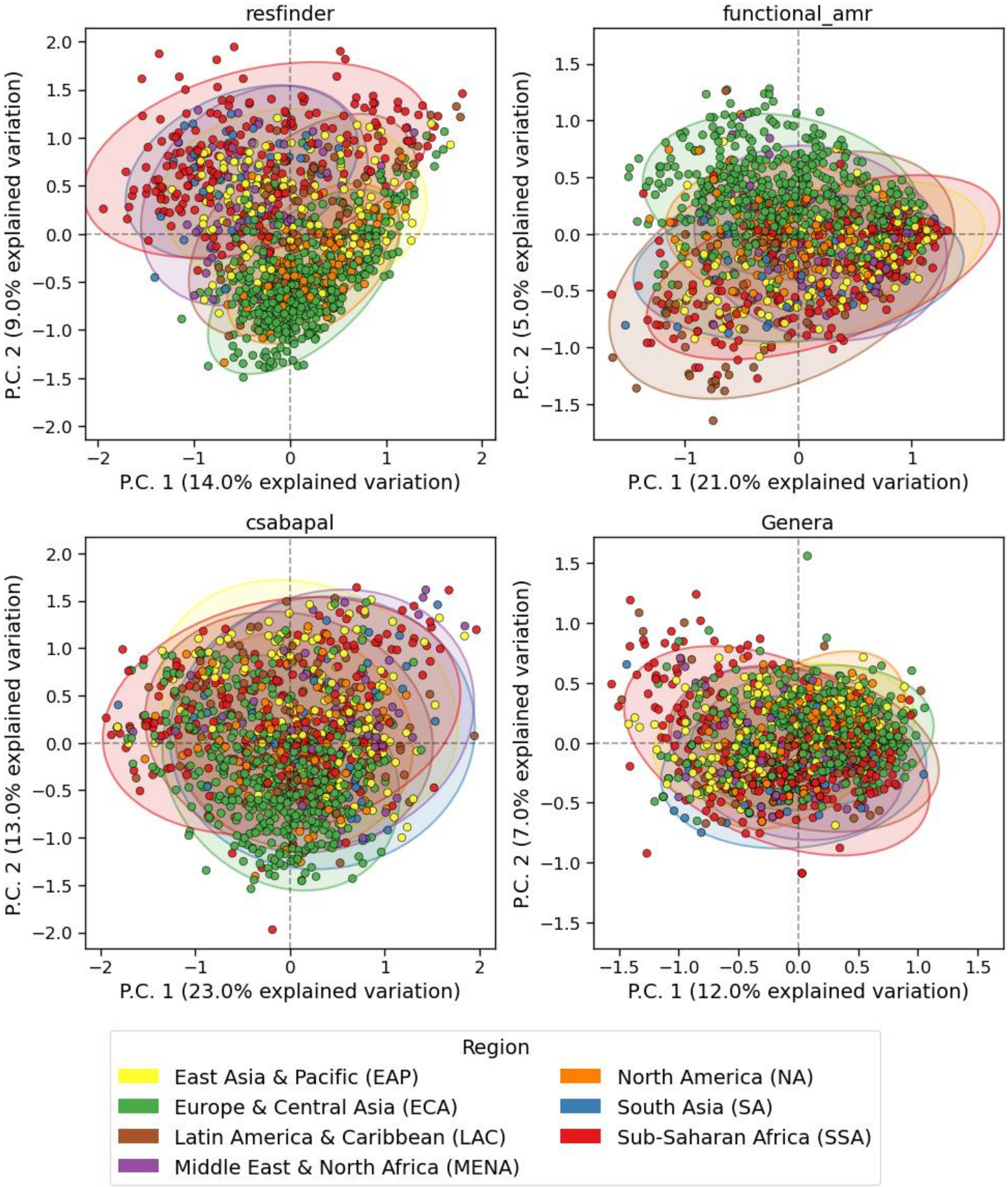

Supplementary Figure 7. ***Additional biplots visualizing the PCA scores of the sewage samples*** *based on the individual reference collections: ResFinder[1], ResFinderFG[2] (functional_amr), the Daruka et al. (2025)[3] genes (csabapal), and the bacterial genera from the mOTUs database[4]. Each sewage sample is colored by the world region from which it was taken.*

Resistance class	Acquired R^2	FG R^2
All	0.120 (*P*=0.001)	0.074 (*P*=0.001)
aminoglycoside	0.121 (*P*=0.001)	0.047 (*P*=0.001)
beta_lactam	0.116 (*P*=0.001)	0.082 (*P*=0.001)
fluoroquinolone	0.053 (*P*=0.001)	0.068 (*P*=0.001)
folate_pathway_antagonist	0.169 (*P*=0.001)	0.059 (*P*=0.001)
fosfomycin	0.072 (*P*=0.001)	
glycopeptide	0.026 (*P*=0.001)	0.078 (*P*=0.001)
lincosamide	0.087 (*P*=0.001)	
macrolide	0.093 (*P*=0.001)	
nitroimidazole	0.144 (*P*=0.001)	
oxazolidinone	0.106 (*P*=0.001)	
phenicol	0.140 (*P*=0.001)	0.073 (*P*=0.001)
pleuromutilin	0.093 (*P*=0.001)	
polymyxin	0.074 (*P*=0.001)	0.067 (*P*=0.001)
pseudomonic_acid	0.010 (*P*=0.084)	
quinolone	0.106 (*P*=0.001)	
rifampicin	0.163 (*P*=0.001)	
steroid_antibacterial	0.003 (*P*=0.77)	
streptogramin_a	0.071 (*P*=0.001)	
streptogramin_b	0.083 (*P*=0.001)	
tetracycline	0.073 (*P*=0.01)	0.110 (*P*=0.001)

Supplementary Table 1. ***Degree and significance of clustering ARG abundances and world regions*** *obtained by running Permutational Multivariate Analysis of Variance (permanova, adonis2) on CLR values grouped by the resistance class. The test was two-sided. The empty cells in the FG column indicate that none of the FG ARGs conferred resistance to that class.*

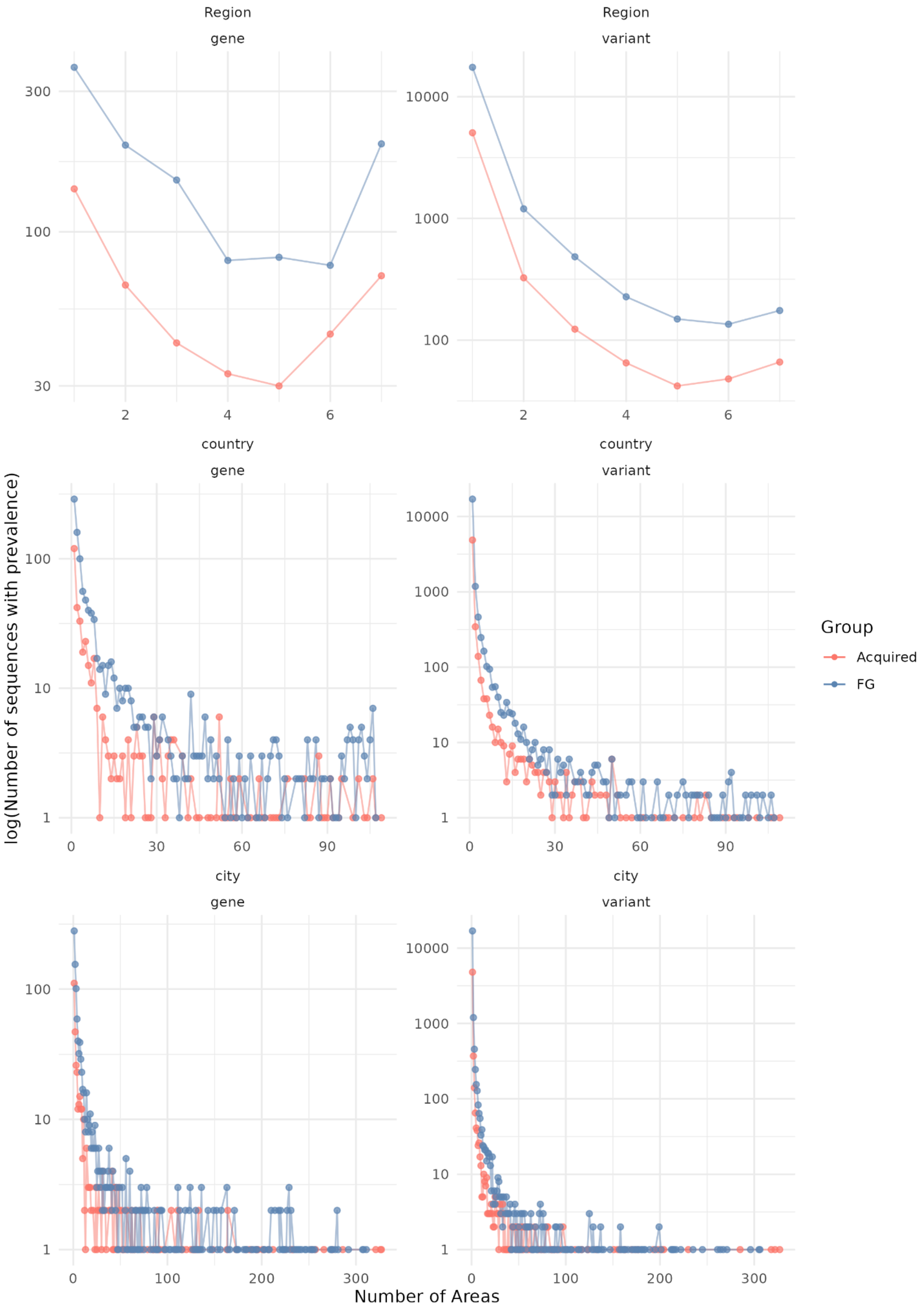

Supplementary Figure 8. ***More ARGs than expected are truly global****, as seen by their spread across geographical groupings: top: region, middle: country, bottom: city. The left column is on the level of the closest match to a reference in the assemblies, and the right column is the exact variant reported by Flankophile[5]. The y-axis has been scaled with log10.*

Model		Abundance			Assembly variants	
		**Acquired**	**FG**	**Genera**	**Acquired**	**FG**
Scale Independent	R^2	0.04	0.012	0.0065	0.27	0.089
	Slope	-0.036*** (*P*<<0)	-0.02*** (*P*=3.8e-155)	-0.017*** (*P*=9.5e-88)	-0.12*** (*P*=3.01e-322)	-0.071*** (*P*=0)
	Mantels *r*	0.22** (*P*=0.001)	0.12** (*P*=0.001)	0.091** (*P*=0.001)	0.6** (*P*=0.001)	0.32** (*P*=0.001)
Within Country	R^2	0.013	0.015	0.057	0.32	0.26
	Slope	-0.016** (*P*=0.0003)	-0.016** (*P*=0.0002)	-0.033*** (*P*=2.2e-13)	-0.064*** (*P*=6e-06)	-0.08*** (*P*=5.6e-15)
	Mantels *r*	0.14** (*P*=0.001)	0.15** (*P*=0.001)	0.26** (*P*=0.001)	0.57** (*P*=0.001)	0.49** (*P*=0.001)
Within Region	R^2	0.038	0.0051	0.00072	0.25	0.074
	Slope	-0.044*** (*P*=2.5e-100)	-0.017*** (*P*=4.2e-15)	-0.0072* (*P*=0.0021)	-0.089*** (*P*=4e-66)	-0.089*** (*P*=4e-66)
	Mantels *r*	0.2** (*P*=0.001)	0.079** (*P*=0.001)	0.034** (*P*=0.001)	0.51** (*P*=0.001)	0.27** (*P*=0.001)
Between Regions	R^2	0.00033	0.00041	0.0014	0.00044	0.035
	Slope	0.006*** (*P*=4.2e-05)	0.0069*** (*P*=6.7e-06)	0.015*** (*P*=2e-16)	-0.01 (*P*=0.11)	-0.084*** (*P*=1.7e-102)
	Mantels *r*	-0.02 (*P*=1)	-0.025 (*P*=1)	-0.044 (*P*=1)	0.013 (*P*=0.22)	0.19** (*P*=0.001)

Supplementary Table 2. **Results of the linear regression models and statistical tests to investigate the association between resistome similarity and sampling distances.** Mantel two-sided tests were performed between dissimilarity and distance matrices, and the distance-decay models used ln-transformed similarity matrices (i.e., 1-dissimilarity). Dissimilarities were measured either by Aitchison distances for the Abundance models or Bray-Curtis distances for the assembly variant models. Asterisks: * *P* ≤ 0.01, ** *P* ≤ 0.001, and *** *P* ≤ 0.0001.

Data input	Group	Comparison	Estimated Difference	Std. Error	t-ratio	*P*-value
Abundance	Acquired	Within Country - Within Region	0.0282	0.0038	7.4643	2.71e-13***
		Within country - Between Regions	-0.0216	0.0036	-6.0431	4.53e-09***
		Within region - Between Regions	-0.04978	0.0024	-20.4194	0.0e+00***
	FG	Within Country - Within Region	0.0010	0.0040	0.2520	9.66e-01
		Within country - Between Regions	-0.0227	0.0038	-6.0289	4.95e-09***
		Within region - Between Regions	-0.0237	0.0026	-9.2234	3.34e-14***
	Bacteria	Within Country - Within Region	-0.0259	0.0045	-5.7449	2.76e-08***
		Within country - Between Regions	-0.0476	0.0043	-11.1680	8.66e-15***
		Within region - Between Regions	-0.0217	0.0029	-7.4605	2.78e-13***
Assembly variants	Acquired	Within Country - Within Region	0.0247	0.0178	1.3906	3.46e-01
		Within country - Between Regions	-0.0538	0.0175	-3.0711	6.08e-03*
		Within region - Between Regions	-0.0785	0.0089	-8.8021	3.90e-08***
	FG	Within Country - Within Region	-0.0146	0.0107	-1.3631	3.60e-01
		Within country - Between Regions	0.00390	0.0103	0.3777	9.24e-01
		Within region - Between Regions	0.0185	0.0060	3.0965	5.57e-03*

Supplementary Table 3. ***Pairwise differences between the slopes of the linear regression models for distance-decays presented in Supplementary Table 2.*** *The estimated difference represents the contrast between the slopes of two spatial scales, along with associated standard errors, test statistics (t-ratios), and the p-value indicating the significance of each comparison.* Asterisks: * $P \leq 0.01$, ** $P \leq 0.001$, and *** $P \leq 0.0001$.

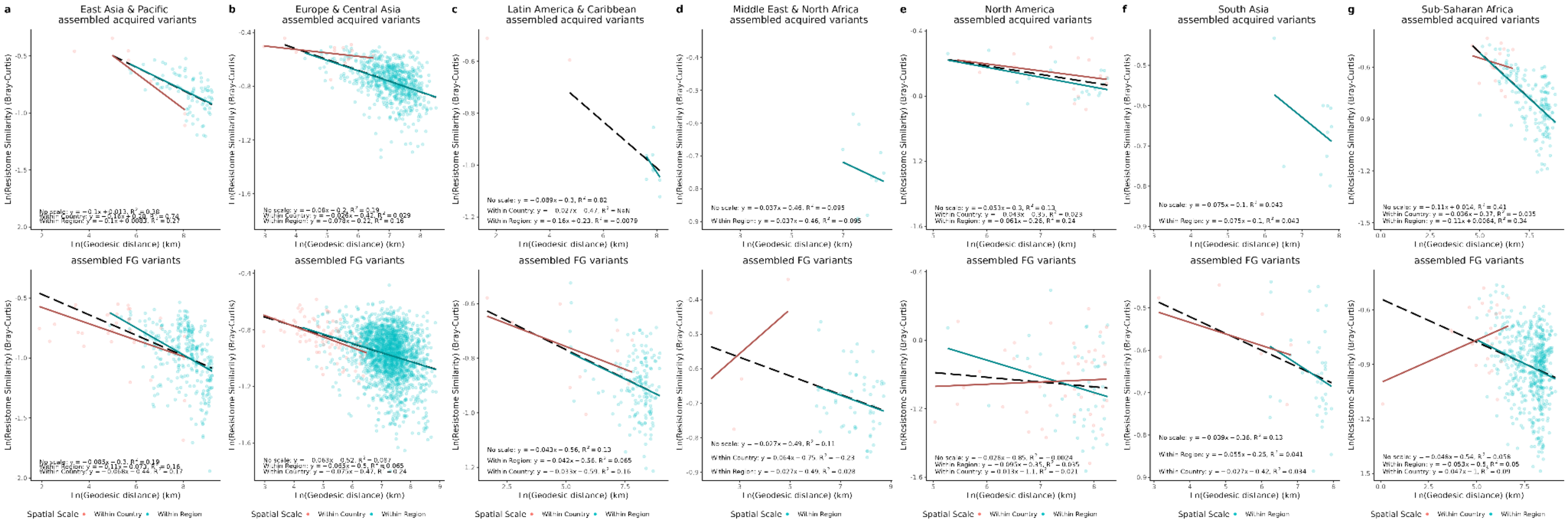

*Supplementary Figure 9. **The distance-decay relationships of the assembled variant resistomes for each world region**. On the x-axis are the pairwise city distances (in kilometers, km) and the resistome beta-diversity Bray-Curtis similarities. The regression lines represent linear models fitted to the ln-transformed values, where the dashed line indicates the fit across all spatial scales. The red line represents the fit for samples originating within the same country, and the blue line represents the fit for distances between countries. Two-sided t-tests were used to assess pairwise differences in slopes. No adjustments were made for multiple comparisons.*

Region	Collection	Scale Independent			Within Country			Within Region		
		R^2	Slope	Mantels *r*	R^2	Slope	Mantels *r*	R^2	Slope	Mantels *r*
EAP	Acquired	0.38	-0.1*** (*P*=3.5e-11)	0.69** (*P*=0.001)	0.74	-0.16* (*P*=0.0081)	0.86* (*P*=0.0042)	0.27	-0.1*** (*P*=2.1e-07)	0.56** (*P*=0.001)
	FG	0.19	-0.085*** (*P*=1.2e-17)	0.47** (*P*=0.001)	0.17	-0.068* (*P*=0.0091)	0.36** (*P*=0.001)	0.16	-0.11*** (*P*=8.4e-14)	0.42** (*P*=0.001)
ECA	Acquired	0.19	-0.08*** (*P*=2.6e-36)	0.47** (*P*=0.001)	0.029	-0.026 (*P*=0.27)	0.36* (*P*=0.006)	0.16	-0.078*** (*P*=1.1e-29)	0.43** (*P*=0.001)
	FG	0.087	-0.063*** (*P*=1.1e-44)	0.31** (*P*=0.001)	0.24	-0.075*** (*P*=2.6e-06)	0.48** (*P*=0.001)	0.065	-0.065*** (*P*=3.3e-32)	0.23** (*P*=0.001)
LAC	Acquired	0.82	-0.089** (*P*=2e-04)	0.95* (*P*=0.0083)	-	-	-	-0.008	-0.16 (*P*=0.37)	0.39 (*P*=0.12)
	FG	0.13	-0.043*** (*P*=6.8e-07)	0.42** (*P*=0.001)	0.16	-0.033 (*P*=0.12)	0.48* (*P*=0.009)	0.065	-0.042** (*P*=0.00068)	0.29** (*P*=0.001)
MENA	Acquired	-0.095	-0.037 (*P*=0.65)	0.22 (*P*=0.32)	-	-	-	-0.095	-0.037 (*P*=0.65)	0.22 (*P*=0.32)
	FG	0.11	-0.027* (*P*=0.0079)	0.4 (*P*=0.012)	-0.23	0.064 (*P*=0.58)	-0.67 (*P*=1)	0.028	-0.027 (*P*=0.12)	0.25 (*P*=0.035)
NA	Acquired	0.13	-0.053 (*P*=0.016)	0.39 (*P*=0.017)	0.023	-0.043 (*P*=0.26)	0.23 (*P*=0.11)	0.24	-0.061 (*P*=0.016)	0.42* (*P*=0.008)
	FG	-0.002	-0.028 (*P*=0.38)	0.089 (*P*=0.2)	-0.021	0.013 (*P*=0.73)	-0.029 (*P*=0.61)	0.035	-0.095 (*P*=0.11)	0.21 (*P*=0.039)
SA	Acquired	0.043	-0.075 (*P*=0.27)	0.47 (*P*=0.12)	-	-	-	0.043	-0.075 (*P*=0.27)	0.47 (*P*=0.12)
	FG	0.13	-0.039* (*P*=0.0081)	0.4* (*P*=0.006)	0.034	-0.027 (*P*=0.32)	0.49 (*P*=0.038)	0.041	-0.055 (*P*=0.12)	0.31 (*P*=0.016)

SSA	Acquired	0.41	-0.11*** (*P*=9.1e-24)	0.69** (*P*=0.001)	-0.035	-0.036 (*P*=0.48)	0.19 (*P*=0.1)	0.34	-0.11*** (*P*=1.4e-17)	0.64** (*P*=0.001)
	FG	0.058	-0.048*** (*P*=1.5e-09)	0.28** (*P*=0.001)	0.09	0.047 (*P*=0.095)	-0.31 (*P*=1)	0.05	-0.053*** (*P*=3.8e-08)	0.27** (*P*=0.001)

Supplementary Table 4. **Results of the regional linear regression models between resistome similarity in assembled variants and sampling distances**. The distance-decay models used ln-transformed similarity matrices (1-dissimilarity) and the physical distances between sampling points. Dissimilarities were measured by Bray-Curtis distances. Two-sided t-tests were used to assess pairwise differences in slopes. No adjustments were made for multiple comparisons. Asterisks: * $P \leq 0.01$, ** $P \leq 0.001$, and *** $P \leq 0.0001$. EAP: East Asia & Pacific, ECA: Europe & Central Asia, LAC: Latin America & Caribbean, MENA: Middle East & North Africa. NA: North America, SA: South Asia, SSA: Sub-Saharan Africa.

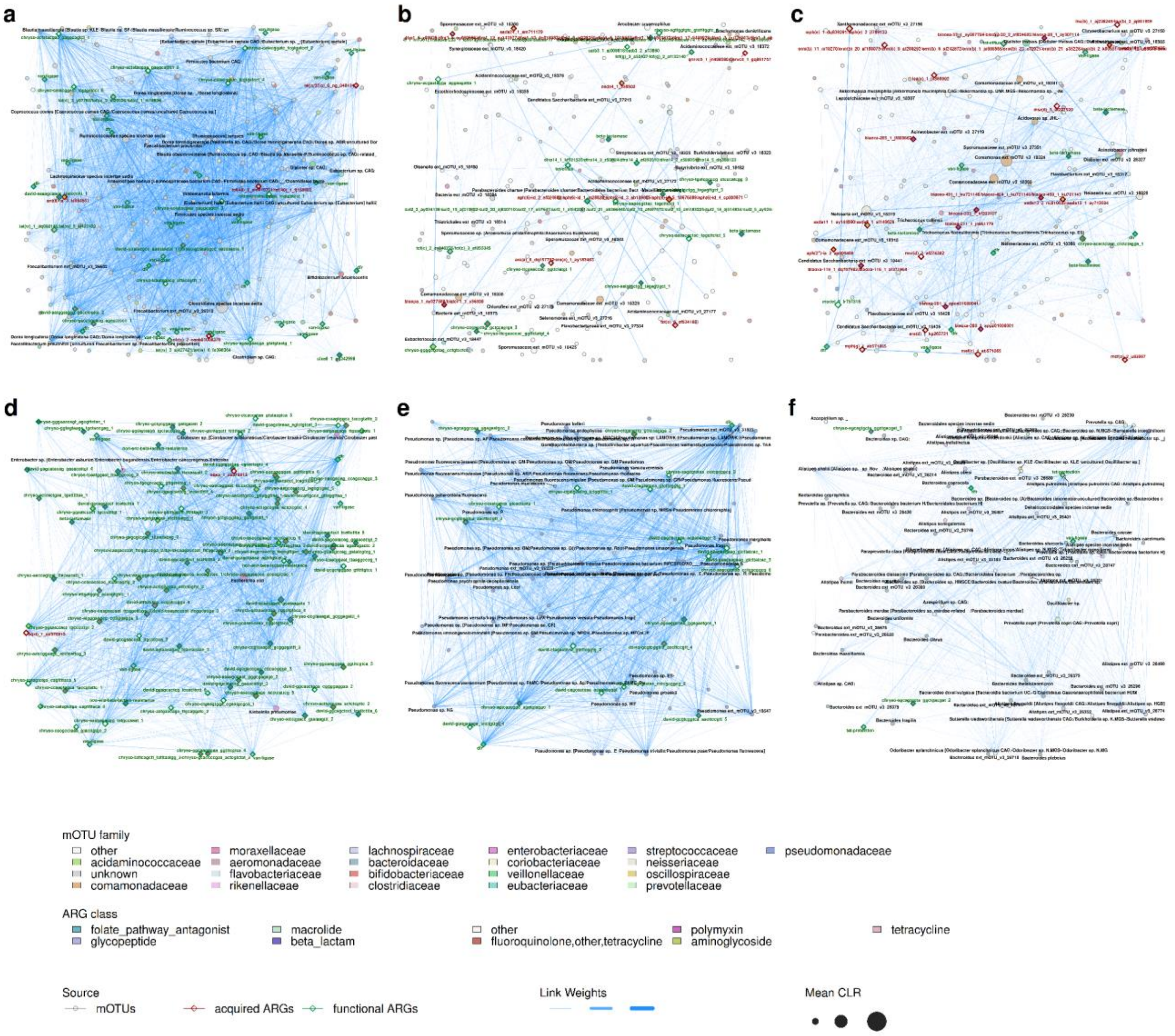

Supplementary Figure 10. ***Detailed visualization of microbial community networks.*** *Granular view of the composition and connectivity within seven distinct microbial communities, identified as Communities 1 through 6. Each panel (a-g) depicts the community structure, emphasizing the relationships and node characteristics such as microbial taxa (mOTUs) and antibiotic resistance genes (ARGs). Node size represents the mean CLR abundances, node color indicates the type of element (mOTUs, acquired ARGs, FG ARGs), and edge transparency for interaction strengths. In this representation, nodes are intentionally spaced farther apart to accommodate the inclusion of more labels, which alters the visual representation of the network's topology.*

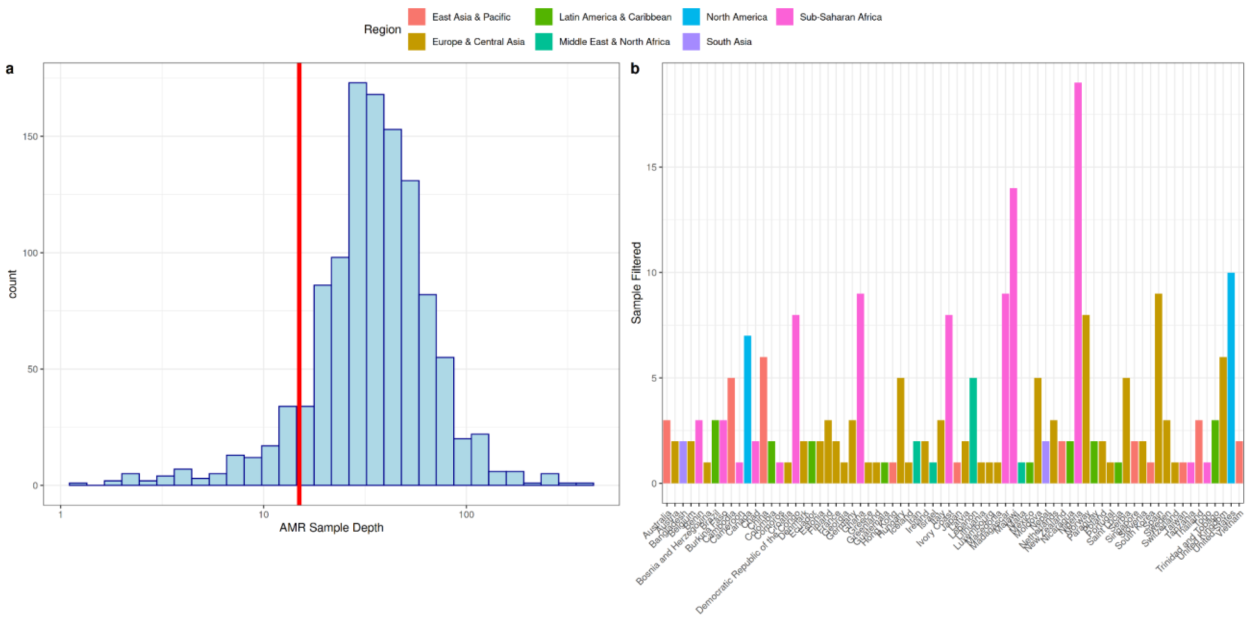

Supplementary Figure 11. ***Filtering Summary For Network Reconstruction. a****. Distribution of ARG samples on a logarithmic scale, with the red line indicating the threshold for inclusion.* ***b****. Details of the excluded samples by region and country.*

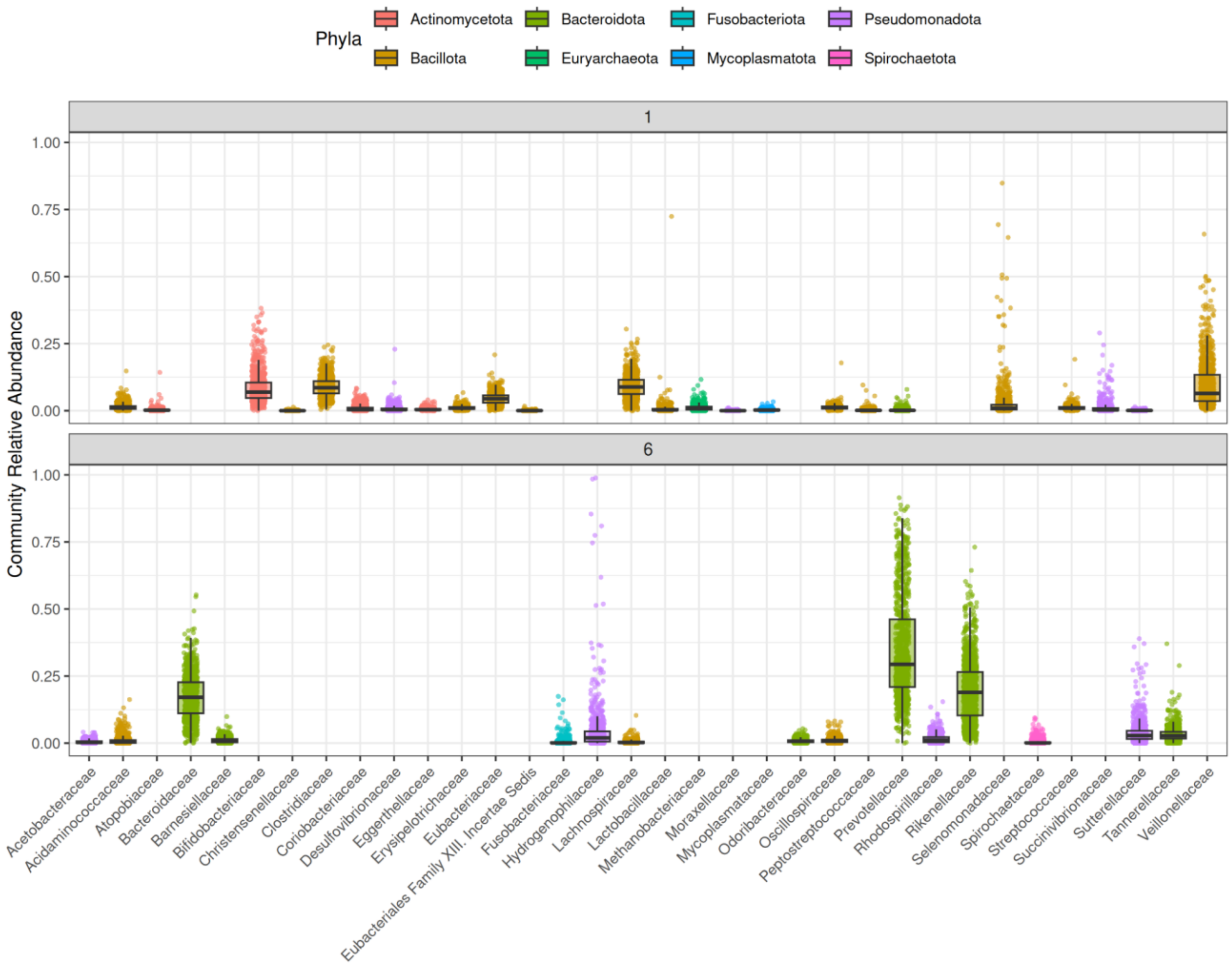

Supplementary Figure 12. ***Microbial Family Abundance in Human-Associated Communities 1 and 6.*** *Composition of microbial families within communities 1 and 6, with each family's relative abundance normalized by the total mOTU abundance within the respective community rather than the entire sample. Colors indicate phylum-level taxonomic differences, emphasizing the distinct microbial profiles. Only bacterial taxa are considered.*

Supplementary note on PanRes ARGs

We have compiled this supplementary note to address our decision to focus solely on a specific subset of the PanRes database, specifically the genes from the databases ResFinder[1], ResFinderFG[2], and the CsabaPal[3] collections. Concerns were raised by the reviewers as how the results were affected by our choice of excluding PanRes ARGs that weren't part of the target databases.

In this material, we begin by explaining the structure of the PanRes collection and the reasons for dividing a subset of the ARGs into acquired and functional metagenomic (FG) genes. Next, we expand the primary analyses of the manuscript by including the other excluded ARGs, which we refer to as the "other ARGs". Finally, we discuss how the exclusion of specific genes may have affected our main conclusions.

The content of PanRes

We introduced the idea of PanRes in our ARGprofiler manuscript[6] and made the full collection publicly available on Zenodo (https://zenodo.org/records/13885013). In brief, the PanRes collection integrates multiple existing resistance gene reference databases into a single, non-redundant resource, thereby eliminating the need to select which resistance reference database to use. Specifically, we included genes from the sources listed in Supplementary Table 5.

As shown in Supplementary Table 5, only the three databases ResFinder, ResFinderFG, and CsabaPal do not contain ARGs identified through similarity predictions. While overlaps exist across all the different database sources (Supplementary Figure 13), the functional metagenomic collections are mostly unique (Supplementary Figure 14).

Our study aimed to investigate the latent reservoir of resistance genes in sewage resistomes, which may represent a collection of ARGs that may become mobilized in the future. To achieve this, we focused on ARGs known to be mobilized or acquired, as well as those identified through functional metagenomics, which we hypothesize to be intrinsic resistance genes. We grouped the ARGs into these categories:

- Acquired ARGs: Genes known to be mobilized and transferred between species. We used ResFinder genes for this group, as this collection is explicitly curated for acquired resistance[1]23/10/2025 11:38:00.
- FG ARGs: ARGs identified with functional metagenomics (FG), which are believed to be intrinsic to environmental bacteria, and not yet mobilized. The ResFinderFG and CsabaPal collections contain solely genes identified with this technique.
- Other ARGs: Genes from the remaining databases that do not clearly fall into the other two categories. These are likely to include a mix of both types; however, due to a lack of annotations regarding mobilization and discovery, they were excluded from the main study.

Database	Version	# ARGs	Contains ARGs	Contains similarity-predicted genes	Identified with functional metagenomics
ResFinder[1]23/10/2025 11:38:00	2023-01-20	3,131	Yes	N	No**
ResFinderFG[2]	2.0	3,897	Yes	N	Yes
CARD[7]	3.2.5	4,661	Yes	Yes	No**
MegaRes[8]	3.0.0	8,295	Yes	Yes	No**
AMRFinderPlus[9]	3.11/2022-12-19.1	6,465	Yes	Yes	No**
ARG-ANNOT[10]	V6_July2019	2,223	Yes	Yes	No**
CsabaPal[3]	November 2022	1,093	Yes	No	Yes
MetalResistance*	https://doi.org/10.5281/zenodo.8108201	578	No	No	No

Supplementary Table 5. ***The databases included in PanRes v1.0.1****, including their version number, how many reference sequences were included, whether they contain ARGs or not, and an indicator of whether the source contains genes identified through similarity predictions.*

** MetalResistance is an in-house collection that contains both genes from BacMet[11] v1.1 and a manual query.*

*** Since the ResFinderFG genes were identified by reviewing published studies, several genes are already included in the other databases.*

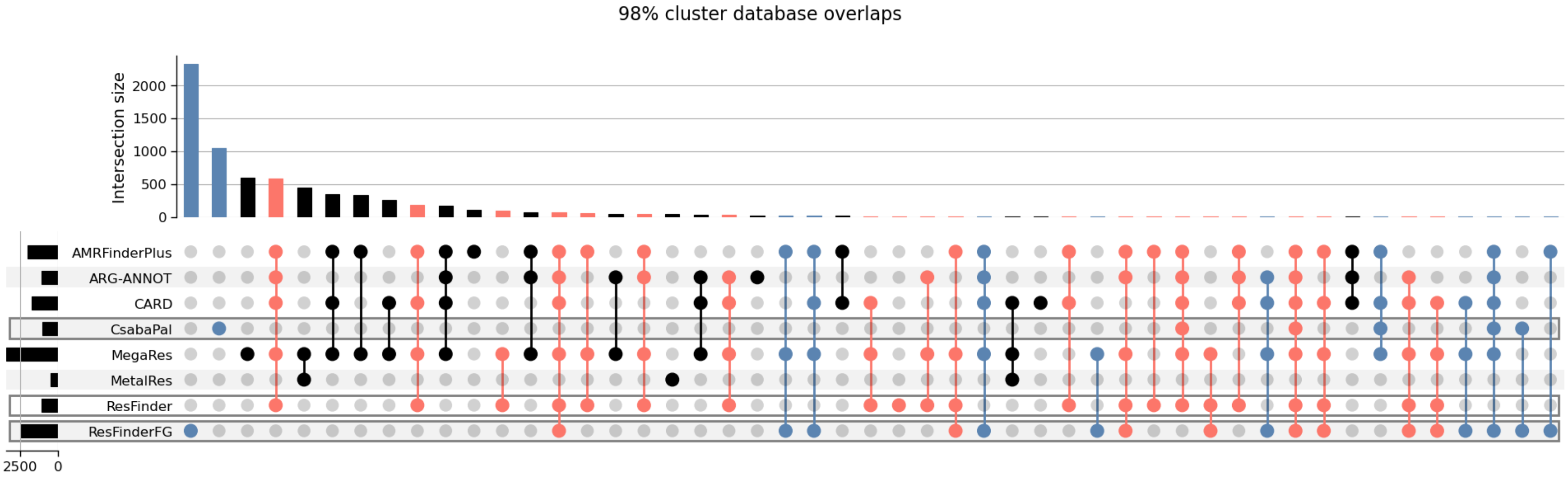

Supplementary Figure 13. ***Upset plot showing the overlap for all PanRes databases in their representative members for the 98% CD-HIT clustering****. Highlighted rows show the chosen databases for which we created groupings: ResFinder, ResFinderFG, and CsabaPal. The coloring follows the scheme used in the main paper to distinguish between Acquired (red) and FG (blue) ARGs.*

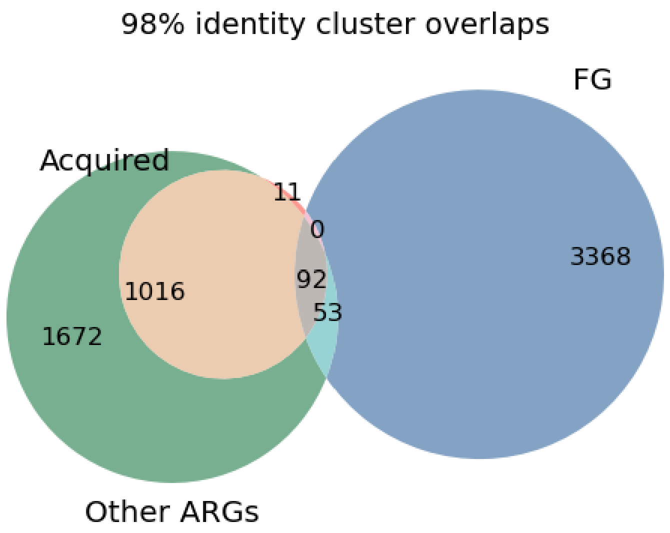

*Supplementary Figure 14. **Overlap in representative cluster sequences between the groupings** of Acquired (ResFinder), FG (ResFinderFG+CsabaPal), and the other ARGs not included in the main manuscript (CARD, MegaRes, AMRFinderPlus, ARGANNOT).*

Primary analyses

To determine whether our focus on acquired and FG ARGs introduced any bias into our study, we have compared the abundance, diversity, and variants of all three ARG categories.

Read abundances

To start with, we compared the number of sequences matched with at least one read fragment, the total number of fragments aligned, and the sample-wise summary statistics (average, range, and standard deviations) of fragment counts, as shown in Supplementary Table 6. Among the representative sequences clustered at 98% identity, we observed that the acquired ARGs had the highest proportion of sequences that were hit. In terms of sample-wise fragment counts, FG and Other ARGs had similar levels, while the acquired ARGs were lower in read fragments aligned.

	**Acquired**	**FG**	**Other ARGs**	**All ARGs**
**Representative 98% sequences hit (%)**	1,052 / 1,119 (94.01%)	3,108 / 3,421 (90.85%)	1,512 / 1,672 (90.43%)	5,645 / 6,212 (90.87%)
**Total fragments aligned to sequences (M: million)**	17.28 M	21.75 M	31.01 M	70.05 M
**Average of fragments aligned to sequences (M: million)**	0.015 M	0.018 M	0.027 M	0.061 M
**Range of fragments aligned (M: million)**	0.0002 M - 0.113 M	0.0006 M - 0.239 M	0.001 M - 0.209 M	0.003 M - 0.438 M
**Standard deviation of fragments aligned**	0.013 M	0.016 M	0.019 M	0.043 M

*Supplementary Table 6**: Summary of fragments aligned to the references** in the three different groupings: Acquired, FG, and Other ARGs. Abbreviations: M: Million.*

Next, we calculated the alpha- and beta-diversities of the fragment counts for the three different groupings (Supplementary Figure 15 and Supplementary Figure 16). Supplementary Figure 15 compares the aligned fragment of ARGs to the amount aligned to bacterial genera, the absolute richness, and the Shannon diversities. While roughly the same total amount of fragments was aligned, the FG and other ARGs exhibited higher richness and Shannon diversity than the acquired ARGs. Interestingly, the sample-wise diversity metrics of the other ARGs consistently fell between those of the FG and acquired ARGs, supporting our interpretation that this group likely represents a mixture of both mobilized and intrinsic resistance genes.

Continuing this, we saw that the country-wise abundances of the ARGs in Supplementary Figure 16a also indicated that the other ARGs are a group of mobilized and intrinsic resistance genes, although the abundances were almost the same in some countries, for example, in Georgia, Tanzania, and Vietnam. For the beta diversity visualized in the PCA biplots in Supplementary Figure 16, we saw that the beta diversity could be explained by 8.9% by world regions, which was slightly higher than the FG ARGs at 7.4% and lower than the acquired at 12% (Supplementary Table 7). Similarly, the other ARGs were more related to the bacteriome than the FG ARGs and less than the acquired ARGs.

	**Acquired ARGs**	**FG ARGs**	**Other ARGs**
**Beta diversity explained by regions (permanova)**	12% (*P*=0.001)	7.4% (*P*=0.001)	8.9 % (*P*=0.001)
**Relatedness to bacteriome (procrustes)**	0.88 (*P*=0.001)	0.69 (*P*=0.001)	0.79 (*P*=0.001)

Supplementary Table 7. ***Permanova and Procrustes tests on the beta-diversity of the resistomes*** *in relation to world regions and bacteriome.*

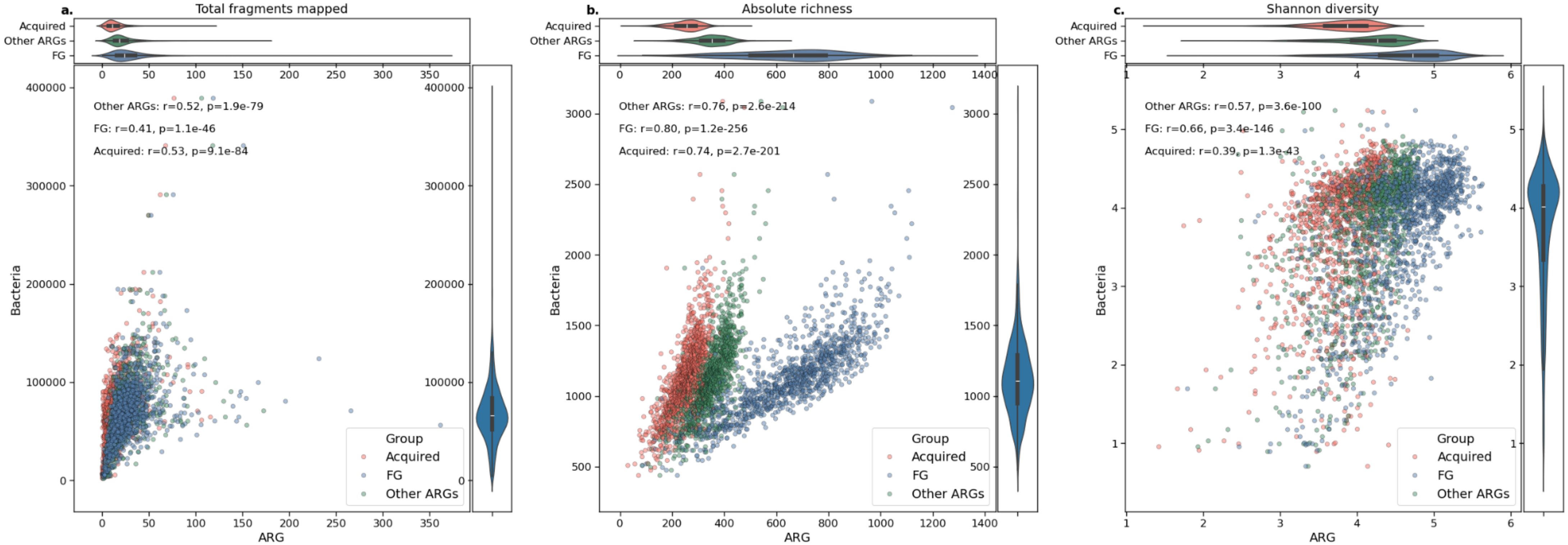

Supplementary Figure 15. ***Alpha-diversity indices of antimicrobial resistance genes (x-axis) and the bacterial genera (y-axis) found in the sewage samples****, stratified by ARG grouping.* ***a****. The number of read fragments aligned to either the Acquired ARGs, FG ARGs, or Other ARGs against the fragments aligned to the bacterial genera.* ***b****. Absolute Richness of the ARGs and genera.* ***c****. Shannon diversity indices for ARGs and bacterial genera.*

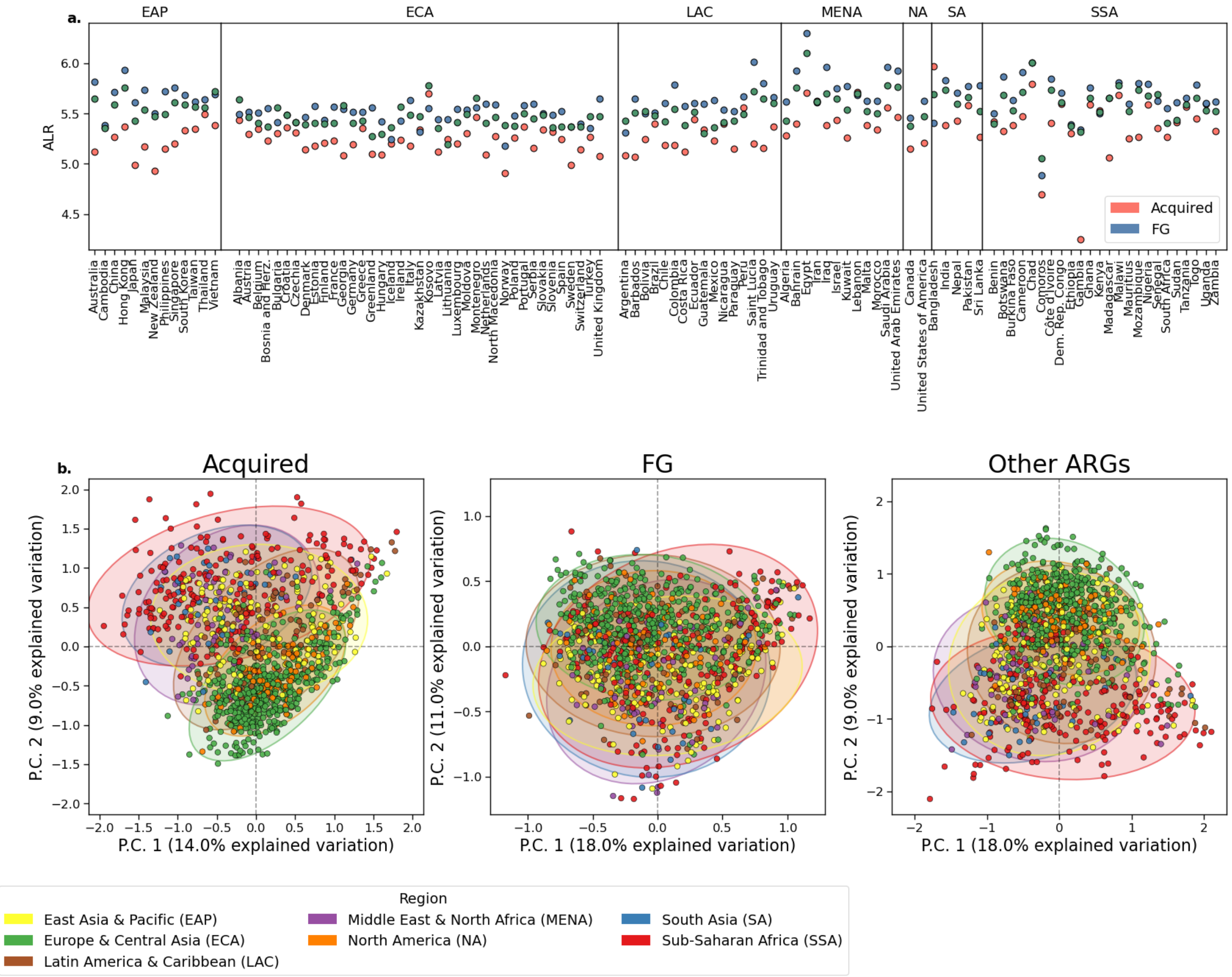

Supplementary Figure 16. ***Abundance and beta-diversity of ARGs across geographical regions. a****. The country-wise ALR abundances of acquired ARGs, FG ARGs, and Other ARGs.* ***b.*** *PCA biplots of resistance genes (98% homology grouping), in which the PCA loadings were calculated from CLR values, with the acquired ARGs left, FG ARGs in the middle, and the other ARGs on the right. Each marker represents a sewage sample and is colored by the world region.*

Distance-decay effects

Building on the observed diversity patterns, we next investigated whether geographic distance between sampling sites contributes to differences in other ARGs beyond those observed in the main manuscript (Supplementary Figure 17 and Supplementary Figure 18).

In the distance-decay models based on abundance data (Supplementary Figure 17), the slopes for the other ARGs closely resembled those of the FG ARGs (Supplementary Table 8). However, at the inter-regional scale, the slope for the other ARGs became slightly positive ($slope_{between\ region}$=0.012), more similar to the slope observed for the bacterial abundance ($slope_{between\ region}$=0.015). This suggests that, like the FG ARGs, the other ARGs may be more environmentally embedded and less constrained by geographic distance. Mantel tests further supported this, showing that the other ARGs were slightly less correlated to the bacteriome (ρ=0.73; P=0.001) than FG ARGs (ρ=0.76; P=0.001), but more so than the acquired ARGs (ρ=0.7; P=0.001).

When analyzing the variants of the other ARGs discovered in the metagenomic assemblies, we did not observe a strong clustering of cities sharing the same variants in the UMAP plot (Supplementary Figure 18c). The distance-decay slopes for the other ARG were also weak across all spatial scales, indicating limited effects of geographic distance on the distribution of variants (Supplementary Figure 18, Supplementary Table 8). Moreover, there were no strongly significant differences in the distance-decay slopes (Supplementary Table 9), further suggesting that the other ARGs are not facing strong dispersal limitations.

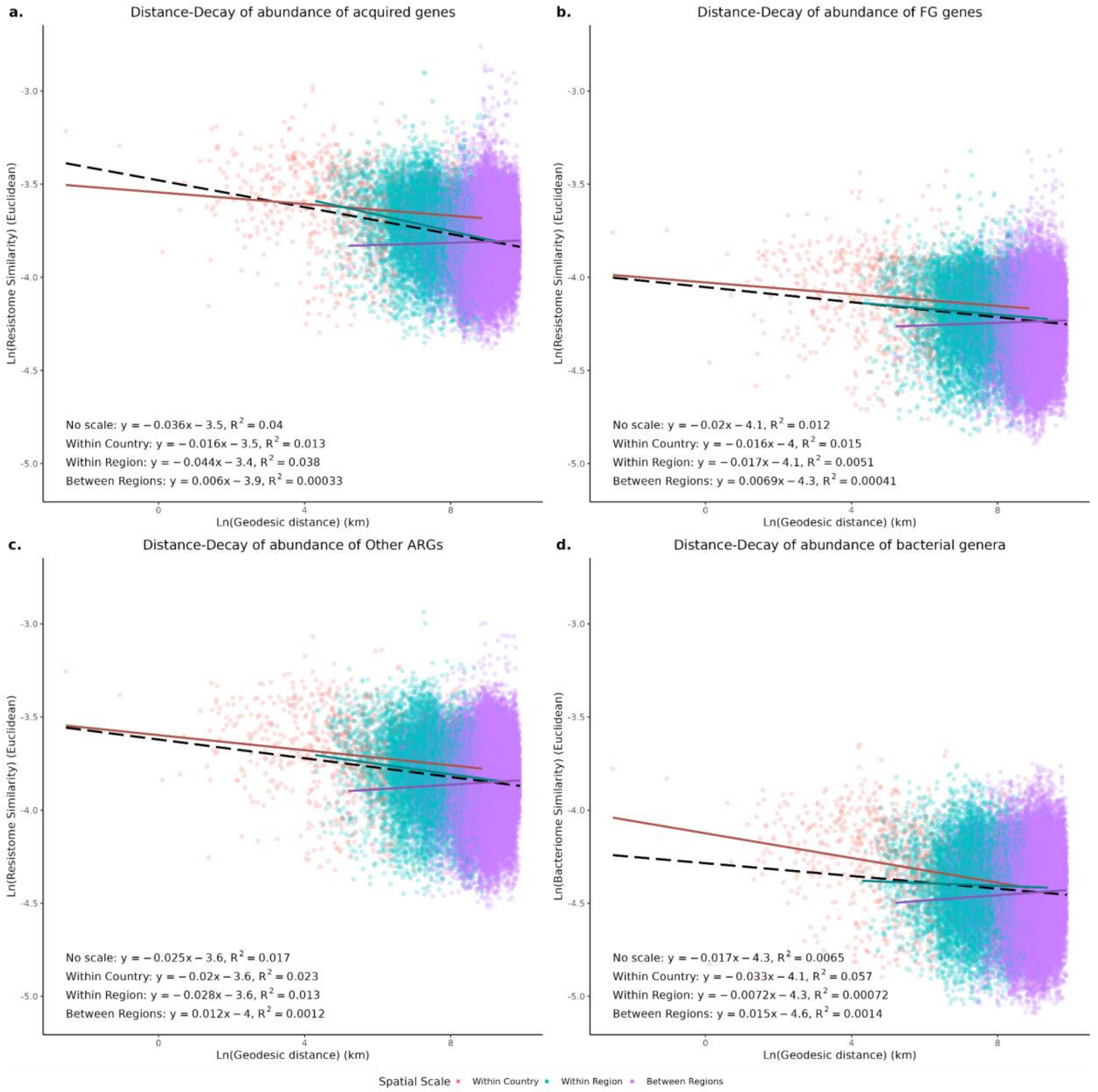

Supplementary Figure 17. ***Distance-Decay relationships for the bacteriome and resistome city communities*** *(n=60,030 pairwise comparisons) for the abundance of* ***a****. Acquired ARGs,* ***b.*** *FG ARGs,* ***c.*** *Other ARGs, and* ***d.*** *bacterial genera. The x-axis shows the pairwise city distances in kilometers (km), and the y-axis shows similarities. The dashed line represents the fit across all spatial scales, where the solid lines denote regressions fitted individually by different city-wise comparisons: national (red), cities within the same region (blue), and inter-regional (between regions, purple). Model parameters and adjusted* R^2 *values are listed for each model in its corresponding plot.*

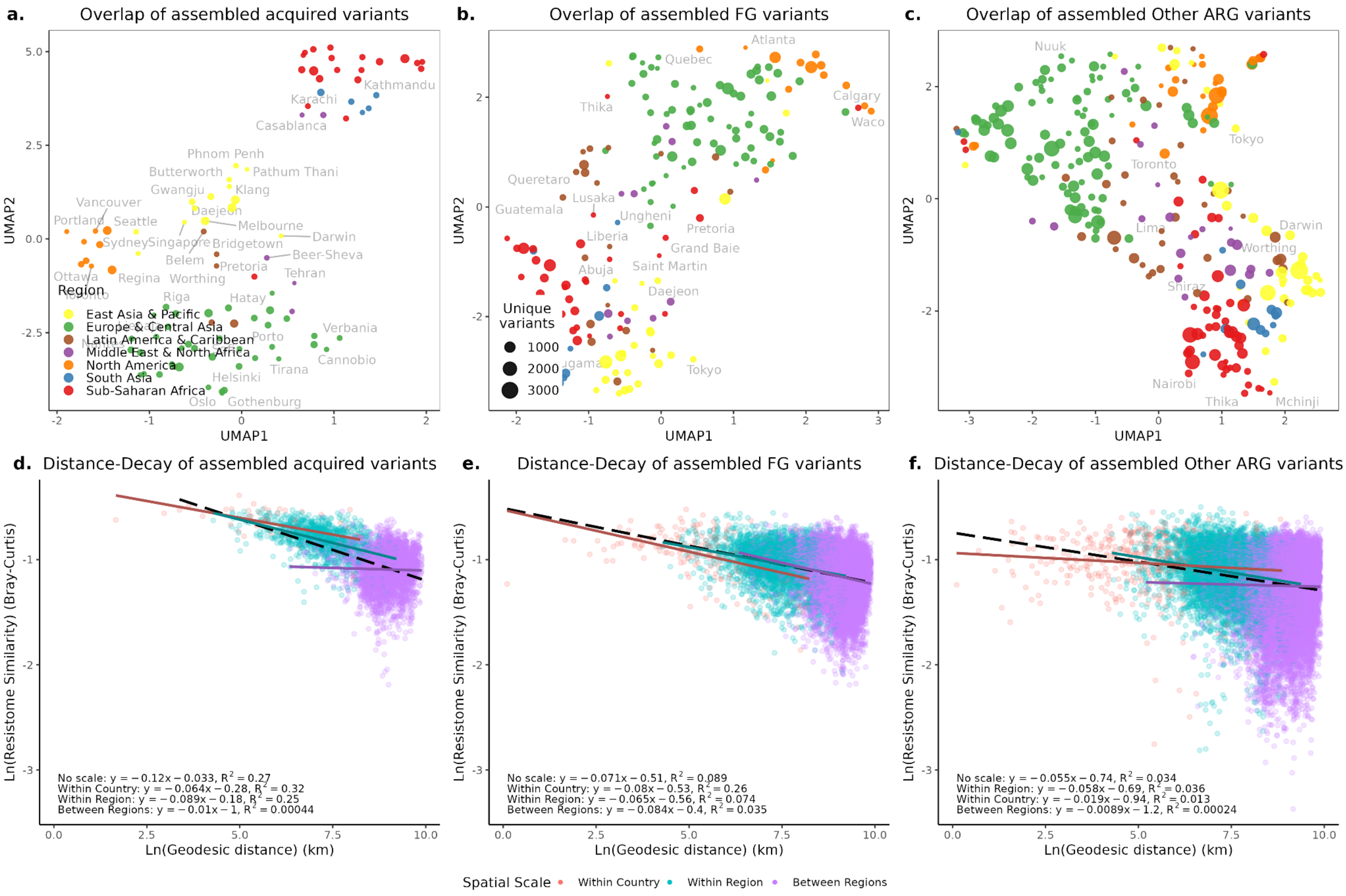
a.
Overlap of assembled acquired variants
b.
Overlap of assembled FG variants
c.
Overlap of assembled Other ARG variants
UMAP1
UMAP2
Region
East Asia & Pacific
Europe & Central Asia
Latin America & Caribbean
Middle East & North Africa
North America
South Asia
Sub-Saharan Africa
Unique variants
1000
2000
3000
d.
Distance-Decay of assembled acquired variants
e.
Distance-Decay of assembled FG variants
f.
Distance-Decay of assembled Other ARG variants
Ln(Resistome Similarity) (Bray-Curtis)
Ln(Geodesic distance) (km)
No scale: y = −0.12x − 0.033, R2 = 0.27
Within Country: y = −0.064x − 0.28, R2 = 0.32
Within Region: y = −0.089x − 0.18, R2 = 0.25
Between Regions: y = −0.01x − 1, R2 = 0.00044
No scale: y = −0.071x − 0.51, R2 = 0.089
Within Country: y = −0.08x − 0.53, R2 = 0.26
Within Region: y = −0.065x − 0.56, R2 = 0.074
Between Regions: y = −0.084x − 0.4, R2 = 0.035
No scale: y = −0.055x − 0.74, R2 = 0.034
Within Region: y = −0.058x − 0.69, R2 = 0.036
Within Country: y = −0.019x − 0.94, R2 = 0.013
Between Regions: y = −0.0089x − 1.2, R2 = 0.00024
Spatial Scale
Within Country
Within Region
Between Regions

Supplementary Figure 18. ***UMAP and Distance-Decay Analysis of resistomes**. **a.-c.*** *UMAP clustering of shared variants among the cities for a. acquired ARGs, b. FG ARGs and c. Other ARGs. Only cities with more than 100 non-singleton alleles were retained, and Hellinger transformed and clustered with the UMAP algorithm. Each marker represents a city, colored by region and sized according to the number of unique variants in that city. City labels were optimized using the ggrepel package to minimize overlap.* ***d.-f.*** *Distance-decay relationships for assembled city resistomes across different spatial scales for* ***d****. acquired (n=4,656 pairwise comparisons), the FG variants (n=16,471 pairwise comparisons), and* ***f.*** *Other ARGs (n=39,903 pairwise comparisons). The x-axis shows the pairwise city distances in kilometers (km), and the y-axis shows resistome similarities. The dashed line represents the fit across all spatial scales, where the solid lines denote regressions fitted individually for three spatial scales: cities within the same country (red), cities within the same region (blue), and cities that are in different regions (between regions, purple). Model parameters and the adjusted R^2 values are written for each model in its corresponding plot.*

Model		Abundance				Assembly variants		
		Acquired	FG	Other ARGs	Genera	Acquired	FG	Other ARGs
Scale Independent	R^2	0.04	0.012	0.017	0.0065	0.27	0.089	0.034
	Slope	-0.036*** (*P*<<0)	-0.02*** (*P*=3.8e-155)	-0.025*** (*P*=1.5e-219)	-0.017*** (*P*=9.5e-88)	-0.12*** (*P*=3.01e-322)	-0.071*** (*P*=0)	-0.055*** (*P*=1.6e-299)
	Mantels *r*	0.22** (*P*=0.001)	0.12** (*P*=0.001)	0.14** (*P*=0.001)	0.091** (*P*=0.001)	0.6** (*P*=0.001)	0.32** (*P*=0.001)	0.22** (*P*=0.001)
Within Country	R^2	0.013	0.015	0.023	0.057	0.32	0.26	0.036
	Slope	-0.016** (*P*=0.0003)	-0.016** (*P*=0.0002)	-0.02*** (*P*=3.2e-06)	-0.033*** (*P*=2.2e-13)	-0.064*** (*P*=6e-06)	-0.08*** (*P*=5.6e-15)	0.058*** (*P*=3.40e-63)
	Mantels *r*	0.14** (*P*=0.001)	0.15** (*P*=0.001)	0.18** (*P*=0.001)	0.26** (*P*=0.001)	0.57** (*P*=0.001)	0.49** (*P*=0.001)	0.2** (*P*=0.001)
Within Region	R^2	0.038	0.0051	0.013	0.00072	0.25	0.074	0.013
	Slope	-0.044*** (*P*=2.5e-100)	-0.017*** (*P*=4.2e-15)	-0.028*** (*P*=2.1e-35)	-0.0072* (*P*=0.0021)	-0.089*** (*P*=4e-66)	-0.089*** (*P*=4e-66)	-0.019* (*P*=4.9e-3)
	Mantels *r*	0.2** (*P*=0.001)	0.079** (*P*=0.001)	0.12** (*P*=0.001)	0.034** (*P*=0.001)	0.51** (*P*=0.001)	0.27** (*P*=0.001)	0.0.12** (*P*=0.001)
Between Regions	R^2	0.00033	0.00041	0.001	0.0014	0.00044	0.035	0.00024
	Slope	0.006*** (*P*=4.2e-05)	0.0069*** (*P*=6.7e-06)	0.012*** (*P*=3.0e-14)	0.015*** (*P*=2e-16)	-0.01 (*P*=0.11)	-0.084*** (*P*=1.7e-102)	-0.0089* (*P*=3.5e-3)
	Mantels *r*	-0.02 (*P*=1)	-0.025 (*P*=1)	-0.042 (*P*=1)	-0.044 (*P*=1)	0.013 (*P*=0.22)	0.19** (*P*=0.001)	0.04** (*P*=0.001)

*Supplementary Table 8. **Results of the linear regression models and statistical tests to investigate the association between resistome similarity and sampling distances**. Mantel tests were performed between dissimilarity and distance matrices, and the distance-decay models used ln-transformed similarity matrices (1-dissimilarity). Dissimilarities were measured either by Aitchison distances for the Abundance models or Bray-Curtis distances for the assembly variant models. Asterisks: * P ≤ 0.01, ** P ≤ 0.001, and *** P ≤ 0.0001.*

Data input	Group	Comparison	Estimated Difference	Std. Error	t-ratio	*P*-value
Abundance	Acquired	Within Country - Within Region	0.0282	0.0038	7.4643	2.71e-13***
		Within country - Between Regions	-0.0216	0.0036	-6.0431	4.53e-09***
		Within region - Between Regions	-0.04978	0.0024	-20.4194	0.0e+00***
	FG	Within Country - Within Region	0.0039	0.0040	0.2520	9.66e-01
		Within country - Between Regions	-0.0227	0.0038	-6.0289	4.95e-09***
		Within region - Between Regions	-0.0237	0.0026	-9.2234	3.34e-14***
	Other ARGs	Within Country - Within Region	0.00750	0.0042	1.8078	1.67e-01
		Within country - Between Regions	-0.0325	0.0039	-8.2713	3.38e-14***
		Within region - Between Regions	-0.040	0.0027	-14.9193	0.0e+00**
	Bacteria	Within Country - Within Region	-0.0259	0.0045	-5.7449	2.76e-08***
		Within country - Between Regions	-0.0476	0.0043	-11.1680	8.66e-15***
		Within region - Between Regions	-0.0217	0.0029	-7.4605	2.78e-13***
Assembly variants	Acquired	Within Country - Within Region	0.0247	0.0178	1.3906	3.46e-01
		Within country - Between Regions	-0.0538	0.0175	-3.0711	6.08e-03*
		Within region - Between Regions	-0.0785	0.0089	-8.8021	3.90e-08***
	FG	Within Country - Within Region	-0.0146	0.0107	-1.3631	3.60e-01
		Within country - Between Regions	0.00390	0.0103	0.3777	9.24e-01
		Within region - Between Regions	0.0185	0.0060	3.0965	5.57e-03*
	Other ARGs	Within Country - Within Region	0.00395	0.0078	5.0274	1.48e-06***
		Within country - Between Regions	-0.0100	0.0074	-1.3544	3.65e-01
		Within region - Between Regions	-0.0495	0.0049	-10.1203	3.02e-14***

Supplementary Table 9. ***Pairwise differences between the slopes of the linear regression models for distance-decays.*** *The estimated difference represents the contrast between the slopes of two spatial scales, along with associated standard errors, test statistics (t-ratios), and P-values indicating the significance of each comparison. Asterisks: * P ≤ 0.01, ** P ≤ 0.001, and *** P ≤ 0.0001.*

Conclusion

Here, we present a brief overview of the content of the entire PanRes database and how we have distinguished between the different ARGs in our paper, "*Geographics and bacterial networks shape the global urban sewage resistome*." We repeated the primary analyses on compositional data and the distance-decay analyses to demonstrate how the main conclusions of the paper have been influenced by our decision to focus only on specific groupings.

PanRes encompasses more than 14,000 genes that confer resistance to antimicrobials, biocides, and metals, representing a diverse array of genetic sequences. However, by combining so many different types of collections into one, we introduced a large uncertainty regarding gene annotations and biological origins. To study the latent reservoir of antimicrobial resistance in sewage resistomes, we focused on ARGS with well-curated discovery methodologies and phenotypic validation (Supplementary Table 5). Specifically, we selected:

- ResFinder[1] for the mobilized, or acquired, ARGs and
- ResFinderFG[2] and the Daruka et al. (2025)[3] for ARGs identified with functional metagenomics (FG), representing the latent reservoir.

Supplementary Figures 13 and 14 showed that the FG ARGs had minimal overlap with the remaining PanRes ARGs, while the acquired ARGs appeared to be a subset of the broader collection ARGs.

Supplementary Table 6 summarizes the read alignment counts across the three different groupings, where we observed that the other ARGs were the group with the most fragments aligned overall and per sample. However, the FG ARGs were still more abundant than the acquired ARGs. Similarly, we could see from the alpha diversity metrics that the FG ARGs showed higher richness and diversity than the acquired ARGs, where the other ARGs fell between the two (Supplementary Figure 15). The abundance and beta diversity metrics showed similar behavior with some regional separation (Supplementary Figure 16), which, taken together, show that the other ARGs may be more diverse, but their biological relevance for our study remains unclear.

The analyses of distance-decay patterns on both the abundance and the dispersal of variants detected in metagenomic assemblies showed that the other ARGs did not fundamentally alter our conclusions that the FG ARGs behave differently than the acquired ARGs in terms of geographic spread (Supplementary Figure 17, Supplementary Figure 18, Supplementary Tables 8-9).

In conclusion, our analyses of the remaining other ARGs demonstrated that this group of genes consistently exhibited mixed characteristics across all of our analyses, suggesting that they represent a mixture of true mobilized, thus acquired, and non-mobilized ARGs. Furthermore, these genes lack a clear definition in contrast to our chosen subset, namely that they have not been laboratory-verified to cause phenotypic resistance. Therefore, we opted to exclude these genes from the main manuscript.

References

1. Bortolaia, V. *et al.* ResFinder 4.0 for predictions of phenotypes from genotypes. *Journal of Antimicrobial Chemotherapy* **75**, 3491–3500 (2020).
2. Gschwind, R. *et al.* ResFinderFG v2.0: a database of antibiotic resistance genes obtained by functional metagenomics. *Nucleic Acids Research* gkad384 (2023) doi:10.1093/nar/gkad384.
3. Daruka, L. *et al.* ESKAPE pathogens rapidly develop resistance against antibiotics in development in vitro. *Nat Microbiol* 1–19 (2025) doi:10.1038/s41564-024-01891-8.
4. Ruscheweyh, H. J. *et al.* Cultivation-independent genomes greatly expand taxonomic-profiling capabilities of mOTUs across various environments. *Microbiome* **10**, 1–12 (2022).
5. Thorn, A. V., Aarestrup, F. M. & Munk, P. Flankophile: a bioinformatic pipeline for prokaryotic genomic synteny analysis. *Microbiology Spectrum* **12**, e02413-23 (2023).
6. Martiny, H.-M. *et al.* ARGprofiler—a pipeline for large-scale analysis of antimicrobial resistance genes and their flanking regions in metagenomic datasets. *Bioinformatics* **40**, btae086 (2024).
7. Alcock, B. P. *et al.* CARD 2023: expanded curation, support for machine learning, and resistome prediction at the Comprehensive Antibiotic Resistance Database. *Nucleic Acids Res* **51**, D690–D699 (2023).
8. Bonin, N. *et al.* MEGARes and AMR++, v3.0: an updated comprehensive database of antimicrobial resistance determinants and an improved software pipeline for classification using high-throughput sequencing. *Nucleic Acids Research* **51**, D744–D752 (2023).
9. Feldgarden, M. *et al.* AMRFinderPlus and the Reference Gene Catalog facilitate examination of the genomic links among antimicrobial resistance, stress response, and virulence. *Scientific Reports* **11**, (2021).

10. Gupta, S. K. *et al.* ARG-ANNOT, a New Bioinformatic Tool To Discover Antibiotic Resistance Genes in Bacterial Genomes. *Antimicrobial Agents and Chemotherapy* **58**, 212–220 (2014).

11. Pal, C., Bengtsson-Palme, J., Rensing, C., Kristiansson, E. & Larsson, D. G. J. BacMet: antibacterial biocide and metal resistance genes database. *Nucleic Acids Research* **42**, D737–D743 (2014).
