# Supplementary material for: Geographics and bacterial networks differently shape the acquired and latent global sewage resistomes
Source: Nat Commun. 2025 Nov 21;16:10278. doi: 10.1038/s41467-025-66070-7 (PMC12639157; doi:10.1038/s41467-025-66070-7)
Supplement: Supplementary file 2 — Reporting Summary [file 41467_2025_66070_MOESM2_ESM.pdf]

nature portfolio

Corresponding author(s): Hannah-Marie Martiny, Patrick Munk

Last updated by author(s): Feb 13, 2025

Reporting Summary

Nature Portfolio wishes to improve the reproducibility of the work that we publish. This form provides structure for consistency and transparency in reporting. For further information on Nature Portfolio policies, see our Editorial Policies and the Editorial Policy Checklist.

Statistics

For all statistical analyses, confirm that the following items are present in the figure legend, table legend, main text, or Methods section.

n/a	Confirmed	
☐	☒	The exact sample size (*n*) for each experimental group/condition, given as a discrete number and unit of measurement
☐	☒	A statement on whether measurements were taken from distinct samples or whether the same sample was measured repeatedly
☐	☒	The statistical test(s) used AND whether they are one- or two-sided<br>*Only common tests should be described solely by name; describe more complex techniques in the Methods section.*
☐	☒	A description of all covariates tested
☐	☒	A description of any assumptions or corrections, such as tests of normality and adjustment for multiple comparisons
☐	☒	A full description of the statistical parameters including central tendency (e.g. means) or other basic estimates (e.g. regression coefficient) AND variation (e.g. standard deviation) or associated estimates of uncertainty (e.g. confidence intervals)
☐	☒	For null hypothesis testing, the test statistic (e.g. *F*, *t*, *r*) with confidence intervals, effect sizes, degrees of freedom and *P* value noted<br>*Give P values as exact values whenever suitable.*
☒	☐	For Bayesian analysis, information on the choice of priors and Markov chain Monte Carlo settings
☒	☐	For hierarchical and complex designs, identification of the appropriate level for tests and full reporting of outcomes
☐	☒	Estimates of effect sizes (e.g. Cohen's *d*, Pearson's *r*), indicating how they were calculated

Our web collection on statistics for biologists contains articles on many of the points above.

Software and code

Policy information about availability of computer code

Data collection	No software was used to collect data

Data analysis	The ARGprofiler pipeline was used to analyze sequencing reads: https://github.com/genomicepidemiology/ARGprofiler<br>- includes: fastp 0.23.2, KMA 1.4.12a, spades 3.15.5<br>USEARCH v.11.0.66<br>R 4.3.2<br>Python 3.12<br>Matplotlib 3.8.2<br>Seaborn 0.13.2<br>Geopandas 0.14.3<br>pycodamath 1.0 https://bitbucket.org/genomicepidemiology/pycodamath<br>pandas 2.3.2<br>Vegan 2.6<br>Flankophile https://bitbucket.org/genomicepidemiology/flankophile<br>umap 0.2.10.0<br>proxyC 0.4.1<br>mgnet https://github.com/Fuschi/mgnet<br>All code has been deposited on GitHub: https://github.com/genomicepidemiology/gs3_acquired_vs_FG.

For manuscripts utilizing custom algorithms or software that are central to the research but not yet described in published literature, software must be made available to editors and reviewers. We strongly encourage code deposition in a community repository (e.g. GitHub). See the Nature Portfolio guidelines for submitting code & software for further information.

Data

Policy information about availability of data

All manuscripts must include a data availability statement. This statement should provide the following information, where applicable:

- Accession codes, unique identifiers, or web links for publicly available datasets
- A description of any restrictions on data availability
- For clinical datasets or third party data, please ensure that the statement adheres to our policy

The sequenced reads and metagenomic assemblies have been deposited at the European Nucleotide Archive for the different rounds of sampling, which are available under project accession numbers: PRJEB40798, PRJEB40816, PRJEB40815, PRJEB27621, and PRJEB84064. Additional data, such as KMA mapstat files, count matrices, and flankophile output, have been deposited on Zenodo at 10.5281/zenodo.14652833.

The reference databases are available at: mOTUs https://zenodo.org/records/7778108, PanRes https://zenodo.org/records/13885013 and UCHGG at https://ftp.ebi.ac.uk/pub/databases/metagenomics/mgnify_genomes/

Research involving human participants, their data, or biological material

Policy information about studies with human participants or human data. See also policy information about sex, gender (identity/presentation), and sexual orientation and race, ethnicity and racism.

Reporting on sex and gender	Not applicable.
Reporting on race, ethnicity, or other socially relevant groupings	Not applicable.
Population characteristics	Not applicable.
Recruitment	Not applicable.
Ethics oversight	Not applicable.

Note that full information on the approval of the study protocol must also be provided in the manuscript.

Field-specific reporting

Please select the one below that is the best fit for your research. If you are not sure, read the appropriate sections before making your selection.

☐ Life sciences ☐ Behavioural & social sciences ☒ Ecological, evolutionary & environmental sciences

For a reference copy of the document with all sections, see nature.com/documents/nr-reporting-summary-flat.pdf

Ecological, evolutionary & environmental sciences study design

All studies must disclose on these points even when the disclosure is negative.

Study description	The study is an observational study of genomic material extracted from untreated sewage samples, with a specific focus on antimicrobial resistance genes. Participants were instructed as in the pilot study (Hendriksen et al., 2019) and the second study (Munk et al., 2022) to collect untreated sewage in bottles and ship the samples back to Denmark for analysis with pre-paid shipping.

Research sample	Each research sample was an untreated sewage samples collected in one of the 351 cities in 111 countries. The DNA extracted from each sample reflects the microbiome with all genes present at that site at the time of sampling and the human population using the sewage system. The samples are meant to cover global difference in human-impacted urban sewage environments, but also contain an environmental contribution. We've previously shown that untreated sewage is a cost-effective and ethical way of surveying AMR globally (Hendriksen et al., 2019 and Munk et al., 2022).
Sampling strategy	As in the two studies, we advertised the new round of sampling widely and invited everyone in and out of our network to participate samples to the study. No exact sample size calculations were performed prior to the study, however, our aim was to cover as much as the world as possible. That means that the samples can be considered as convenience samples and we could not know how big of an global interest to contribute samples would be.<br><br>The same sampling protocol described in Hendriksen et al. (2019) was followed in this study: each participant was instructed to collect ~1L of untreated community sewage from the sewage plant befoe any potential treatment. Either 1L over a 24H sampling or an approach of 3 x 300 mL samples at least 5 minutes aparts.
Data collection	Each collaborating partner was instructed in how to take local waste water samples, freeze the samples and ship them back to Denmark at DTU's expense. The sampling bottles and other supplies were shipped to the partners. The individual partners at each discrete sampling site were responsible for taking pictures, filling out metadata and submitting it to DTU through Survey Monkey, as well as shipping the frozen samples to DTU.
Timing and spatial scale	Following the same sampling schedule outlined in Munk et. al (2022), the bi-annual sampling campaign (Winter, Summer) were done to avoid seasonal biases. All the new samples were collected between 2020 and 2022. The spatial scope of the sampling campaign was meant to expand our previous efforts to survey AMR at a large and broader global scale. The exact samples were convenience samples and the scope could not be pre-determined, but we aimed to include all countries in the world to cover the largest amount of the globe.
Data exclusions	No specific criteria for excluding data were not set prior to the study. We used the same samples as in the two previous studies and applied the same exclusions on the new samples. Samples with no information on sampling city and GPS coordinates was not used for the distance-decay analyses.
Reproducibility	The study design have been validated already in the pilot study by Hendriksen et al. (2019), hence why no attempts at classical replication were carried out in the second study by Munk et al. (2022) and in this study. In the pilot study included a reproducibility analysis on a number of samples taken one day apart at the same site. Technical replicates in the form of re-sequencing of the same library multiple times was also done.<br><br>Since this study reuses the samples included in the two previous studies (Hendriksen et al. 2019, Munk et al. 2022) and brings in together new ones, some of our results can be seen as reproduced; especially the confirmation that the ResFinder genes have the same regional resistome cluster patterns. We have thus managed to replicate the findings of the two previous studies.
Randomization	As there was no treatment groups used, no randomization was applied. Samples were organized based on the World Bank regions for data analyses and visualizations.
Blinding	No attempts of blinding individual persons were carried out as this is an observational study. The methods and software used to analyze genomic sequences were not presented with the associated metadata, thus were technically "blinded".

Did the study involve field work? ☐ Yes ☒ No

Reporting for specific materials, systems and methods

We require information from authors about some types of materials, experimental systems and methods used in many studies. Here, indicate whether each material, system or method listed is relevant to your study. If you are not sure if a list item applies to your research, read the appropriate section before selecting a response.

Materials & experimental systems

n/a	Involved in the study
☒	☐ Antibodies
☒	☐ Eukaryotic cell lines
☒	☐ Palaeontology and archaeology
☒	☐ Animals and other organisms
☒	☐ Clinical data
☒	☐ Dual use research of concern
☒	☐ Plants

Methods

n/a	Involved in the study
☒	☐ ChIP-seq
☒	☐ Flow cytometry
☒	☐ MRI-based neuroimaging

Plants

Seed stocks	*Report on the source of all seed stocks or other plant material used. If applicable, state the seed stock centre and catalogue number. If plant specimens were collected from the field, describe the collection location, date and sampling procedures.*
Novel plant genotypes	*Describe the methods by which all novel plant genotypes were produced. This includes those generated by transgenic approaches, gene editing, chemical/radiation-based mutagenesis and hybridization. For transgenic lines, describe the transformation method, the number of independent lines analyzed and the generation upon which experiments were performed. For gene-edited lines, describe the editor used, the endogenous sequence targeted for editing, the targeting guide RNA sequence (if applicable) and how the editor was applied.*
Authentication	*Describe any authentication procedures for each seed stock used or novel genotype generated. Describe any experiments used to assess the effect of a mutation and, where applicable, how potential secondary effects (e.g. second site T-DNA insertions, mosiacism, off-target gene editing) were examined.*
