# Supplementary material for: Geographics and bacterial networks differently shape the acquired and latent global sewage resistomes
Source: Nat Commun. 2025 Nov 21;16:10278. doi: 10.1038/s41467-025-66070-7 (PMC12639157; doi:10.1038/s41467-025-66070-7)
Supplement: Supplementary file 3 — Description of Additional Supplementary Files [file 41467_2025_66070_MOESM3_ESM.pdf]

File Name: GS3_Supplementary_Data1.xlsx
Description: Metadata for samples and associated ENA accession numbers.

File Name: GS3_Supplementary_Data2.xlsx
Description: Metadata for the ARGs studied in the main manuscript.

File Name: GS3_Supplementary_Data3.xlsx
Description: Data for nodes in the network analysis (Figure 4, Supplementary Figures 10-12).

File Name: GS3_Supplementary_Data4.xlsx
Description: Data for the links in the network analysis (Figure 4, Supplementary Figures 10-12).

File Name: GS3_Supplementary_Data5.xlsx
Description: Human community composition in the network analysis (Figure 4, Supplementary Figures 10-12).

File Name: GS3_Supplementary_Data6_class_pca_abns.pdf
Description: PCA abundance analyses for resistance classes, showing that the class abundances differ across the world regions.
