# Supplementary material for: Geographics and bacterial networks differently shape the acquired and latent global sewage resistomes
Source: Nat Commun. 2025 Nov 21;16:10278. doi: 10.1038/s41467-025-66070-7 (PMC12639157; doi:10.1038/s41467-025-66070-7)
Supplement: Supplementary file 9 — Supplementary Data 6 [file 41467_2025_66070_MOESM9_ESM.pdf]

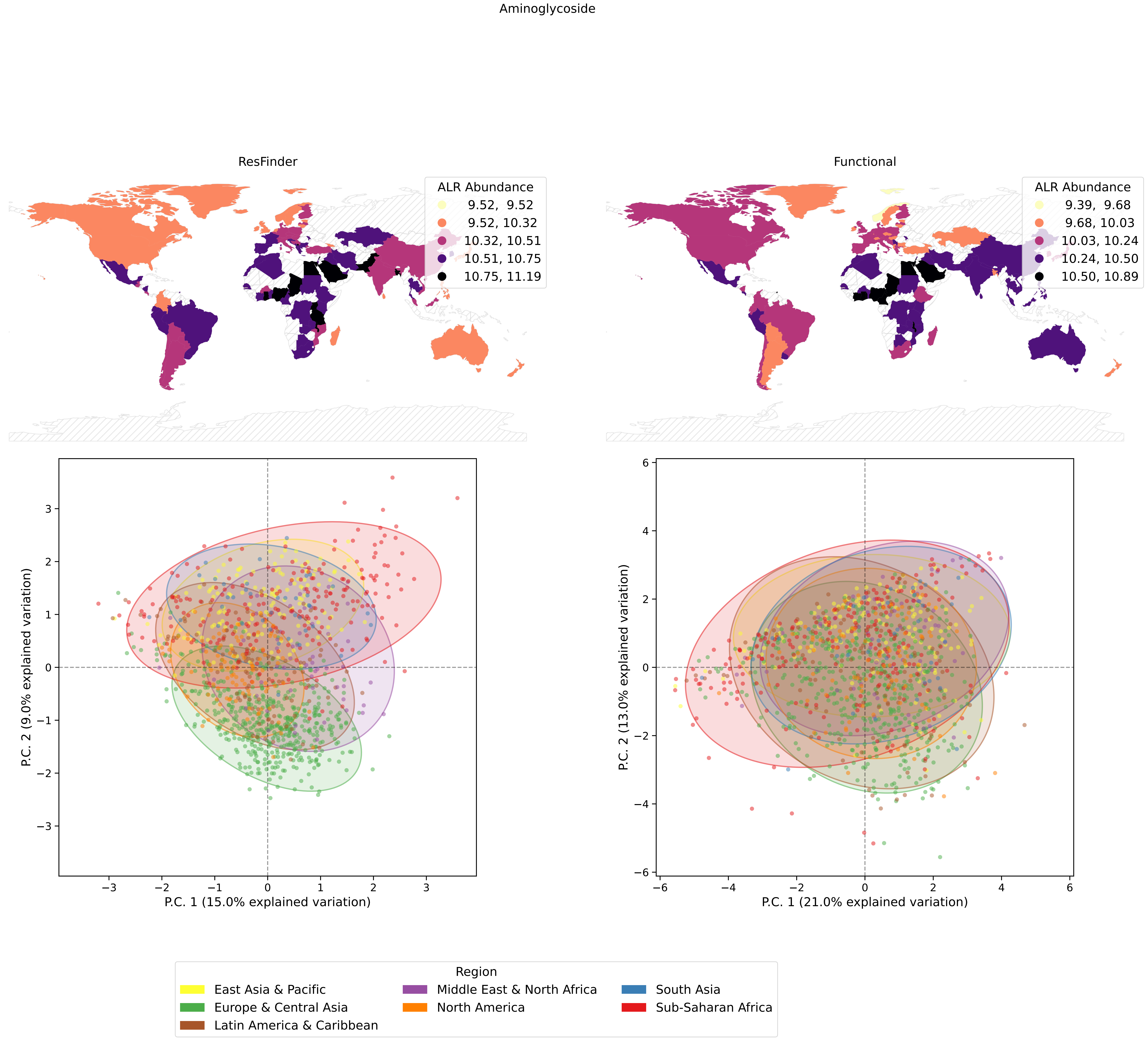

Aminoglycoside
ResFinder
Functional
ALR Abundance
9.52, 9.52
9.52, 10.32
10.32, 10.51
10.51, 10.75
10.75, 11.19
ALR Abundance
9.39, 9.68
9.68, 10.03
10.03, 10.24
10.24, 10.50
10.50, 10.89
P.C. 1 (15.0% explained variation)
P.C. 2 (9.0% explained variation)
P.C. 1 (21.0% explained variation)
P.C. 2 (13.0% explained variation)
Region
East Asia & Pacific
Europe & Central Asia
Latin America & Caribbean
Middle East & North Africa
North America
South Asia
Sub-Saharan Africa

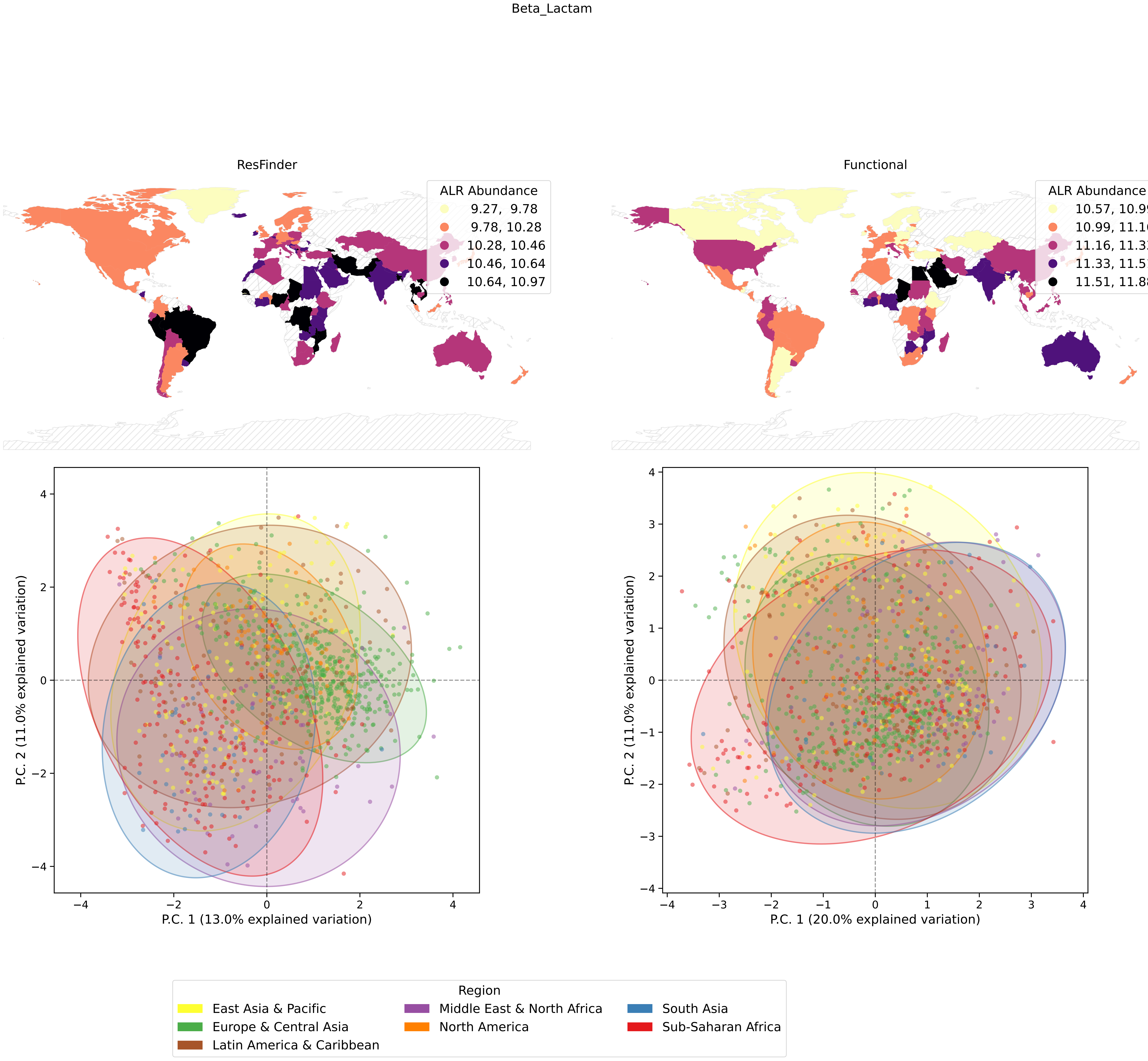
Beta_Lactam
ResFinder
ALR Abundance
9.27, 9.78
9.78, 10.28
10.28, 10.46
10.46, 10.64
10.64, 10.97
Functional
ALR Abundance
10.57, 10.99
10.99, 11.16
11.16, 11.33
11.33, 11.51
11.51, 11.88
P.C. 1 (13.0% explained variation)
P.C. 2 (11.0% explained variation)
P.C. 1 (20.0% explained variation)
P.C. 2 (11.0% explained variation)
Region
East Asia & Pacific
Europe & Central Asia
Latin America & Caribbean
Middle East & North Africa
North America
South Asia
Sub-Saharan Africa

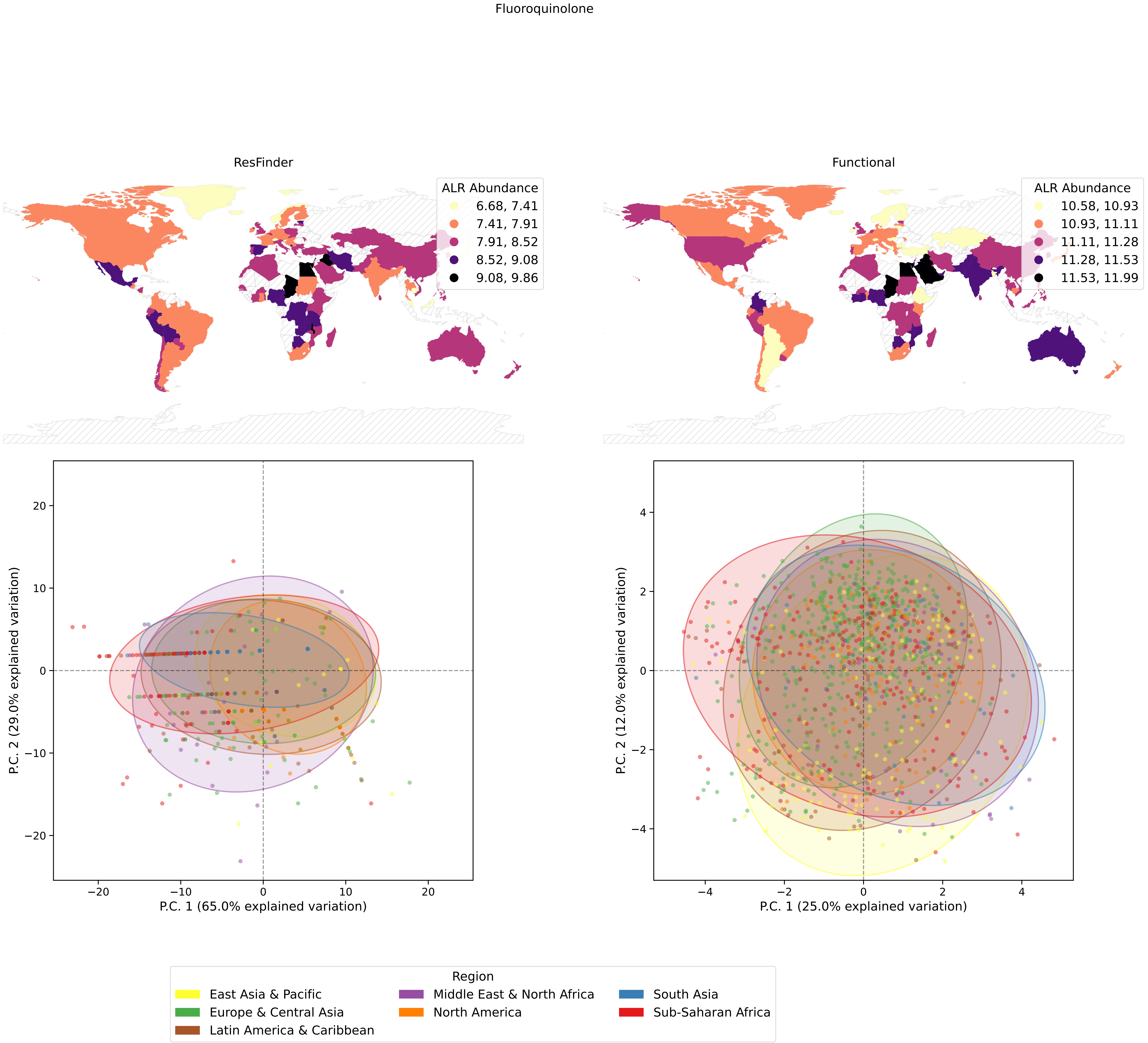

Fluoroquinolone
ResFinder
Functional
ALR Abundance
6.68, 7.41
7.41, 7.91
7.91, 8.52
8.52, 9.08
9.08, 9.86
ALR Abundance
10.58, 10.93
10.93, 11.11
11.11, 11.28
11.28, 11.53
11.53, 11.99
P.C. 1 (65.0% explained variation)
P.C. 2 (29.0% explained variation)
P.C. 1 (25.0% explained variation)
P.C. 2 (12.0% explained variation)
Region
East Asia & Pacific
Europe & Central Asia
Latin America & Caribbean
Middle East & North Africa
North America
South Asia
Sub-Saharan Africa

Folate_Pathway_Antagonist

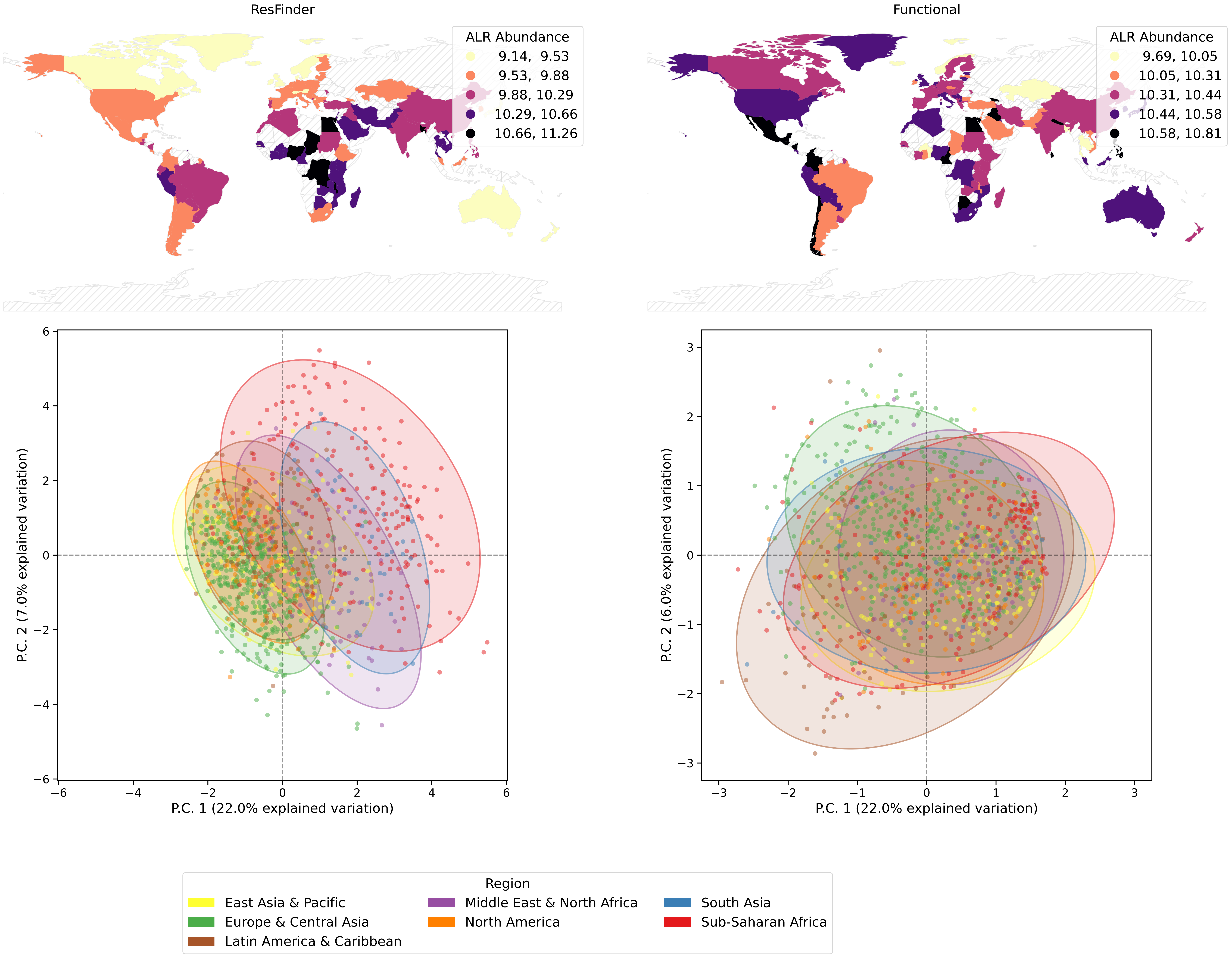

Fosfomycin

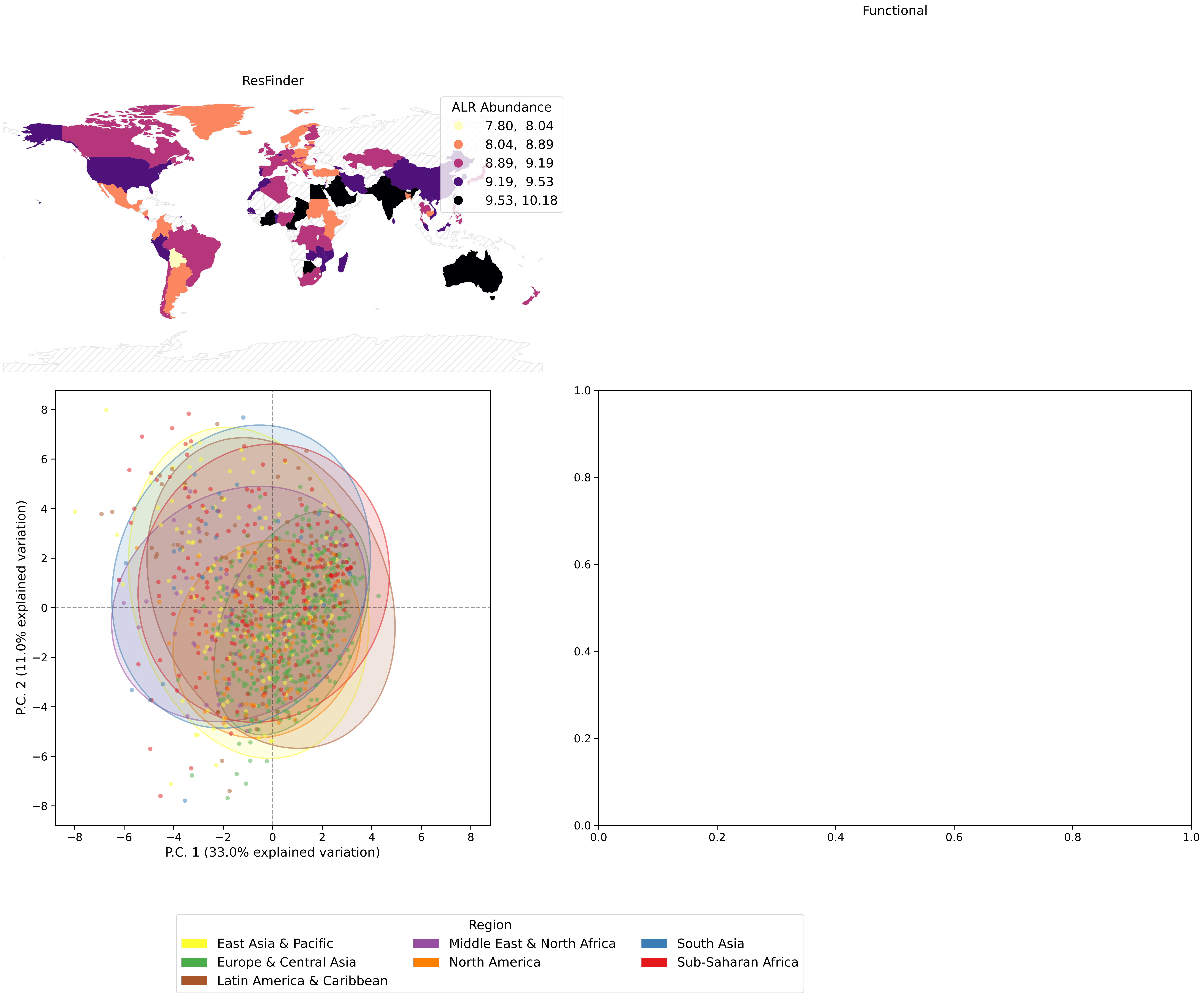

Functional
ResFinder
ALR Abundance
7.80, 8.04
8.04, 8.89
8.89, 9.19
9.19, 9.53
9.53, 10.18
P.C. 2 (11.0% explained variation)
P.C. 1 (33.0% explained variation)
Region
East Asia & Pacific
Europe & Central Asia
Latin America & Caribbean
Middle East & North Africa
North America
South Asia
Sub-Saharan Africa

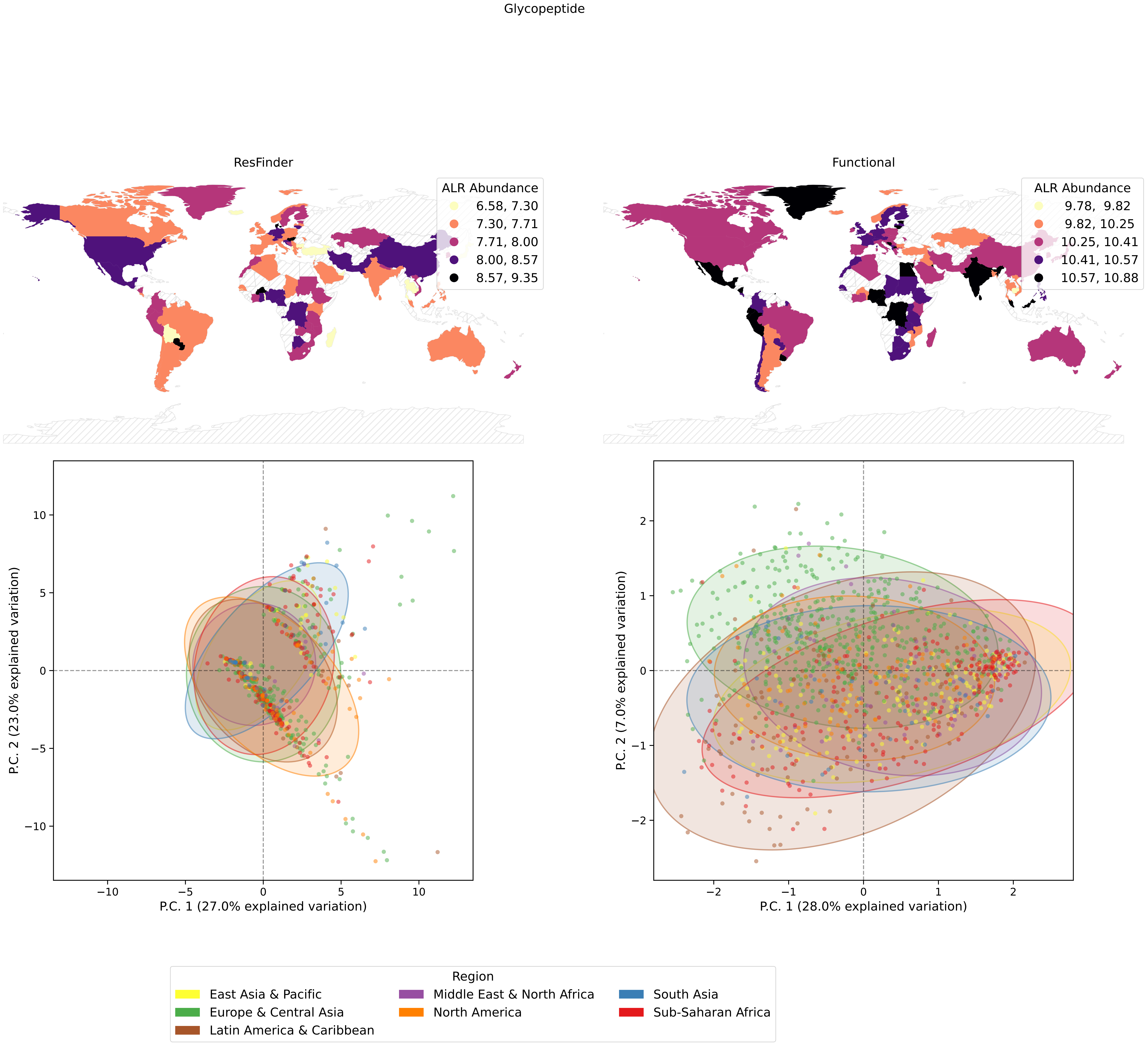
Glycopeptide
ResFinder
Functional
ALR Abundance
6.58, 7.30
7.30, 7.71
7.71, 8.00
8.00, 8.57
8.57, 9.35
ALR Abundance
9.78, 9.82
9.82, 10.25
10.25, 10.41
10.41, 10.57
10.57, 10.88
P.C. 1 (27.0% explained variation)
P.C. 2 (23.0% explained variation)
P.C. 1 (28.0% explained variation)
P.C. 2 (7.0% explained variation)
Region
East Asia & Pacific
Europe & Central Asia
Latin America & Caribbean
Middle East & North Africa
North America
South Asia
Sub-Saharan Africa

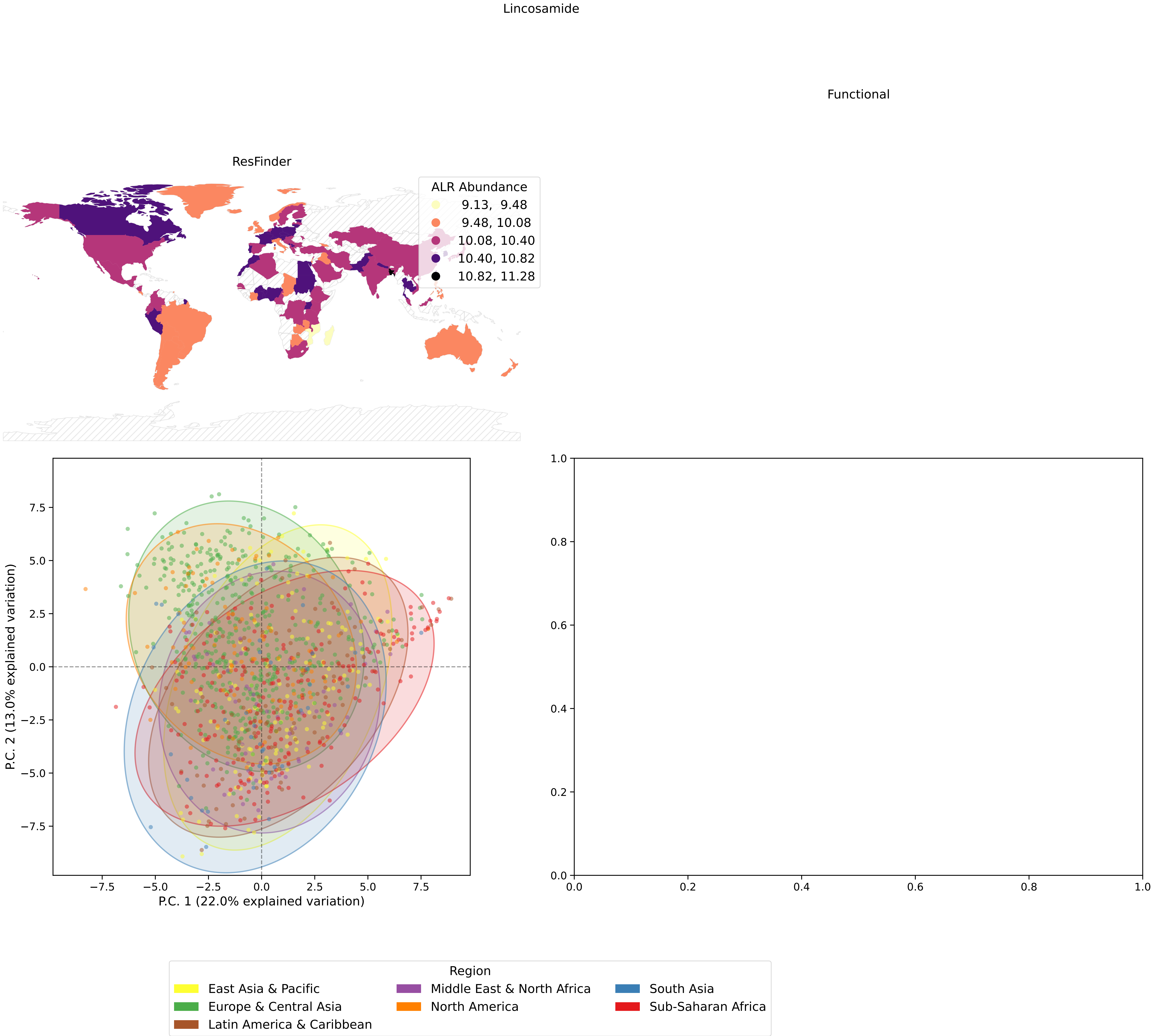
Lincosamide
Functional
ResFinder
ALR Abundance
9.13, 9.48
9.48, 10.08
10.08, 10.40
10.40, 10.82
10.82, 11.28
P.C. 2 (13.0% explained variation)
P.C. 1 (22.0% explained variation)
−7.5
−5.0
−2.5
0.0
2.5
5.0
7.5
0.0
0.2
0.4
0.6
0.8
1.0
Region
East Asia & Pacific
Europe & Central Asia
Latin America & Caribbean
Middle East & North Africa
North America
South Asia
Sub-Saharan Africa

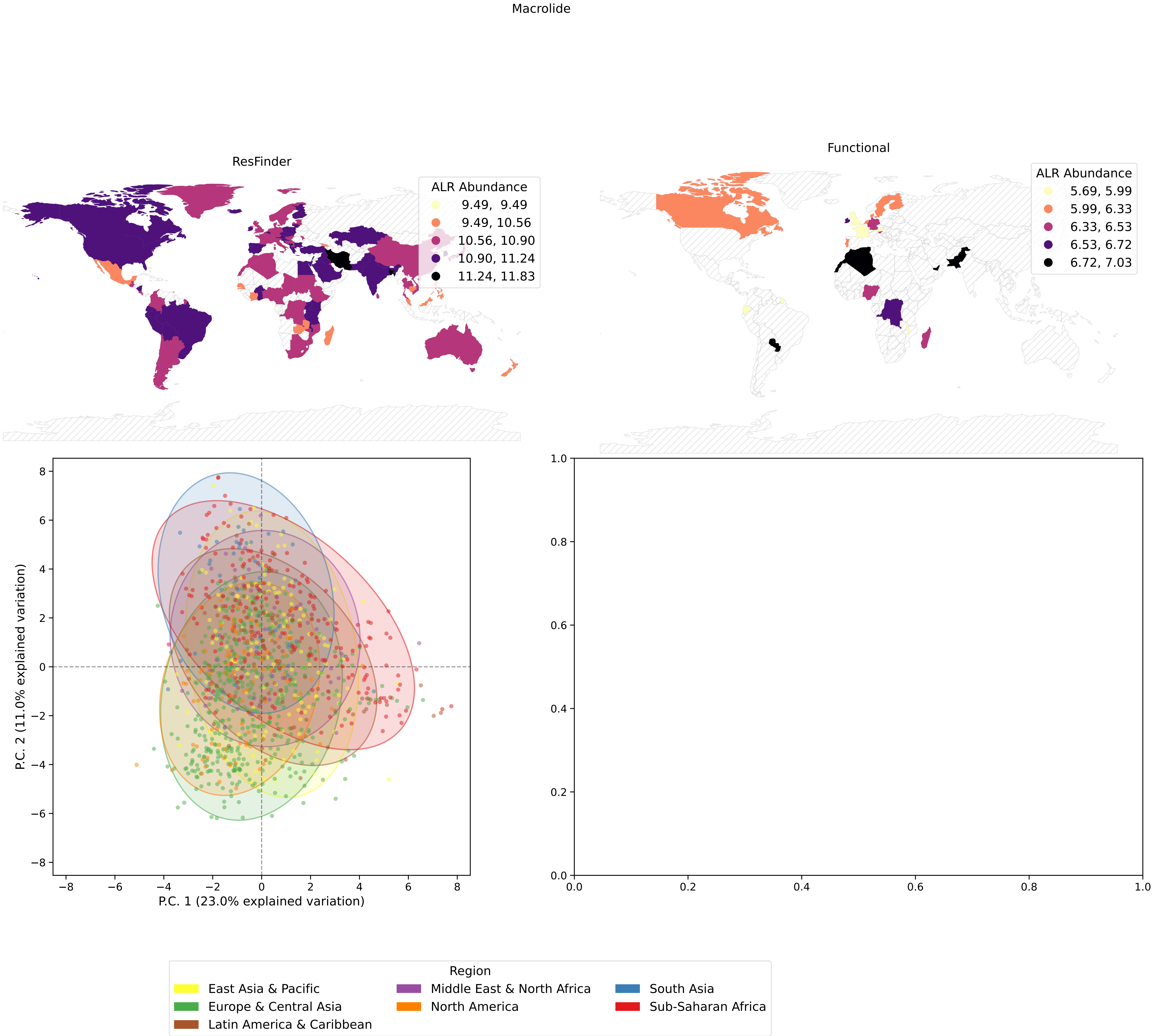
Macrolide
ResFinder
ALR Abundance
9.49, 9.49
9.49, 10.56
10.56, 10.90
10.90, 11.24
11.24, 11.83
Functional
ALR Abundance
5.69, 5.99
5.99, 6.33
6.33, 6.53
6.53, 6.72
6.72, 7.03
P.C. 1 (23.0% explained variation)
P.C. 2 (11.0% explained variation)
Region
East Asia & Pacific
Europe & Central Asia
Latin America & Caribbean
Middle East & North Africa
North America
South Asia
Sub-Saharan Africa

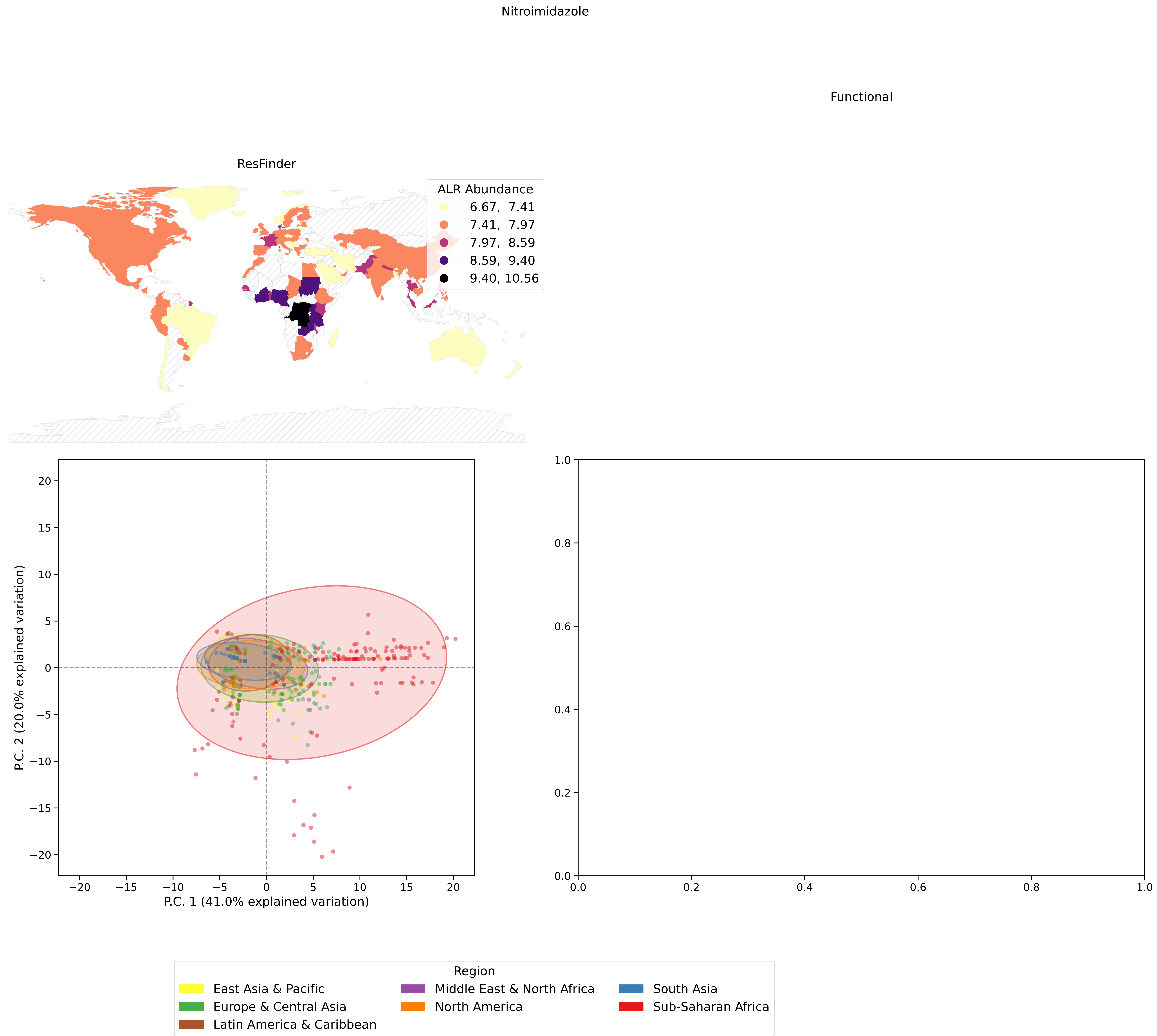
Nitroimidazole
Functional
ResFinder
ALR Abundance
6.67, 7.41
7.41, 7.97
7.97, 8.59
8.59, 9.40
9.40, 10.56
P.C. 1 (41.0% explained variation)
P.C. 2 (20.0% explained variation)
Region
East Asia & Pacific
Europe & Central Asia
Latin America & Caribbean
Middle East & North Africa
North America
South Asia
Sub-Saharan Africa

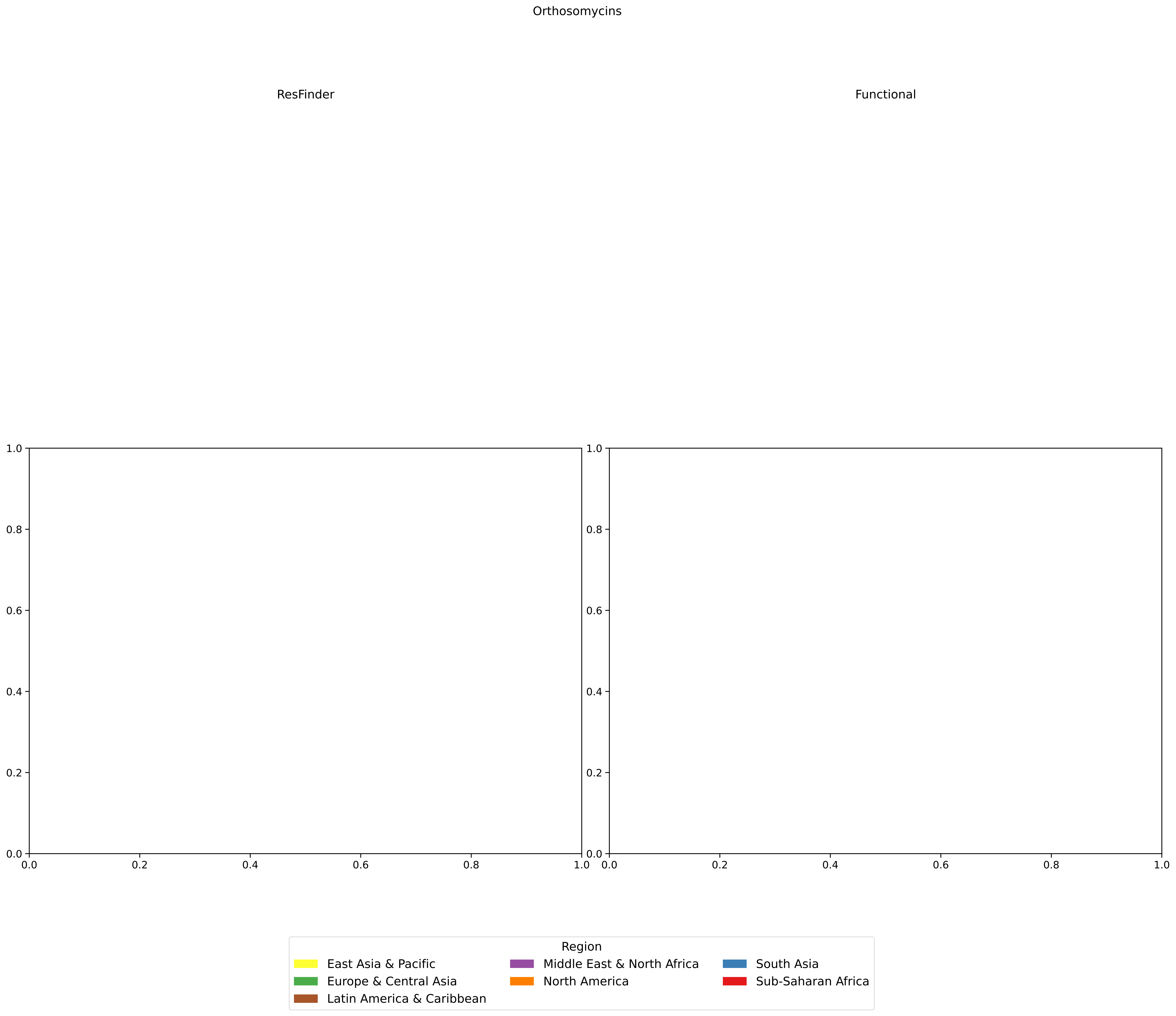
Orthosomycins
ResFinder
Functional
1.0
0.8
0.6
0.4
0.2
0.0
0.0
0.2
0.4
0.6
0.8
1.0
Region
East Asia & Pacific
Europe & Central Asia
Latin America & Caribbean
Middle East & North Africa
North America
South Asia
Sub-Saharan Africa

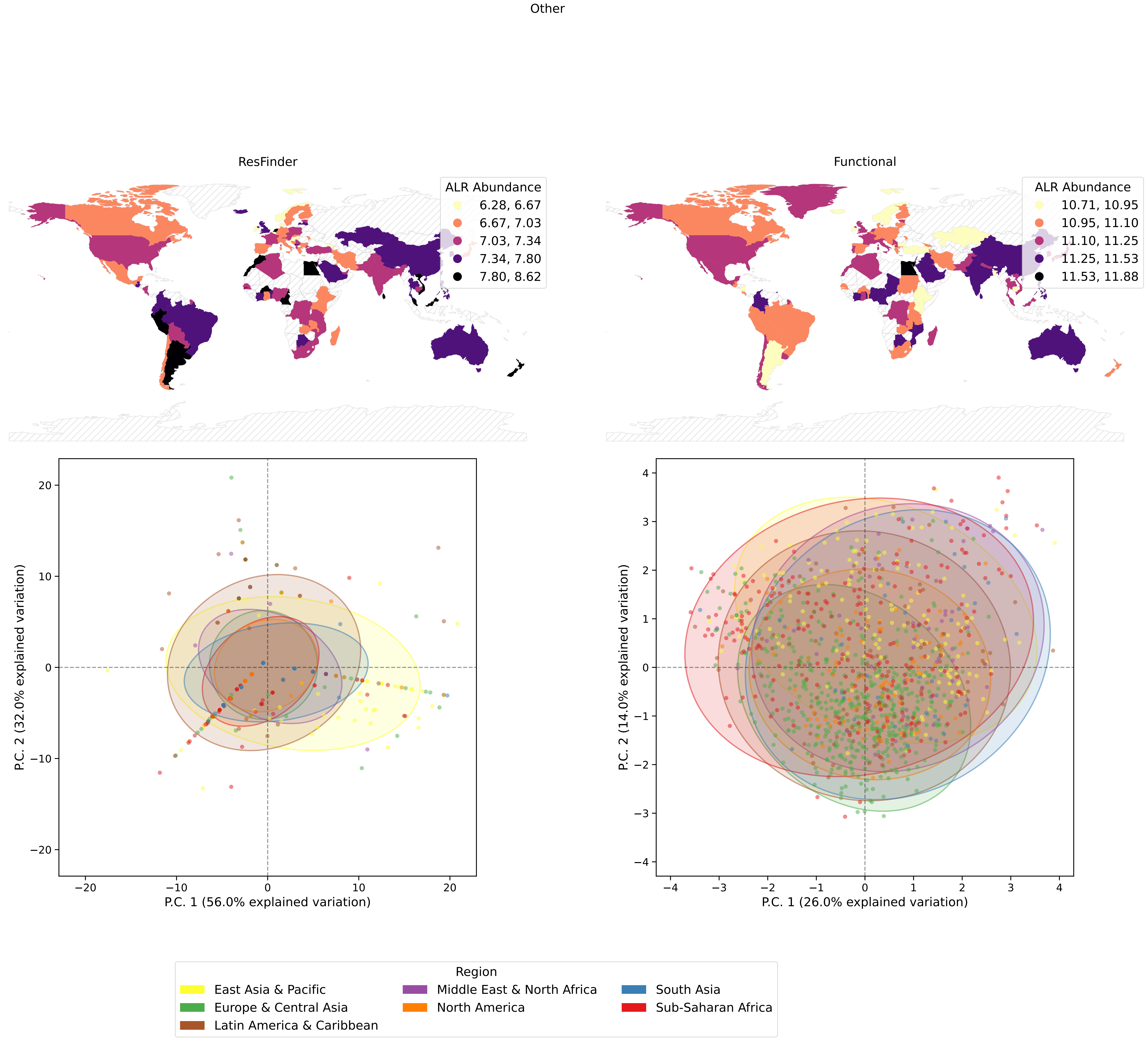

Other
ResFinder
Functional
ALR Abundance
6.28, 6.67
6.67, 7.03
7.03, 7.34
7.34, 7.80
7.80, 8.62
ALR Abundance
10.71, 10.95
10.95, 11.10
11.10, 11.25
11.25, 11.53
11.53, 11.88
P.C. 1 (56.0% explained variation)
P.C. 2 (32.0% explained variation)
P.C. 1 (26.0% explained variation)
P.C. 2 (14.0% explained variation)
Region
East Asia & Pacific
Europe & Central Asia
Latin America & Caribbean
Middle East & North Africa
North America
South Asia
Sub-Saharan Africa

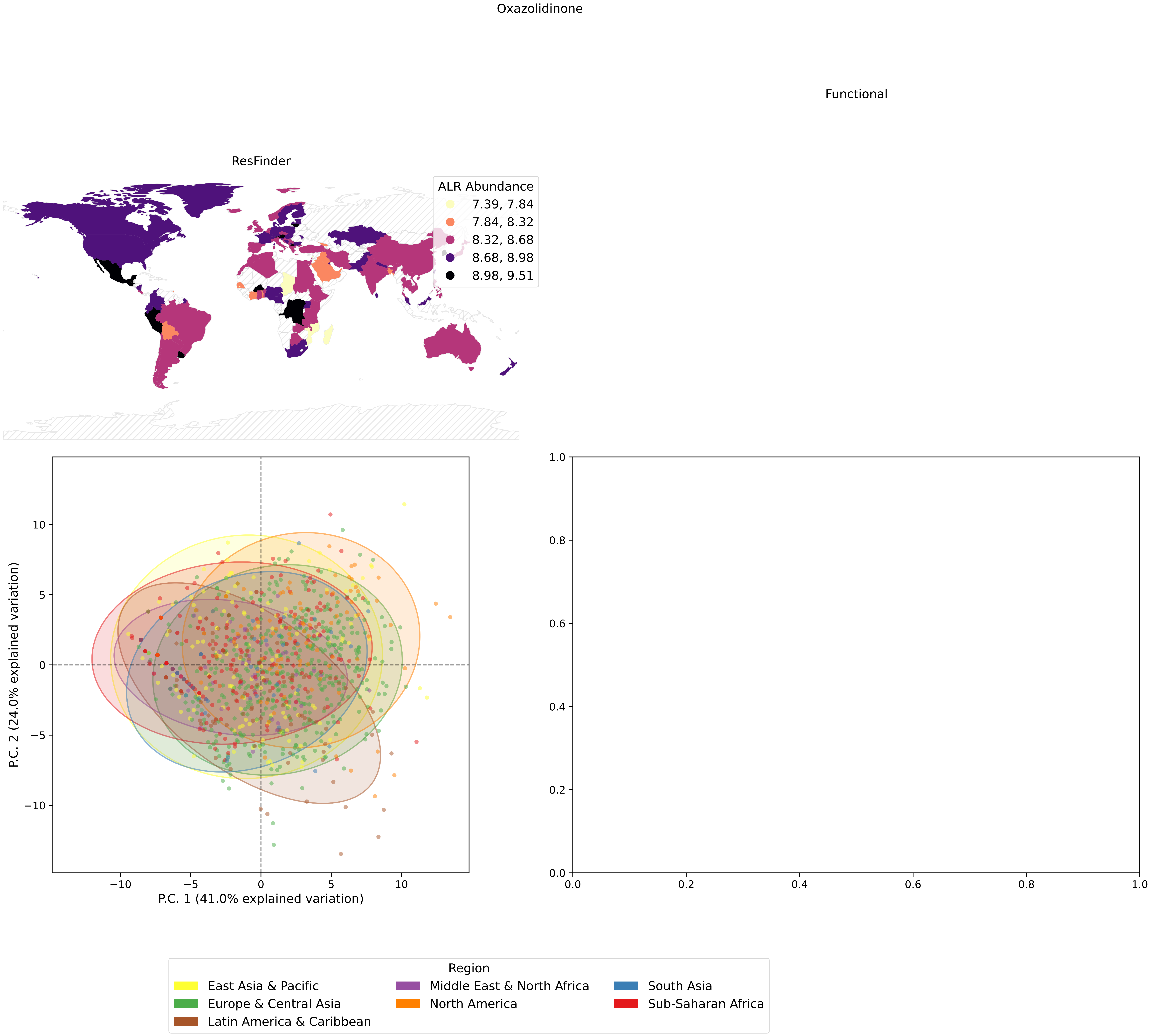

Oxazolidinone
Functional
ResFinder
ALR Abundance
7.39, 7.84
7.84, 8.32
8.32, 8.68
8.68, 8.98
8.98, 9.51
P.C. 1 (41.0% explained variation)
P.C. 2 (24.0% explained variation)
Region
East Asia & Pacific
Europe & Central Asia
Latin America & Caribbean
Middle East & North Africa
North America
South Asia
Sub-Saharan Africa

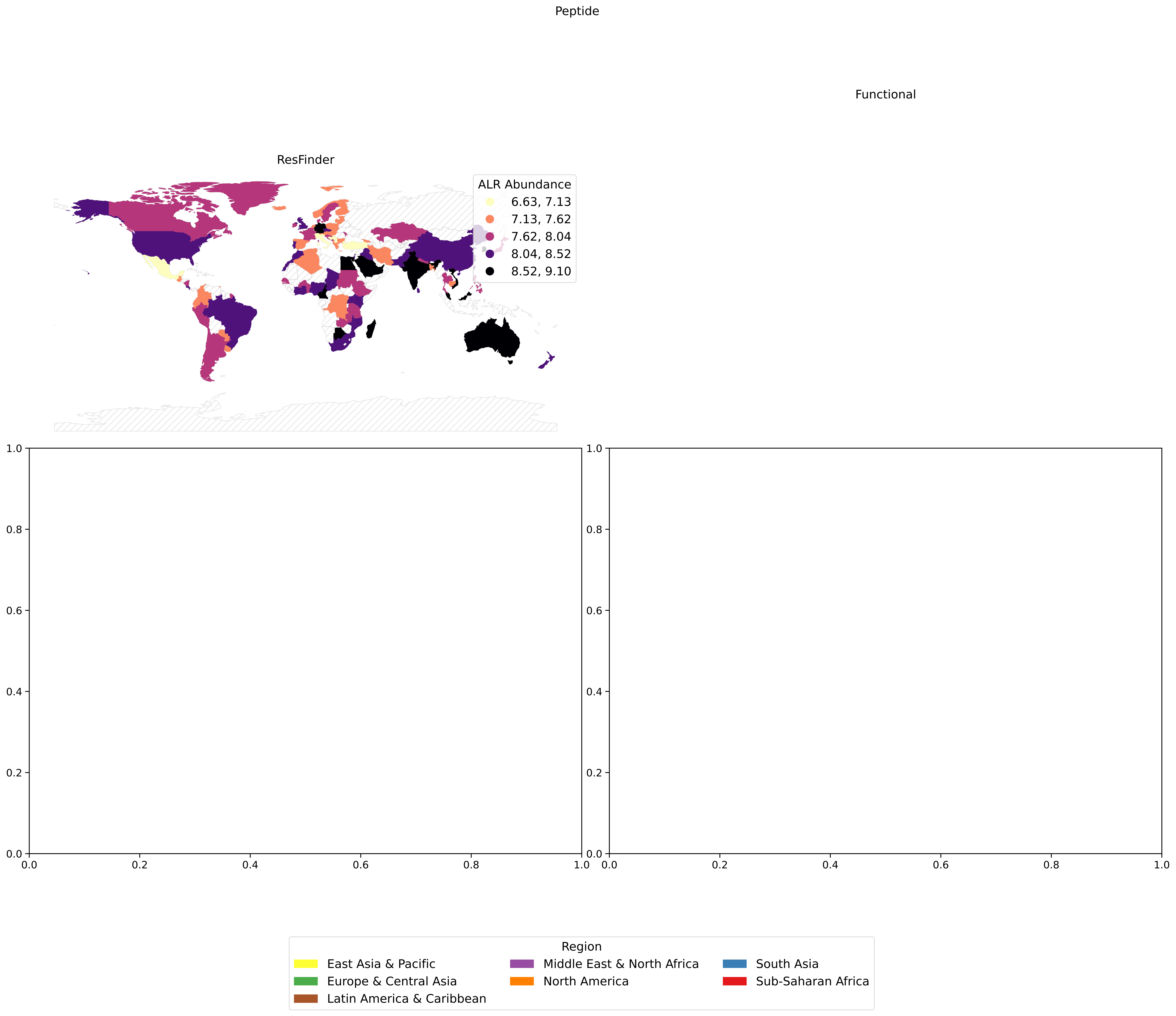
Peptide
Functional
ResFinder
ALR Abundance
6.63, 7.13
7.13, 7.62
7.62, 8.04
8.04, 8.52
8.52, 9.10
0.0
0.2
0.4
0.6
0.8
1.0
Region
East Asia & Pacific
Europe & Central Asia
Latin America & Caribbean
Middle East & North Africa
North America
South Asia
Sub-Saharan Africa

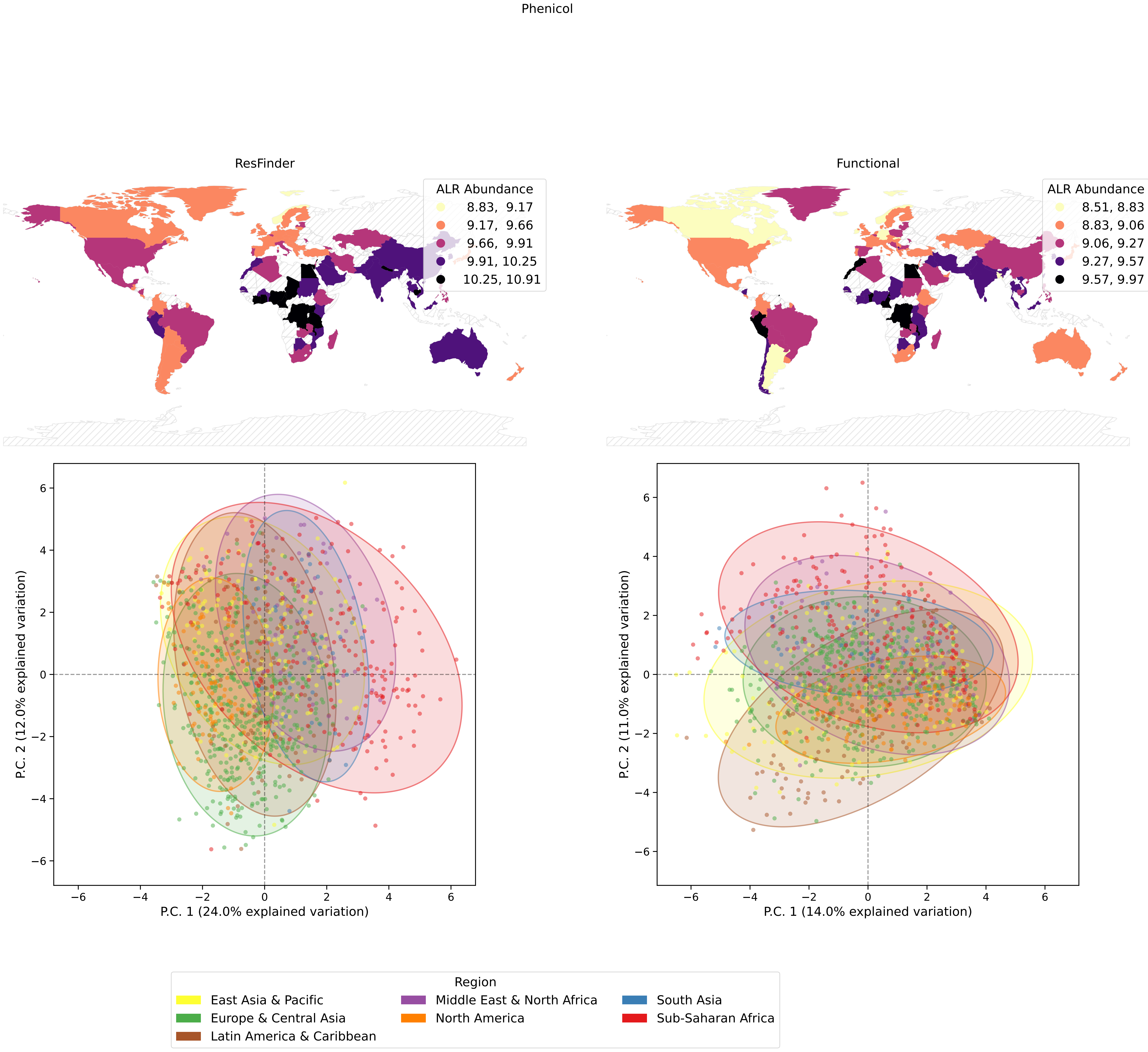

Phenicol
ResFinder
Functional
ALR Abundance
8.83, 9.17
9.17, 9.66
9.66, 9.91
9.91, 10.25
10.25, 10.91
ALR Abundance
8.51, 8.83
8.83, 9.06
9.06, 9.27
9.27, 9.57
9.57, 9.97
P.C. 1 (24.0% explained variation)
P.C. 2 (12.0% explained variation)
P.C. 1 (14.0% explained variation)
P.C. 2 (11.0% explained variation)
Region
East Asia & Pacific
Europe & Central Asia
Latin America & Caribbean
Middle East & North Africa
North America
South Asia
Sub-Saharan Africa

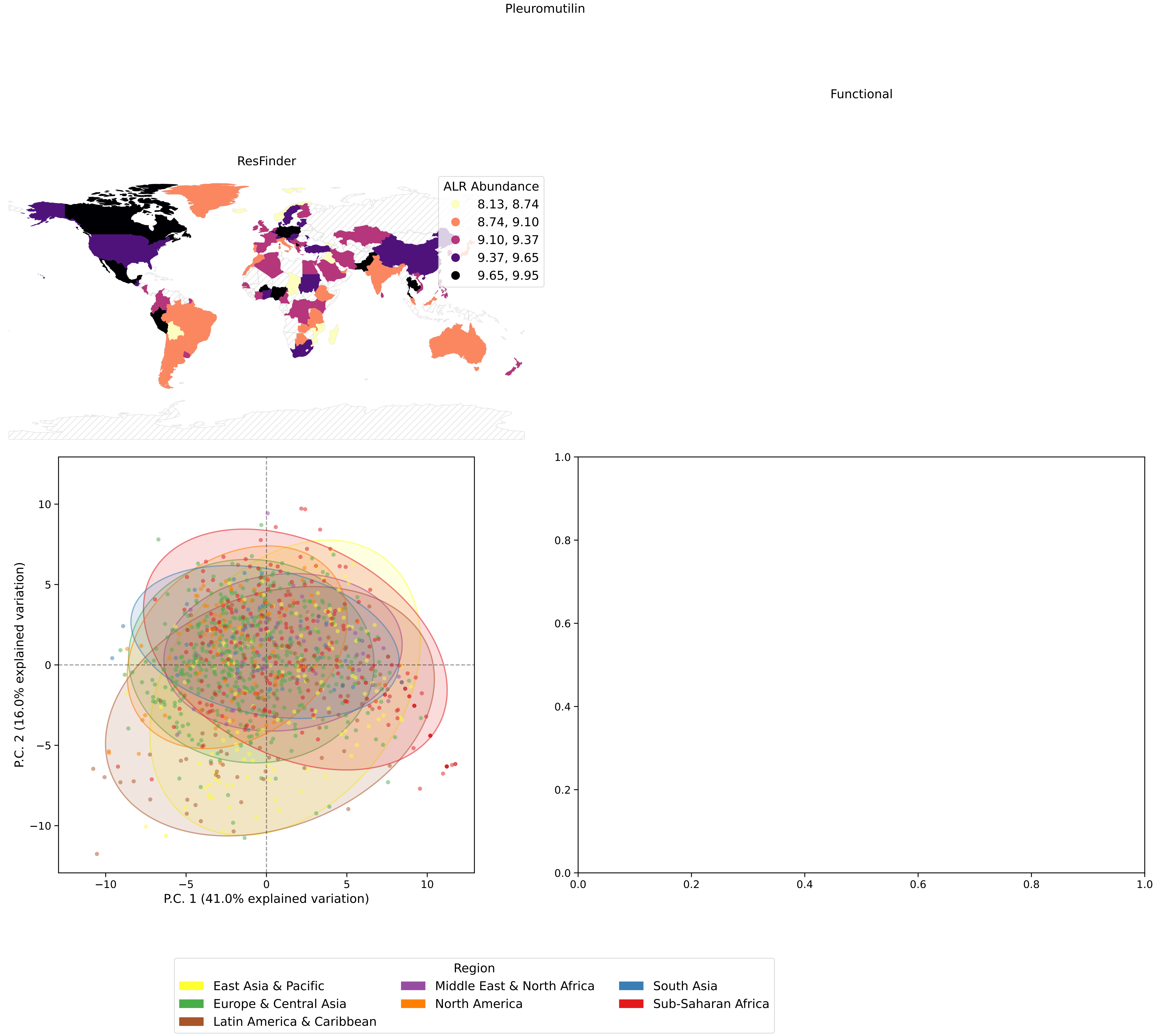
Pleuromutilin
ResFinder
Functional
ALR Abundance
8.13, 8.74
8.74, 9.10
9.10, 9.37
9.37, 9.65
9.65, 9.95
P.C. 1 (41.0% explained variation)
P.C. 2 (16.0% explained variation)
Region
East Asia & Pacific
Europe & Central Asia
Latin America & Caribbean
Middle East & North Africa
North America
South Asia
Sub-Saharan Africa

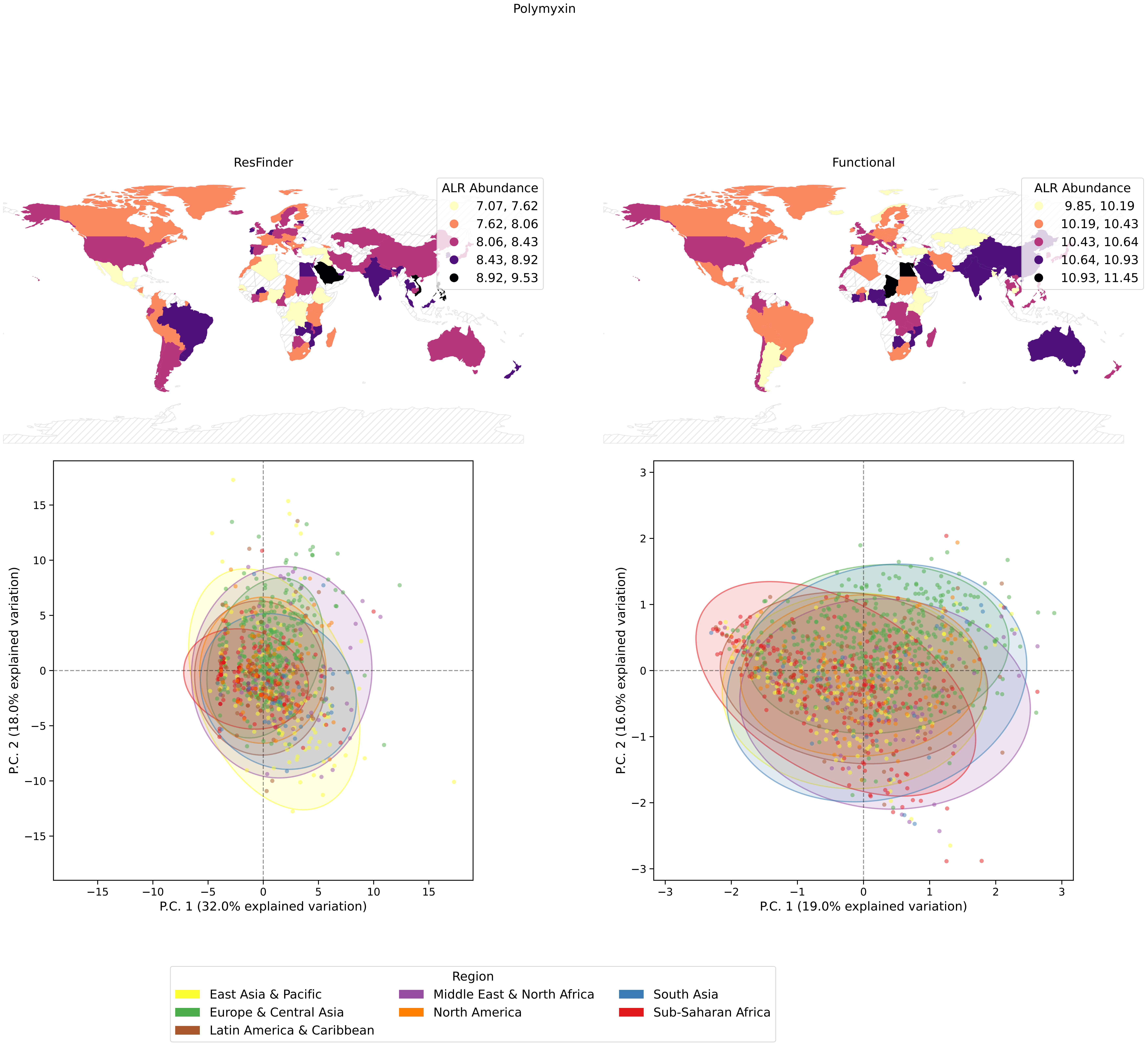
Polymyxin
ResFinder
ALR Abundance
7.07, 7.62
7.62, 8.06
8.06, 8.43
8.43, 8.92
8.92, 9.53
Functional
ALR Abundance
9.85, 10.19
10.19, 10.43
10.43, 10.64
10.64, 10.93
10.93, 11.45
P.C. 1 (32.0% explained variation)
P.C. 2 (18.0% explained variation)
P.C. 1 (19.0% explained variation)
P.C. 2 (16.0% explained variation)
Region
East Asia & Pacific
Europe & Central Asia
Latin America & Caribbean
Middle East & North Africa
North America
South Asia
Sub-Saharan Africa

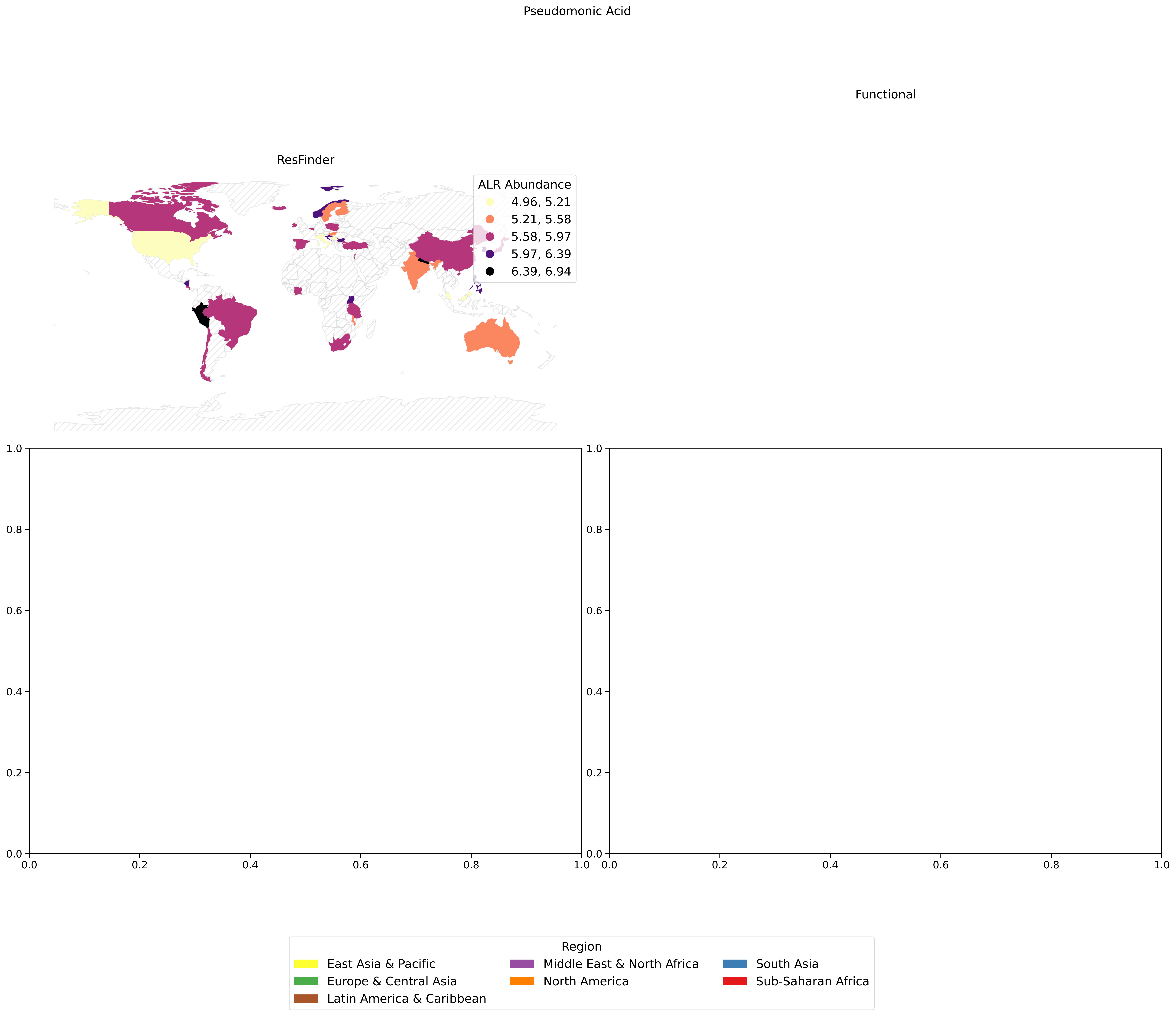
Pseudomonic Acid
Functional
ResFinder
ALR Abundance
4.96, 5.21
5.21, 5.58
5.58, 5.97
5.97, 6.39
6.39, 6.94
0.0
0.2
0.4
0.6
0.8
1.0
Region
East Asia & Pacific
Europe & Central Asia
Latin America & Caribbean
Middle East & North Africa
North America
South Asia
Sub-Saharan Africa

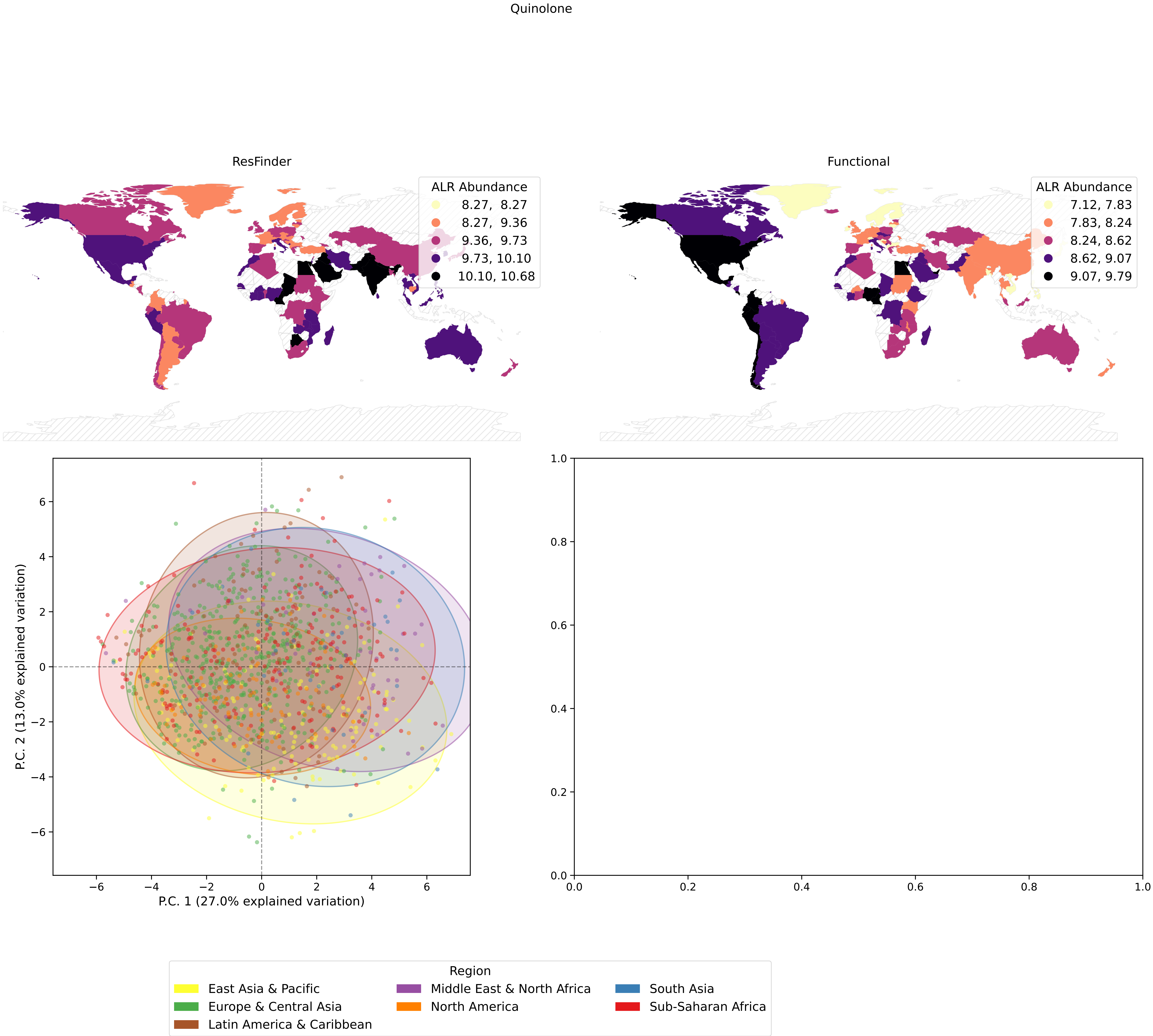

Quinolone
ResFinder
ALR Abundance
8.27, 8.27
8.27, 9.36
9.36, 9.73
9.73, 10.10
10.10, 10.68
Functional
ALR Abundance
7.12, 7.83
7.83, 8.24
8.24, 8.62
8.62, 9.07
9.07, 9.79
P.C. 1 (27.0% explained variation)
P.C. 2 (13.0% explained variation)
Region
East Asia & Pacific
Europe & Central Asia
Latin America & Caribbean
Middle East & North Africa
North America
South Asia
Sub-Saharan Africa

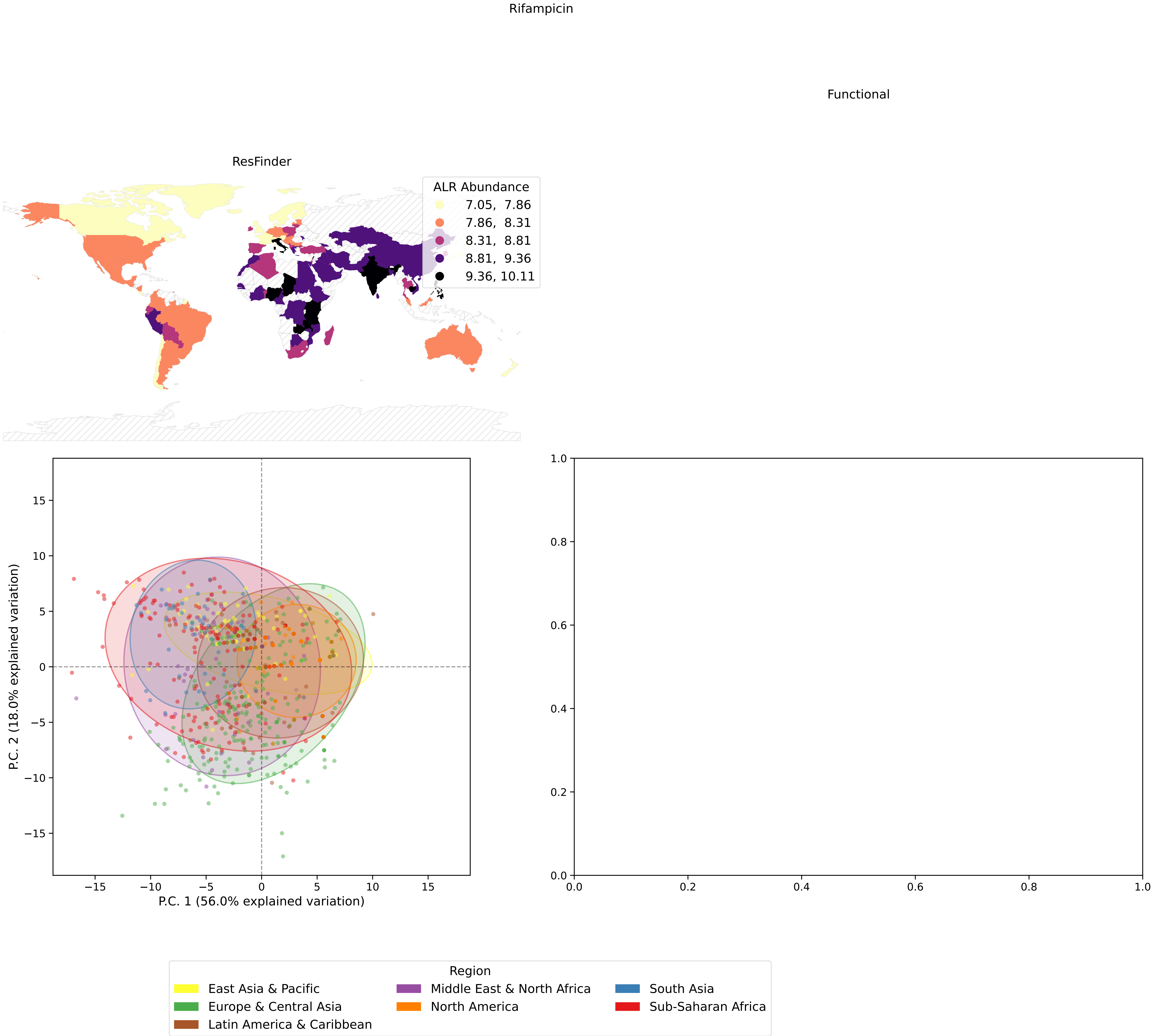
Rifampicin
Functional
ResFinder
ALR Abundance
7.05, 7.86
7.86, 8.31
8.31, 8.81
8.81, 9.36
9.36, 10.11
P.C. 1 (56.0% explained variation)
P.C. 2 (18.0% explained variation)
Region
East Asia & Pacific
Europe & Central Asia
Latin America & Caribbean
Middle East & North Africa
North America
South Asia
Sub-Saharan Africa

Steroid_Antibacterial

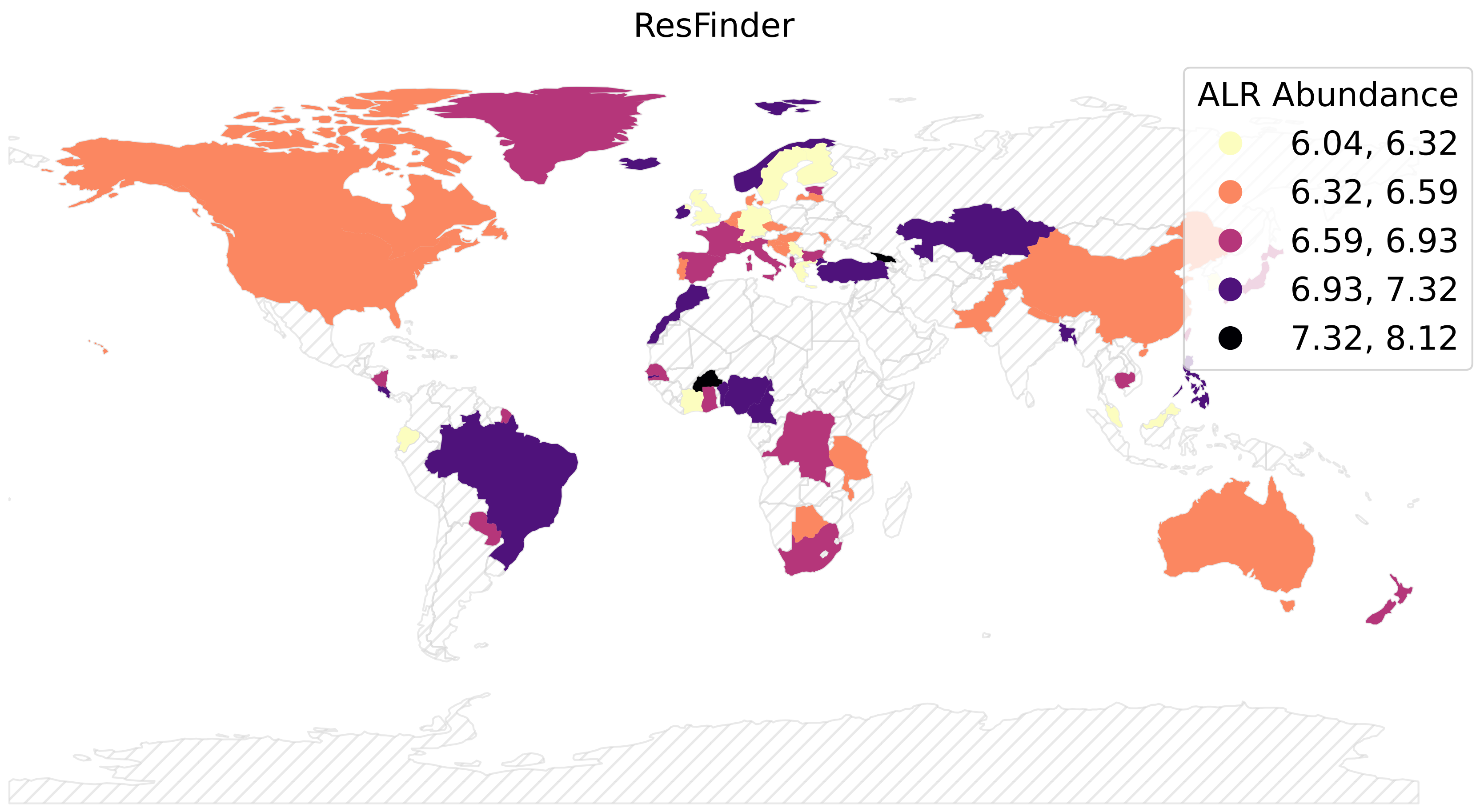

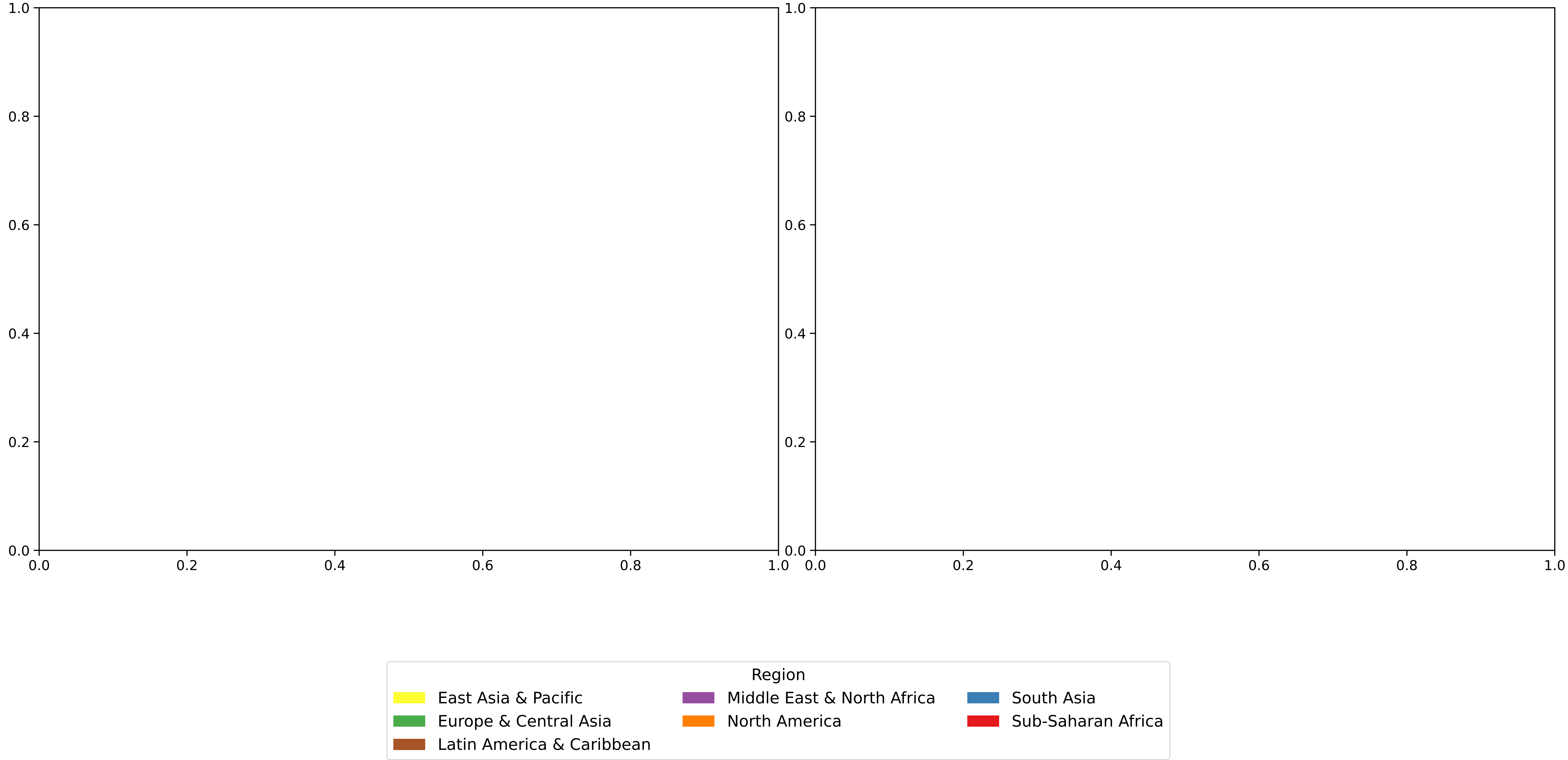

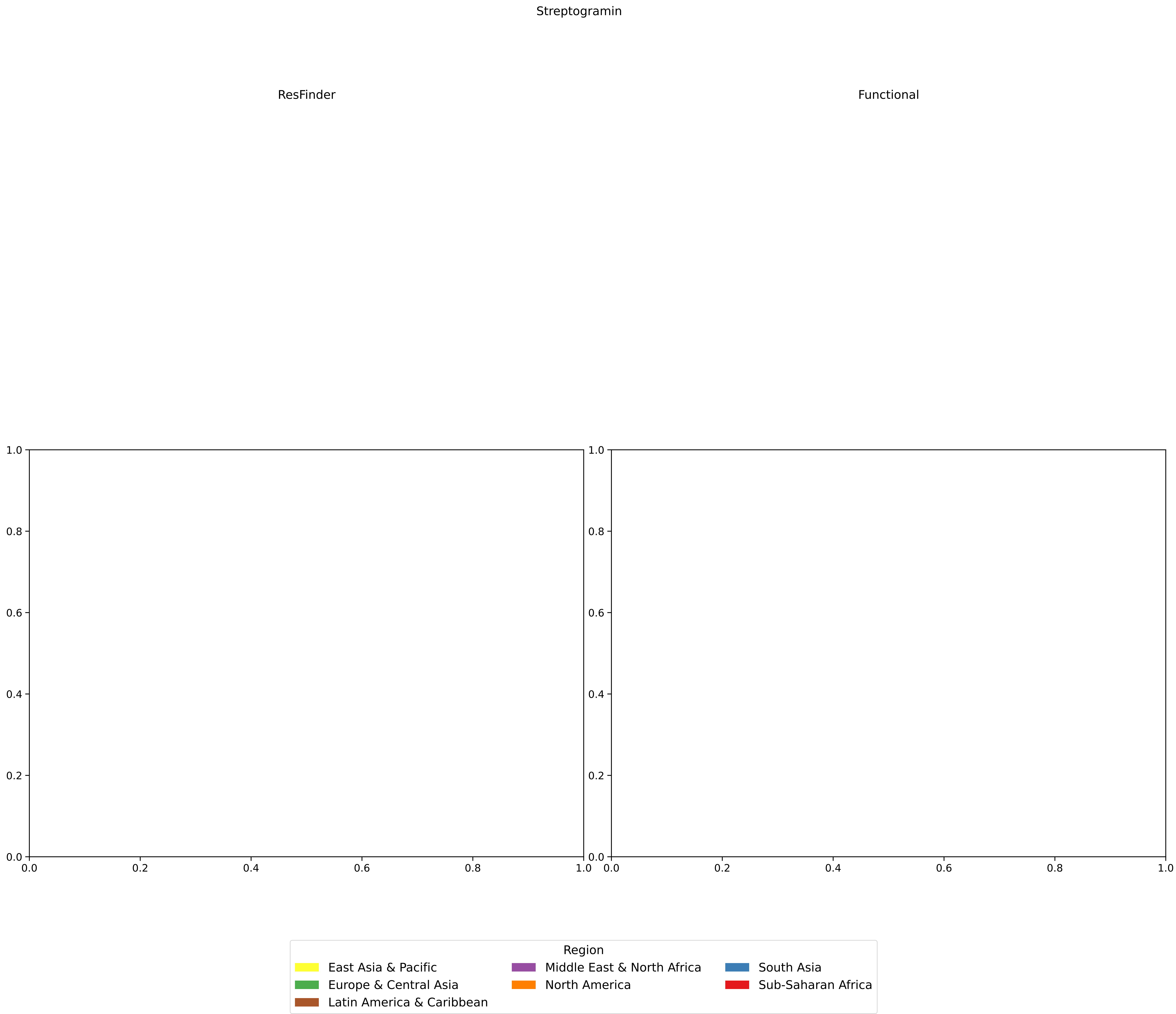
Streptogramin
ResFinder
Functional
0.0
0.2
0.4
0.6
0.8
1.0
Region
East Asia & Pacific
Europe & Central Asia
Latin America & Caribbean
Middle East & North Africa
North America
South Asia
Sub-Saharan Africa

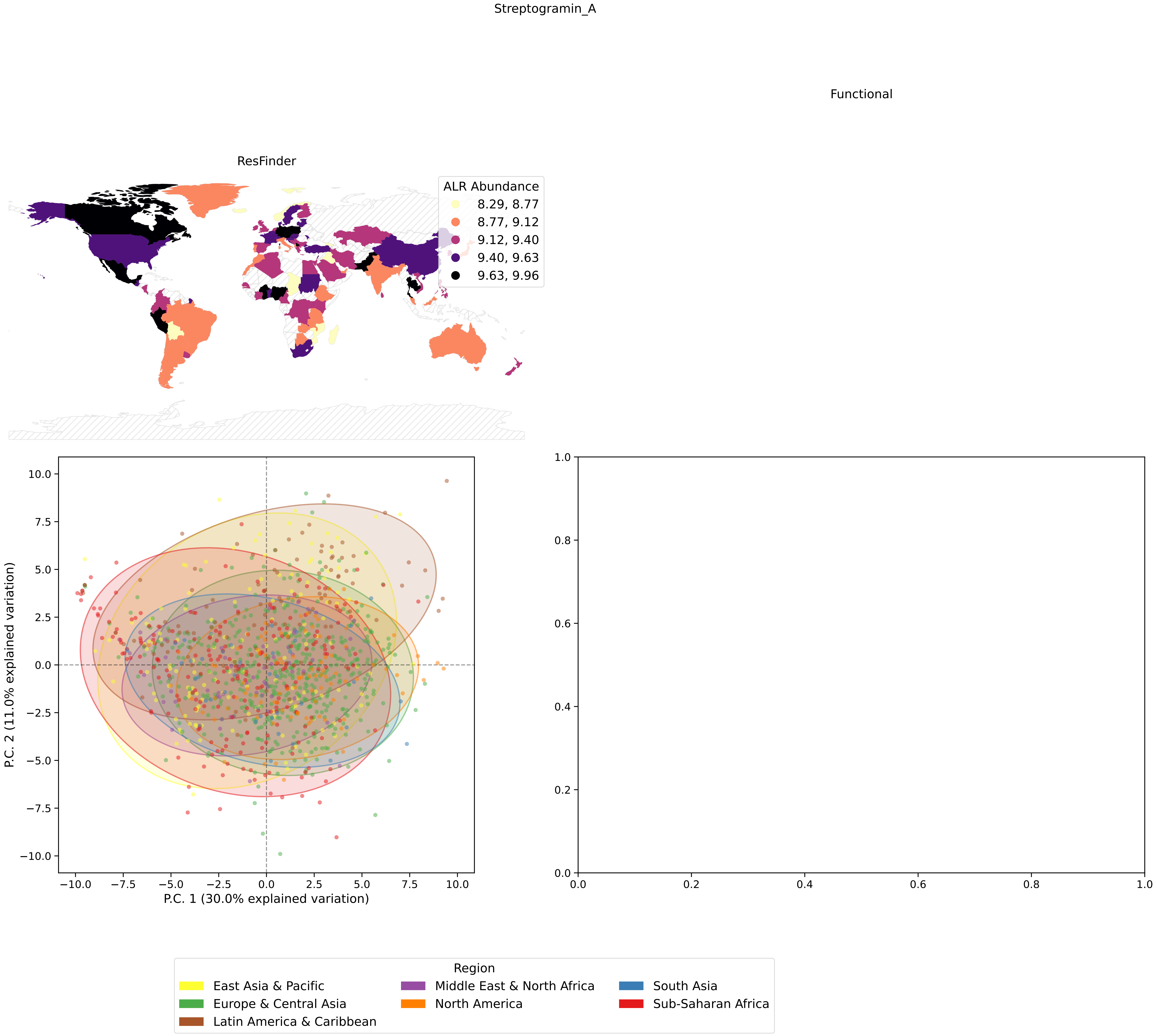

Streptogramin_A
Functional
ResFinder
ALR Abundance
8.29, 8.77
8.77, 9.12
9.12, 9.40
9.40, 9.63
9.63, 9.96
P.C. 2 (11.0% explained variation)
P.C. 1 (30.0% explained variation)
−10.0 −7.5 −5.0 −2.5 0.0 2.5 5.0 7.5 10.0
0.0 0.2 0.4 0.6 0.8 1.0
Region
East Asia & Pacific
Europe & Central Asia
Latin America & Caribbean
Middle East & North Africa
North America
South Asia
Sub-Saharan Africa

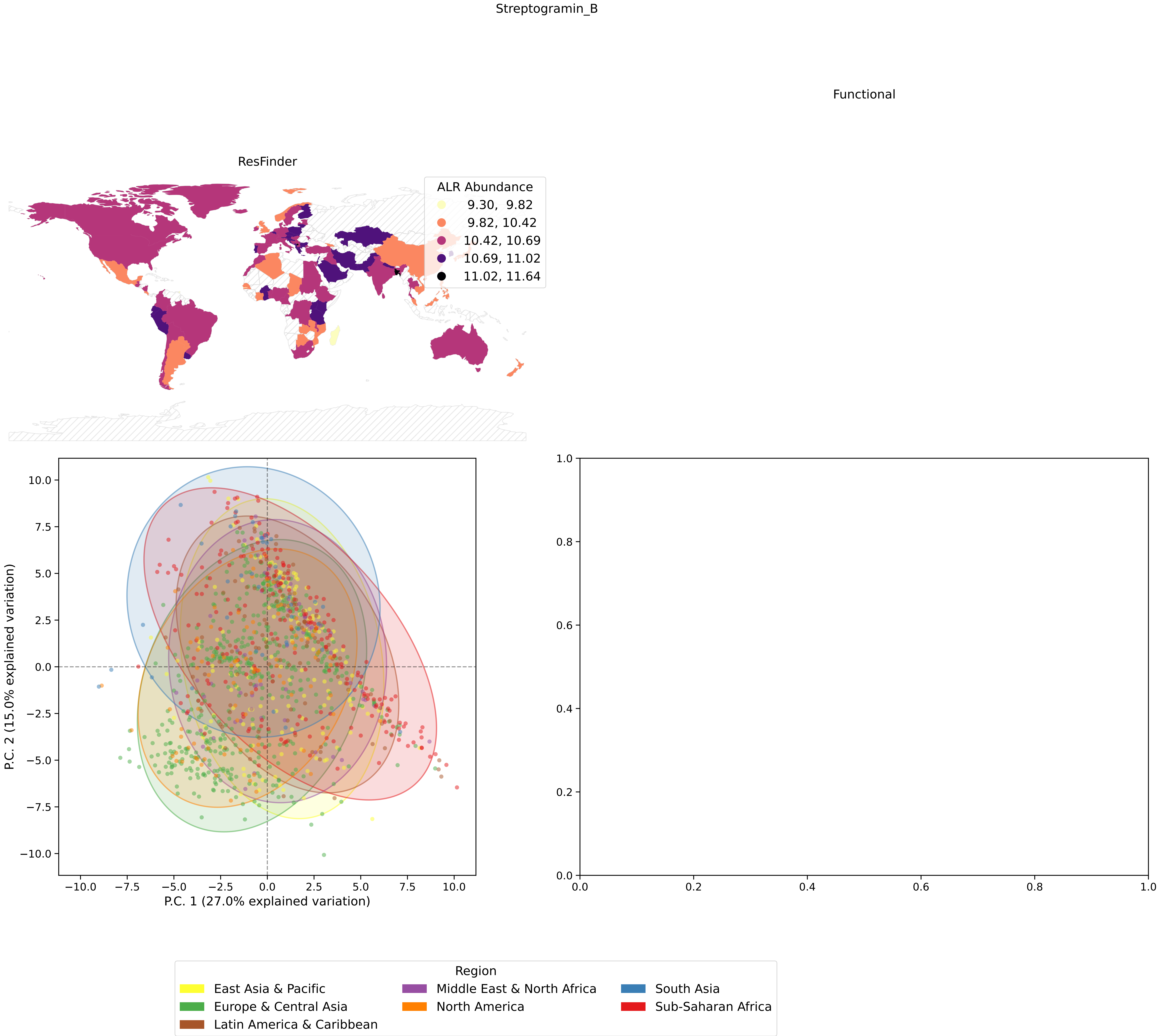
Streptogramin_B
Functional
ResFinder
ALR Abundance
9.30, 9.82
9.82, 10.42
10.42, 10.69
10.69, 11.02
11.02, 11.64
P.C. 1 (27.0% explained variation)
P.C. 2 (15.0% explained variation)
Region
East Asia & Pacific
Europe & Central Asia
Latin America & Caribbean
Middle East & North Africa
North America
South Asia
Sub-Saharan Africa

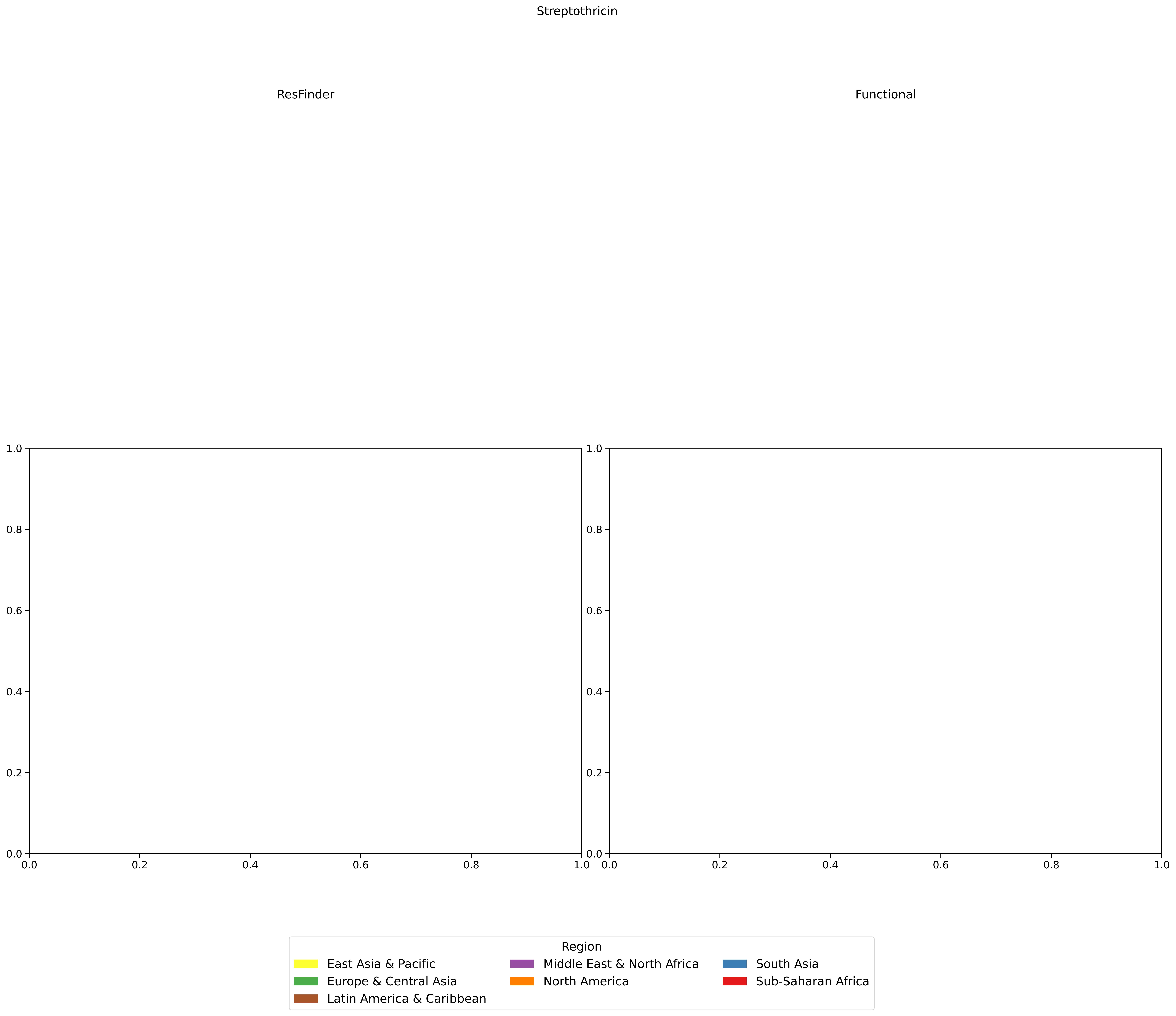
Streptothricin
ResFinder
Functional
1.0
0.8
0.6
0.4
0.2
0.0
0.0
0.2
0.4
0.6
0.8
1.0
Region
East Asia & Pacific
Europe & Central Asia
Latin America & Caribbean
Middle East & North Africa
North America
South Asia
Sub-Saharan Africa

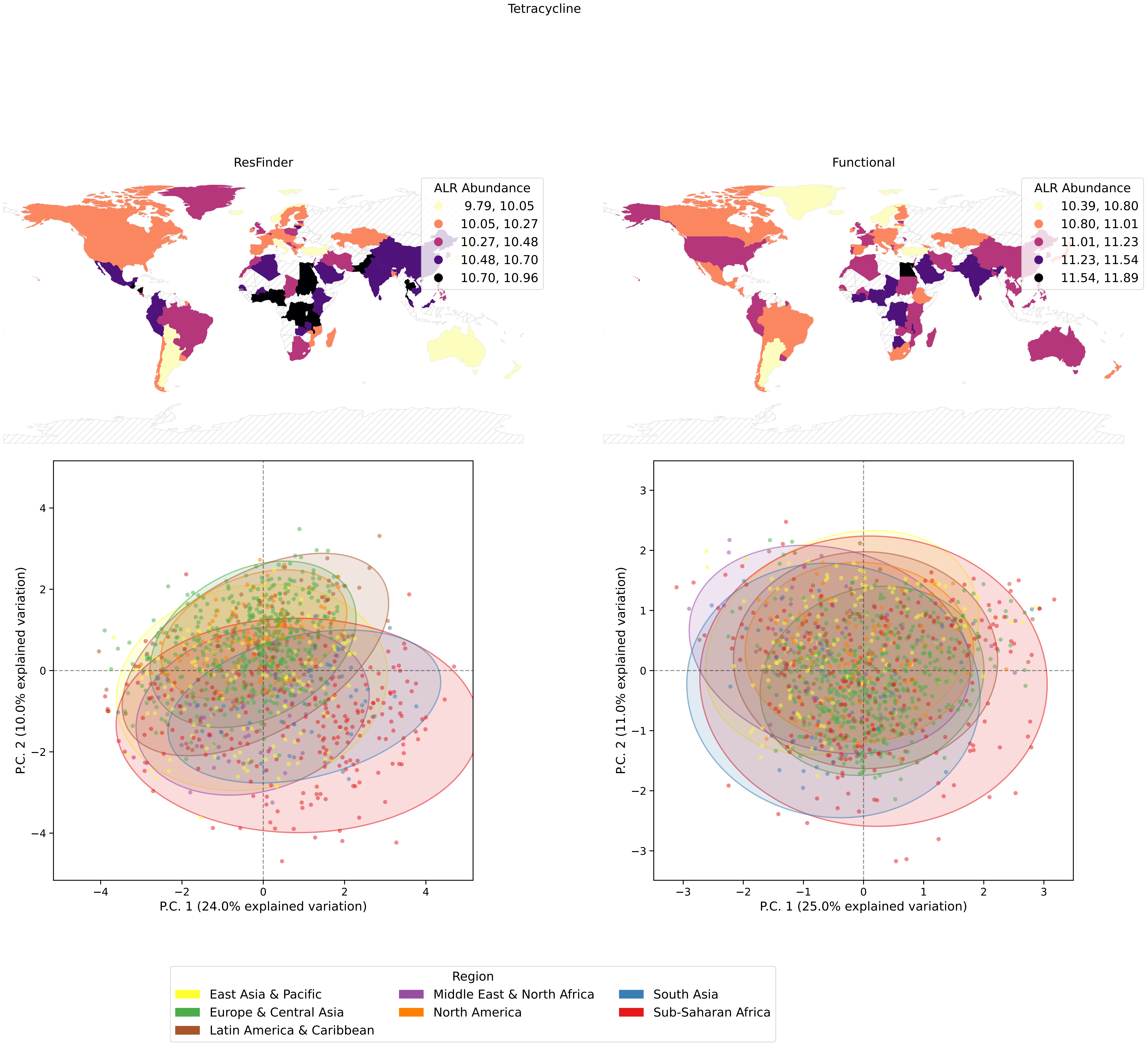

Tetracycline
ResFinder
Functional
ALR Abundance
9.79, 10.05
10.05, 10.27
10.27, 10.48
10.48, 10.70
10.70, 10.96
ALR Abundance
10.39, 10.80
10.80, 11.01
11.01, 11.23
11.23, 11.54
11.54, 11.89
P.C. 1 (24.0% explained variation)
P.C. 2 (10.0% explained variation)
P.C. 1 (25.0% explained variation)
P.C. 2 (11.0% explained variation)
Region
East Asia & Pacific
Europe & Central Asia
Latin America & Caribbean
Middle East & North Africa
North America
South Asia
Sub-Saharan Africa
